# Non-Canonical and Sexually Dimorphic X Dosage Compensation States in the Mouse and Human Germline

**DOI:** 10.1016/j.devcel.2016.12.023

**Published:** 2017-02-06

**Authors:** Mahesh N. Sangrithi, Helene Royo, Shantha K. Mahadevaiah, Obah Ojarikre, Leena Bhaw, Abdul Sesay, Antoine H.F.M. Peters, Michael Stadler, James M.A. Turner

**Affiliations:** 1 Mill Hill Laboratory, The Francis Crick Institute, The Ridgeway, Mill Hill, London NW7 1AA, UK; 2 Friedrich Miescher Institute for Biomedical Research (FMI), 4058 Basel, Switzerland; 3 Swiss Institute of Bioinformatics, 4058 Basel, Switzerland; 4 UCL EGA Institute for Women's Health UCL, Medical School Building, 74 Huntley Street, London WC1E 6AU, UK

**Keywords:** sex chromosomes, dosage compensation, germline development, genome-wide reprogramming, X inactivation, X upregulation

## Abstract

Somatic X dosage compensation requires two mechanisms: X inactivation balances X gene output between males (XY) and females (XX), while X upregulation, hypothesized by Ohno and documented in vivo, balances X gene with autosomal gene output. Whether X dosage compensation occurs in germ cells is unclear. We show that mouse and human germ cells exhibit non-canonical X dosage states that differ from the soma and between the sexes. Prior to genome-wide reprogramming, X upregulation is present, consistent with Ohno's hypothesis. Subsequently, however, it is erased. In females, erasure follows loss of X inactivation, causing X dosage excess. Conversely, in males, erasure leads to permanent X dosage decompensation. Sex chromosomally abnormal models exhibit a “sex-reversed” X dosage state: XX males, like XX females, develop X dosage excess, while XO females, like XY males, develop X dosage decompensation. Thus, germline X dosage compensation states are determined by X chromosome number, not phenotypic sex. These unexpected differences in X dosage compensation states between germline and soma offer unique perspectives on sex chromosome infertility.

## Highlights

• X dosage compensation in germ cells is reset during GWR
• PGCs exhibit X upregulation before GWR, in keeping with Ohno's hypothesis
• X upregulation is lost during GWR
• Mouse and human germ cells exhibit X dosage states that are sexually dimorphic

## Teaser

Germ cells reset their epigenome and transcriptome prior to meiosis. Sangrithi et al. show that unique X chromosome dosage compensation states prevail in germ cells. These states are determined by the number of X chromosomes present rather than phenotypic sex, providing a different perspective on infertility associated with sex chromosome aneuploidy.

## Introduction

Male and female mammals carry the same complement of autosomes but differ with respect to their sex chromosomes: females have two X chromosomes (XX) while males have one X chromosome and one Y chromosome (XY). The X and Y chromosomes evolved from a pair of ancestral autosomes following the acquisition of the male-determining locus *Sry* on the proto-Y chromosome. The subsequent appearance of sexually antagonistic alleles near *Sry* caused progressive suppression of X-Y recombination (Bachtrog, 2013, Cortez et al., 2014, Hughes and Page, 2015, Muller, 1914). The X chromosome managed to retain most of its ancestral genes through ongoing X-X recombination in the female germline. In contrast, without a partner with which to recombine, the Y chromosome lost most of its original gene content through genetic drift (Charlesworth, 1996, Charlesworth and Charlesworth, 2000).

Evolutionary loss of genes from the Y chromosome led to a disparity in the dosage of X chromosome versus autosomal genes, with males becoming monosomic for X-linked gene products. Susumo Ohno proposed that to rectify this imbalance, expression of X chromosome genes was increased 2-fold to match the output of the diploid autosomal complement, i.e., giving an X-to-autosome ratio (X:A) of 1 (termed Ohno's hypothesis) (Ohno, 1967). This process, X chromosome upregulation, was also acquired in females, leading to a 2-fold excess in X gene expression compared with males. To equalize this resulting sex difference in X gene output, mammals subsequently evolved X chromosome inactivation, the global silencing of one of the two X chromosomes in females (Dupont and Gribnau, 2013, Gendrel and Heard, 2014). Together, X upregulation and X inactivation ensure equalization of gene dosage both within, and between, the sexes.

Consistent with Ohno's hypothesis, X upregulation has been observed in multiple organisms including *Drosophila melanogaster* (Conrad and Akhtar, 2011, Gelbart and Kuroda, 2009, Straub and Becker, 2007), *Caenorhabditis elegans* (Gupta et al., 2006), and mammals (Adler et al., 1997, Gupta et al., 2006, Lin et al., 2007, Nguyen and Disteche, 2006). More recently, RNA-sequencing (RNA-seq) analyses showed that the X:A ratio in males and females is nearer 0.5, and therefore that X upregulation does not occur (Julien et al., 2012, Xiong et al., 2010). The discrepancy between these studies has been attributed to the choice of genes used to assay X upregulation. The X chromosome is enriched in tissue-specific genes, including those expressed in the testis and ovary (Deng et al., 2014, Khil et al., 2004, Mueller et al., 2008, Mueller et al., 2013). These genes are silent in the soma, and thus their inclusion can artificially lower estimations of the somatic X:A ratio (Deng et al., 2011). A reappraisal of X:A ratios using expression thresholds that exclude such genes has confirmed the existence of X upregulation (Deng et al., 2011, Lin et al., 2011, Yildirim et al., 2012), and mechanistic studies have identified transcriptional and post-transcriptional mechanisms by which upregulation is achieved (Deng et al., 2013, Faucillion and Larsson, 2015, Yildirim et al., 2012, Yin et al., 2009). X upregulation preferentially affects a subset of expressed X genes with dosage-sensitive housekeeping functions (Birchler, 2012, Pessia et al., 2012, Pessia et al., 2014).

To date, studies of X upregulation have focused on somatic tissues, and it is therefore unclear whether germ cells also conform to Ohno's hypothesis. In mice, primordial germ cells (PGCs) arise from the post-implantation epiblast and migrate along the hindgut endoderm before colonizing the gonad. During this time, they undergo genome-wide reprogramming in which the pluripotency gene network is reactivated, somatic genes are repressed, and genomic imprints are removed (Gkountela et al., 2015, Guo et al., 2015, Leitch et al., 2013, Seisenberger et al., 2012, Tang et al., 2015). In females, one of the two X chromosomes is already inactive prior to PGC specification (Hayashi et al., 2012, McMahon and Monk, 1983, Sugimoto and Abe, 2007). During germline reprogramming, the inactive X chromosome is subsequently reactivated (Chuva de Sousa Lopes et al., 2008, de Napoles et al., 2007, Sugimoto and Abe, 2007). However, expression from the active X chromosome during and after reprogramming has not been examined, and therefore the status of X dosage compensation throughout male and female germline development is unclear.

To address this point, we have generated extensive RNA-seq datasets from wild-type XY male and XX female, as well as sex chromosomally abnormal XO female (Turner syndrome) and XX male (Klinefelter syndrome variant) mouse germ cells before, during, and after reprogramming. Consistent with Ohno's hypothesis, early male and female germ cells exhibit upregulation of the active X chromosome. Later, however, they display unusual and sexually dimorphic dosage compensation patterns. Female germ cells exhibit a phase of X dosage excess, during which X:A ratios exceed 1, while male germ cells, conversely, exhibit X dosage decompensation, with X:A ratios falling below 1. These X dosage compensation patterns are conserved in human germ cells. Intriguingly, sex chromosome variant mice manifest a “sex-reversed” dosage compensation state: XO female germ cells become dosage decompensated like XY males, while XX male germ cells exhibit X dosage excess like XX females. Our studies reveal important differences in X dosage compensation states between the germline and soma and provide fresh insight into the etiology of subfertility caused by sex chromosome abnormalities.

## Results

### Creation of Germ Cell and Somatic Cell RNA-Seq Datasets for Analysis of X Dosage Compensation

For our analysis, we wished to track X dosage compensation states throughout the entirety of embryonic germ cell development (Figure S1). In mice, PGCs arise from the post-implantation epiblast at embryonic day 7.25 (E7.25) (Saitou, 2009). These PGCs migrate along the hindgut endoderm and colonize the gonad between E10.5 and E11.5 (Saitou, 2009). Following sex determination at E11.5, germ cells develop in a sexually dimorphic manner. In females, they divide mitotically until E12.5, before entering meiosis. In males, in contrast, germ cells divide mitotically until E16.5, when they undergo quiescence at the G_0_ phase of the cell cycle (Hilscher, 1974, Vergouwen et al., 1991). After birth, they resume mitosis and enter meiosis at around postnatal day 10 (P10).

As a baseline reflecting the epigenetic state of PGC and somatic cell precursors, we isolated E6.5 epiblasts (Seisenberger et al., 2012). For later time points, we used mice carrying a previously described *Oct4*-EGFP transgene (Yoshimizu et al., 1999). We used fluorescence-activated cell sorting (FACS) to collect highly purified populations of germ cells (EGFP-positive) and gonadal somatic cells (EGFP-negative) from both sexes at E9.5, E11.5, E12.5, E14.5, E15.5, E16.5, and E18.5, as well as purified spermatogonia at P2. For purification of leptotene/zygotene spermatocytes at P11, we used an alternative reporter, ROSA26-Tomato-EGFP (Muzumdar et al., 2007) carrying a *Stra8*-Cre transgene (Sadate-Ngatchou et al., 2008). Non-gonadal somatic cell control datasets were generated from E14.5 male and female liver and tail. Germ cells from individual embryos were processed to make cDNA libraries and served as biological replicates. We generated 184 libraries for our analysis from a total of 60 separate conditions (Table S1). To assess replicate correlation, we performed t-distributed stochastic neighbor embedding (t-SNE) analysis on our dataset, which revealed a high degree of cross-replicate clustering (Figure S2A). We also computed Spearman correlation coefficients between samples, which were typically higher than 0.8, confirming a high level of replicate correlation (Figure S2B).

**Figure 1:**
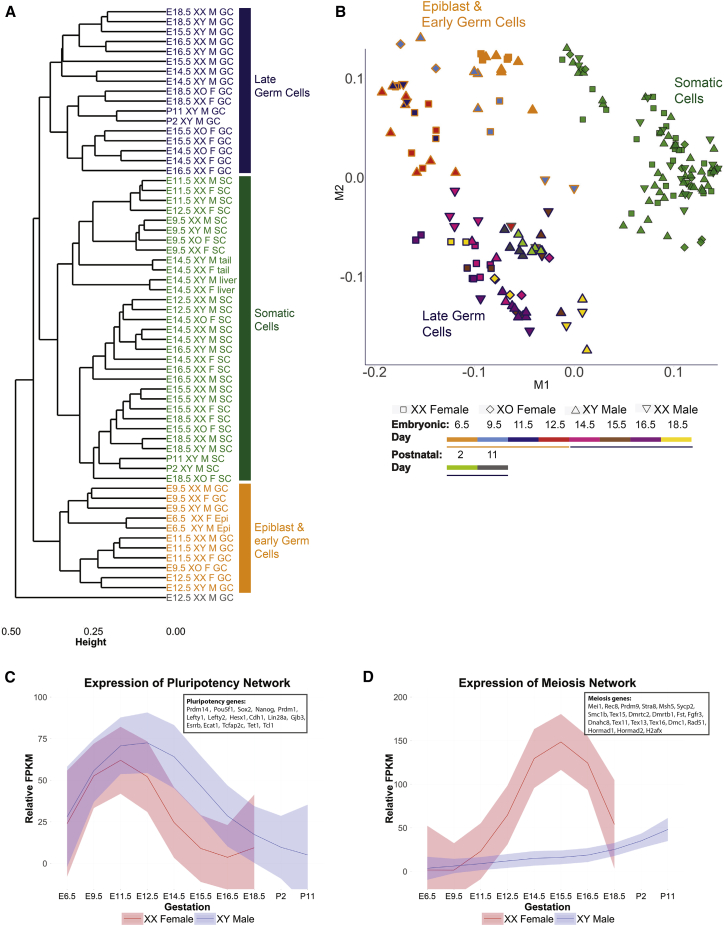
Transcriptome Profiling of Mouse Germ Cells (A) Unsupervised hierarchical clustering of all dataset samples. The dendrogram shown is based on Jensen-Shannon distances between conditions. Dark orange, epiblast and early germ cells; dark blue, late germ cells; green, somatic cells; gray, E12.5 XX male germ cells. (B) MDS plot of gene expression in all replicates within the dataset. (C) Time course of relative expression (FPKM) for pluripotency genes in XY males and XX females. (D) Time course of relative expression (FPKM) for meiosis genes in XY males and XX females.

Unsupervised hierarchical clustering of RNA-seq profiles revealed that early germ cells, late germ cells, and somatic (gonadal and non-gonadal) cells formed three distinct branches (59 of our 60 conditions), suggesting that our transcriptomic data recapitulated the ontology of germ cell development (Figure 1A). We further interrogated the data using multi-dimensional scaling (MDS), which once again highlighted the transcriptional distinction between these three groups. The first dimension separated somatic cells from germ cells, and developmental progression was noted along the second dimension, indicating progressive germ cell differentiation after PGC colonization of the gonad (Figure 1B). The single outlier condition in hierarchical clustering (E12.5 XX male) clearly segregated as germ cells during MDS. We assayed the expression of previously described pluripotency and meiotic genes (Seisenberger et al., 2012) in our germ cell populations. In both male and female germ cells, pluripotency genes were upregulated following the time of PGC specification at E7.25, and were subsequently repressed following sex determination (Figure 1C). Meiotic genes were upregulated in female germ cells from E12.5 but remained repressed at this time point in males (Figure 1D). Thus, our germ cell population faithfully recapitulated the orderly program of sex-specific changes that occur during germ cell development.

### Upregulation of the Active X Chromosome in Non-gonadal and Gonadal Somatic Cells

Next, we ascertained whether X upregulation occurs in somatic cells. We analyzed X chromosome activity in XX and XY non-gonadal somatic cells (E14.5 liver and tail) and gonadal somatic cells (E9.5–E18.5). In both males and females, these cells carry one active X chromosome, as females undergo somatic X chromosome inactivation. In order to assay expression at a chromosome-wide level, we charted median X chromosome expression in relation to that of median expression from the autosomes as a comparison. This X:A ratio was calculated as the ratio of the respective medians, with 95% confidence intervals of the ratio computed using the bootstrap method, which involves random sampling from a distribution with replacement (Efron and Tibshirani, 1993).

**Figure 2:**
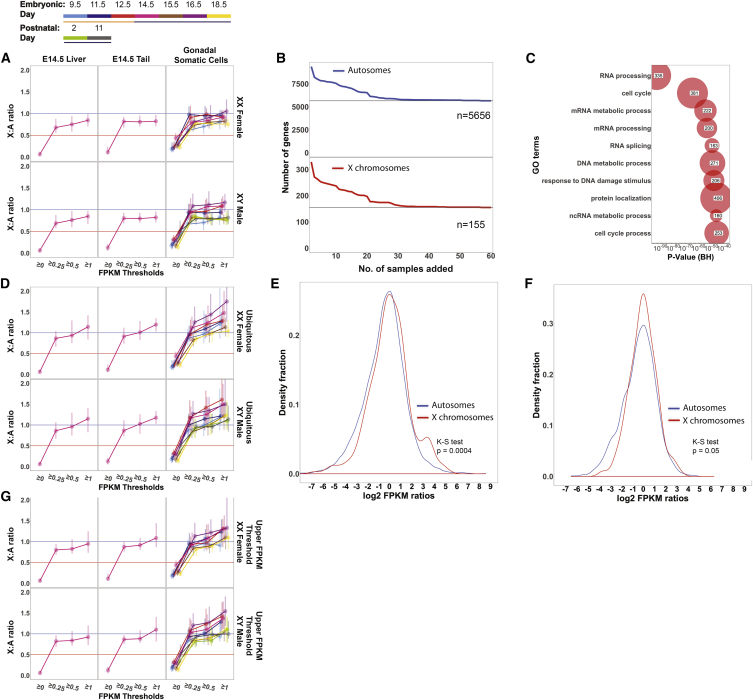
Analysis of X:A Ratios in Somatic Cells (A) Bootstrapped X:A ratios from E14.5 liver and tail and gonadal somatic cell populations (E9.5–P11; E9.5 refers to caudal embryo somatic cells) using different lower FPKM thresholds, and focusing on all genes (i.e., “non-ubiquitous genes”). (B) Definition of a ubiquitous gene set by addition of sequential samples of germ cells and somatic cells. The number of genes expressed in “all samples” is predictably high when the number of samples included is low and decreases as more samples are added before plateauing at a stable gene set. (C) Gene ontology (GO) enrichment analysis, defined using Database for Annotation, Visualization and Integrated Discovery (DAVID), of ubiquitously expressed genes (5,656 on the autosomes and 155 on the X chromosome). Benjamini-Hochberg-corrected p values were plotted against the top ten “biological processes” GO terms. (D) X:A ratios for the same samples as in (A), but considering only ubiquitous genes. (E) Density plot of log_2_ FPKM ratios of expression in E14.5 XY male gonadal somatic cells and E14.5 XY male liver for the X chromosome versus the autosomes. (F) Density plot of log_2_ FPKM ratios of expression in E14.5 XY male gonadal somatic cells and E14.5 XY male liver for the X chromosome versus the autosomes, after imposition of an upper FPKM expression threshold. (G) X:A ratios for the same samples as in (A) and (D) considering only ubiquitous genes together with an upper FPKM threshold.

Consistent with earlier studies (Julien et al., 2012, Xiong et al., 2010), when all genes with a fragments per kilobase of transcript per million reads (FPKM) ≥0 were included in our analysis, X:A ratios were low in non-gonadal and gonadal somatic tissues, implying that X upregulation does not occur (Figure 2A). However, the X chromosome is enriched relative to the autosomes in genes that are silent in somatic cells, and therefore including these genes artificially lowers X:A ratios (Deng et al., 2011, Yildirim et al., 2012). When we subsequently implemented increasing thresholds of expression ranging from FPKM 0.25 to 1, X:A ratios increased in both non-gonadal and gonadal somatic cells (Figure 2A). Hereafter, we implement an FPKM of ≥1, which, based on other studies (Deng et al., 2011, Lin et al., 2011, Yildirim et al., 2012), is appropriate for assaying X upregulation (see Table S2 for numbers of genes exhibiting FPKM ≥1 for each sample).

While our data supported Ohno's prediction that expression from the single active X chromosome is upregulated in male and female somatic tissues (Ohno, 1967), two points warranted attention. Firstly, contrary to expectations, confidence intervals for X:A ratios in many of our somatic samples did not cross 1 when an FPKM of ≥1 was used (Figure 2A). Upregulation affects a subset of X chromosome genes with dosage-sensitive housekeeping functions (Birchler, 2012). Inclusion of expressed but non-dosage-compensated X genes dilutes X:A ratios to less than 1 (Pessia et al., 2012). We therefore refined our approach, employing a strategy (Ramskold et al., 2009) in which autosomal and X chromosome genes with housekeeping functions were identified by virtue of being ubiquitously expressed, i.e., exhibiting an FPKM ≥1, throughout our entire RNA-seq sample dataset. To identify this gene set, we added samples in a stepwise manner, on each occasion retaining only genes that were expressed in all conditions. As expected with this approach, addition of successive samples initially resulted in a drop in the number of genes. However, the number subsequently stabilized at 5,656 autosomal and 155 X chromosomal genes (Figure 2B; Table S3). This ubiquitous gene set represented a sizable proportion of all expressed genes (FPKM ≥1), for example, 49.7% of autosomal and 41.2% of X-linked genes in E14.5 male liver. The number of ubiquitous genes did not change when liver, which expresses a lower number of genes compared with other tissues (Ramskold et al., 2009), was excluded. Gene ontology term enrichment analysis provided strong evidence that these genes had essential housekeeping roles, for example, in the cell cycle, RNA processing, and protein localization (Figure 2C). When we repeated our analysis focusing on these ubiquitous genes, X:A ratio confidence intervals in somatic tissues crossed 1 (Figure 2D). Our approach therefore successfully enriched for X genes that are subject to upregulation.

A second consideration was that the X chromosome is over-represented in genes expressed in reproductive tissues, including gonadal somatic cells and germ cells (Deng et al., 2011, Khil et al., 2004, Mueller et al., 2008, Mueller et al., 2013, Wang et al., 2001). Such genes have tissue-biased functions, and are therefore less likely to require dosage compensation. The inclusion of such highly expressed genes can skew X:A ratios to higher levels (Deng et al., 2011). Indeed, we noted that X:A ratios were higher in some of our gonadal somatic populations than in non-gonadal somatic ones (Figures 2A and 2D). To investigate this difference further, we conducted a pairwise analysis of gene expression ratios comparing E14.5 XY male gonadal somatic cells to E14.5 XY male liver (Figure 2E). There was a significant difference in the distribution of expression from the X chromosome compared with the autosomes, and a greater proportion of highly expressed genes on the X chromosome were expressed in gonadal somatic cells (Kolmogorov-Smirnov test: p = 0.0004; Figure 2E). We controlled for this effect by imposing an upper FPKM threshold set to exclude the top centile of gene expression (Figure 2F; see STAR Methods for details). A total of 4,188 autosomal and 102 X-linked loci remained from the original ubiquitous gene set upon imposing this threshold (Table S4). As expected, when we included this threshold, X:A ratios in gonadal somatic cell populations fell, now resembling those seen in non-gonadal somatic populations (Figure 2G). In light of these findings, we decided to analyze X dosage compensation patterns during germline development both in the presence and absence of an upper FPKM threshold. Finally, we applied our criteria to existing RNA-seq datasets from embryonic stem cells (ESCs) and epiblast-like cells (Hayashi et al., 2011, Lowe et al., 2015, von Meyenn et al., 2016). We observed X:A ratios crossing 1 in male ESCs and epiblast-like cells, and crossing 2 in female ESCs (Figure S3).

### XX Female Germ Cells Exhibit Excess X Chromosome Dosage During Reprogramming

Next, we analyzed X dosage compensation patterns during and after reprogramming in the XX female germline. Previous work has shown that one of the two X chromosomes is already inactive in the epiblast prior to PGC specification (Hayashi et al., 2012, McMahon and Monk, 1983, Sugimoto and Abe, 2007), and subsequently reactivates during germline reprogramming (Chuva de Sousa Lopes et al., 2008, de Napoles et al., 2007, Sugimoto and Abe, 2007). However, the behavior of the active X chromosome during this period has not been examined.

**Figure 3:**
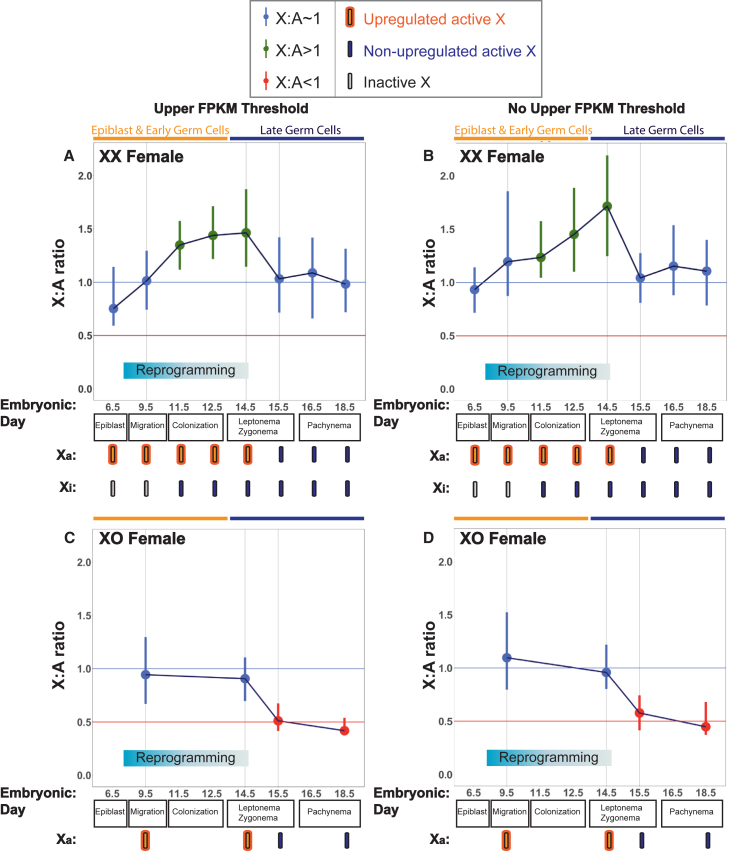
X:A Ratios in XX and XO Female Mouse Germ Cells (A) X:A ratios of ubiquitously expressed genes in XX females using an FPKM ≥1 and an upper expression threshold. (B) X:A ratios of ubiquitously expressed genes in XX females using an FPKM ≥1 and no upper expression threshold. (C) X:A ratios of ubiquitously expressed genes in XO females using an FPKM ≥1 and an upper expression threshold. (D) X:A ratios of ubiquitously expressed genes in XO females using an FPKM ≥1 and no upper expression threshold. Dark orange, epiblast and early germ cells; dark blue, late germ cells.

X:A ratios approximated 1 in epiblast cells at E6.5, whether or not an upper FPKM threshold was used (Figures 3A and 3B). Given that, in these cells, one of the two X chromosomes is silenced, this finding shows that the active X chromosome is already upregulated prior to PGC specification (see next section for confirmation). X dosage compensation was thereafter retained during germ cell migration at E9.5 (Figures 3A and 3B). Interestingly, however, from E11.5, X:A ratios increased, exceeding 1, resulting in a state of X dosage excess (Figures 3A and 3B). The relative overexpression of X genes persisted for 3 days until E14.5, i.e., after gonadal colonization and entry into meiosis (Figures 3A and 3B). This period is noteworthy since it corresponds to the time during which genes on the inactive X chromosome gradually reactivate (Chuva de Sousa Lopes et al., 2008, de Napoles et al., 2007, Sugimoto and Abe, 2007). We reappraised the behavior of the inactive X chromosome from E6.5 to E14.5 using *Xist* RNA fluorescence in situ hybridization (FISH) (Figure S4), and found that the major loss of *Xist* clouds occurred between E9.5 and E11.5, precisely coincident with the shift to X dosage excess as determined by X:A ratio calculations (Figure S4). Subsequently, X dosage compensation was reinstated, with X:A ratios returning to 1 at E15.5 and remaining so thereafter (Figures 3A and 3B). Thus, XX female germ cells show X dosage compensation states that are dynamic and that differ from the soma.

### XO Female Germ Cells Exhibit X Dosage Decompensation Following Reprogramming

Our findings demonstrated that during germline reprogramming in XX female germ cells, expression of X genes undergoes dynamic changes relative to those of the autosomes, resulting in a period of excess X chromosome dosage. However, we could not decipher the relative contribution of the two X chromosomes to this unusual X dosage compensation state. To better understand this phenomenon, we repeated our analysis using germ cells from Turner syndrome female (XO) mice, which carry one rather than two X chromosomes (Figures 3C and 3D). XO mice are viable (Burgoyne et al., 1983a, Burgoyne et al., 1983b), and therefore presumably achieve X dosage compensation through upregulation of their single active X chromosome. By assaying the behavior of this single active X chromosome in isolation, the cause of the X dosage excess observed in XX females can subsequently be deduced. We focused on four time points that in XX females represented states of initial X dosage compensation (E9.5), X dosage excess (E14.5), and reinstatement of X dosage balance (E15.5 and E18.5; Figures 3A and 3B). XO females are subfertile, but the timing of germ cell loss in these mice initiates later, at E19.5 (Burgoyne and Baker, 1981, Burgoyne and Baker, 1985). Thus, our data were not confounded by germ cell elimination.

As observed in XX germ cells (Figures 3A and 3B), X:A ratios in XO germ cells at E9.5 approximated 1, confirming that XO PGCs achieve dosage compensation through upregulation of the single active X chromosome (Figures 3C and 3D). Notably, however, at E14.5, when X dosage excess was observed in XX germ cells (Figures 3A and 3B), XO germ cells retained X dosage balance (Figures 3C and 3D). A pairwise comparison revealed that the distribution of X chromosome expression was significantly different between XO and XX females at this age (Wilcoxon test, p = 8 × 10^−4^). Thus, in the female germline, upregulation of the active X chromosome is maintained at E14.5. By deduction, the state of X dosage excess in XX females at E14.5 (Figures 3A and 3B) results from the additive effects of persistent upregulation of the active X chromosome, and reactivation of the previously inactive X chromosome.

Interestingly, dosage compensation patterns in XO female germ cells changed markedly between E14.5 and E15.5. At E15.5, when XX germ cells regained X:A balance, XO germ cells became X dosage decompensated, i.e., the X:A ratio fell below 1 (Figures 3C and 3D). Thus, upregulation of the active X chromosome is lost during a 24 hr period in early meiosis. Subsequently, at E18.5, X:A ratios decreased further (Figures 3C and 3D). At this stage, XO germ cells have entered pachynema, when meiotic silencing begins to inactivate genes on the single unsynapsed X chromosome (Baarends et al., 2005, Turner et al., 2005). Meiotic silencing affects only 50% of XO oocytes and occurs in an inefficient manner (Cloutier et al., 2015a, Cloutier et al., 2015b), potentially explaining why X:A ratios did not decrease more dramatically. We conclude that germline reprogramming in females is associated not only with reactivation of the inactive X chromosome but also with loss of upregulation of the active X chromosome, and that the completion of these events by E15.5 permits reinstatement of X dosage balance in E15.5 XX female germ cells. In addition, XX and XO females exhibit markedly different dosage compensation patterns.

### XY Male Germ Cells Exhibit X Dosage Decompensation Following Reprogramming

Our findings showed that in the female germline, upregulation of the active X chromosome is lost following reprogramming, restoring X:A balance in XX females but causing X dosage decompensation in XO females. XY males, like XO females, have a single X chromosome. We therefore asked whether X dosage decompensation was also observed in the male germline. To address this, we tracked dosage compensation patterns throughout male germ cell development at equivalent time points to those analyzed in XX females.

**Figure 4:**
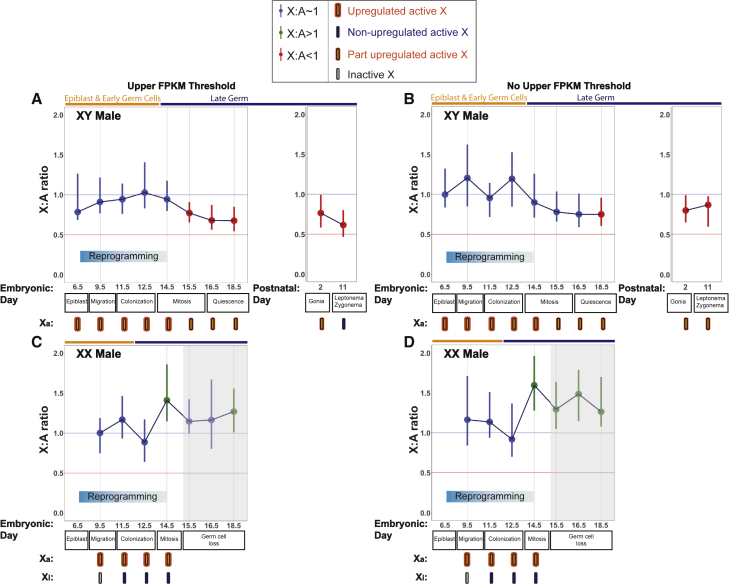
X:A Ratios in XY and XX *Sry* Male Mouse Germ Cells (A) X:A ratios of ubiquitously expressed genes in XY males using an FPKM ≥1 and an upper expression threshold. (B) X:A ratios of ubiquitously expressed genes in XY males using an FPKM ≥1 and no upper expression threshold. (C) X:A ratios of ubiquitously expressed genes in XX *Sry* males using an FPKM ≥1 and an upper expression threshold. (D) X:A ratios of ubiquitously expressed genes in XX *Sry* males using an FPKM ≥1 and no upper expression threshold. Dark orange, epiblast and early germ cells; dark blue, late germ cells.

As noted in XX and XO females, XY epiblast cells exhibited X dosage compensation at E6.5, and X:A ratios subsequently remained at 1 from E9.5 and E14.5 (Figures 4A and 4B). However, at later time points, X:A ratios in XY male germ cells fell, with upper confidence intervals no longer crossing 1. This phenomenon was observed from E15.5 when an upper FPKM threshold was imposed and from E18.5 when no upper threshold was imposed. In both scenarios, the X dosage decompensated state persisted postnatally in spermatogonia and even in leptotene/zygotene spermatocytes. At E15.5, the distribution of X chromosome expression differed significantly between XY males and XO females (Wilcoxon test, p = 0.0041), suggesting that the extent of decompensation in XY male germ cells was not as pronounced as in XO females. However, at E15.5, male germ cells are in mitosis, while female germ cells are in leptonema/zygonema. When we compared stage-matched XY male (P11) and XO female (E15.5) leptotene/zygotene cells, the X chromosome expression distributions did not differ (Wilcoxon test, p = 0.2362). We conclude that XY males exhibit X dosage decompensation after reprogramming, and that XO females exhibit a “sex-reversed” X dosage compensation pattern, i.e., one that is reminiscent of XY males rather than XX females.

### XX Male Germ Cells Exhibit Excess X Chromosome Dosage During Reprogramming

Our analysis demonstrated that X dosage compensation patterns in XO females resemble those in XY males. We next asked the converse question, namely whether dosage compensation patterns in XX males are similar to those in XX females. We calculated X:A ratios in germ cells derived from Klinefelter syndrome variant (XX) males. These mice have two X chromosomes, and thus undergo X chromosome inactivation, but they are male due to presence of a sex reversing *Sry* transgene (Mahadevaiah et al., 1998). XX males exhibited dosage balance at E9.5, E11.5 and E12.5 (Figures 4C and 4D). However, as observed in XX females (Figures 3A and 3B), at E14.5 X:A ratios in XX males exceeded 1 (Figures 4C and 4D), and the distribution of X chromosome expression at this stage differed significantly from that in XY males (Wilcoxon test: p = 9 × 10^−4^). Germ cell loss is first evident in Klinefelter male mice from E15.5 (Hunt et al., 1998); we therefore avoided drawing conclusions about X:A ratios at this stage and after. Our findings show that, like XX females, XX males exhibit excess X chromosome dosage during reprogramming.

### Dosage Decompensation in XY Male Germ Cells is not Corrected by Expression of Autosomal Retrogenes

A number of X genes, including some within our dataset, e.g., *Pgk1*, *Pdha1*, have duplicate copies known as retrogenes. These arise by reverse transcription of X-derived RNAs and subsequent integration at autosomal sites, and thus differ from their parental copies in being intronless (Kaessmann et al., 2009, Khil et al., 2005, Wang, 2004). Retrogenes show testis-biased expression, and some encode proteins with similar functions to their X-linked progenitors (Bradley et al., 2004, Danshina et al., 2010, Emerson et al., 2004, Rohozinski and Bishop, 2004, Wang and Page, 2002). We wondered if X-derived retrogenes were expressed after germline reprogramming and, if so, whether their expression could compensate for the drop in X chromosome output that we observed in XY males during this period. Using the criteria that they should be intronless, putatively protein coding, autosomally encoded, and share >80% nucleotide identity with their X-parental copies, we identified retrogenes for 14 of our 155 ubiquitous X genes (Figure S5A). These retrogenes were expressed at very low levels relative to their X counterparts from E6.5–P11 (Figure S5A). This finding was consistent with previous work showing that X-derived retrogenes initiate expression later, during, or after pachynema (Kaessmann et al., 2009, Khil et al., 2005, Wang, 2004). The X dosage decompensated state in XY male germ cells was not altered when we combined retrogene-derived RNA-seq FPKMs with those from their parental X-encoded progenitors at each stage of germ cell (Figure S5B). We conclude that retrogenes do not compensate for the decrease in expression of their X-linked progenitors.

### Conserved Dosage Compensation Patterns During Human Germline Development

**Figure 5:**
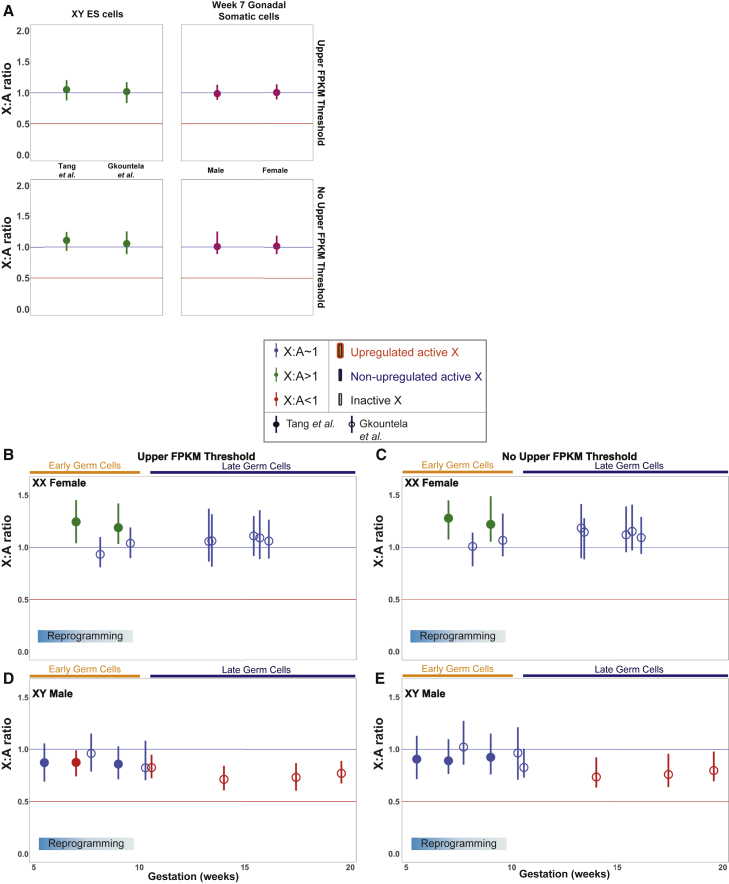
X:A Ratios in Human XY ESCs, Gonadal Somatic Cells, and Germ Cells (A) X:A ratios of ubiquitously expressed genes in XY ESCs, and week 7 male gonadal somatic cells and female gonadal somatic cells using an FPKM ≥1 and the presence or absence of an upper FPKM threshold. (B) X:A ratios of ubiquitously expressed genes in XX female germ cells using an FPKM ≥1 and an upper expression threshold. Data from Tang et al. (2015) are shown with closed circles; data from Gkountela et al. (2015) are shown with open circles. (C) X:A ratios of ubiquitously expressed genes in XX female germ cells using an FPKM ≥1 and no upper expression threshold. (D) X:A ratios of ubiquitously expressed genes in XY male germ cells using an FPKM ≥1 and an upper expression threshold. (E) X:A ratios of ubiquitously expressed genes in XY male germ cells using an FPKM ≥1 and no upper expression threshold. Dark orange, early germ cells; dark blue, late germ cells.

We next turned our attention to X chromosome activity during human germline reprogramming. We analyzed existing RNA-seq datasets that comprised human gonadal somatic cells and XY ESCs, as well as germ cells purified using FACS for c-KIT alone (Gkountela et al., 2015) or both c-KIT and TNAP (Tang et al., 2015). The data encompassed a broad timeline of human PGC development including early germ cells, when germline reprogramming occurs (weeks 5.5, 7, and 9) and later germ cells, when sexually dimorphic development takes place (Figure S1). Where available, biological replicates were pooled as indicated (Tang et al., 2015). We used this entire sample set to generate a set of ubiquitously expressed (FPKM ≥1) genes, in a manner similar to our approach in mice. We identified 8,226 autosomal and 259 X-linked ubiquitously expressed genes (Table S5), which fell to 7,719 autosomal and 236 X-linked genes when an upper FPKM threshold was imposed (Table S6). We observed X:A ratios of 1 for these genes in XY ESCs and gonadal somatic cells, in the presence or absence of an upper FPKM threshold (Figure 5A), indicating that X upregulation occurs in these populations. Notably, however, germ cells exhibited dosage compensation patterns that differed from the soma, and were reminiscent of those seen in mouse germ cells. During germline reprogramming (weeks 7–9), when reactivation of the inactive X chromosome takes place (Tang et al., 2015), XX female samples derived from Tang et al. (2015) exhibited X dosage excess, both in the presence or absence of an upper FPKM threshold (Figures 5B and 5C). This phenomenon was not observed in males (Figures 5D and 5E). Furthermore, after germline reprogramming, while X:A ratios in females returned to 1 (Figures 5B and 5C), those in males fell below 1, resulting in an X dosage decompensated state (Figures 5D and 5E). We conclude that differences in X dosage compensation states exhibited between somatic and germ cells, and between females and males, are conserved in mice and humans.

## Discussion

**Figure 6:**
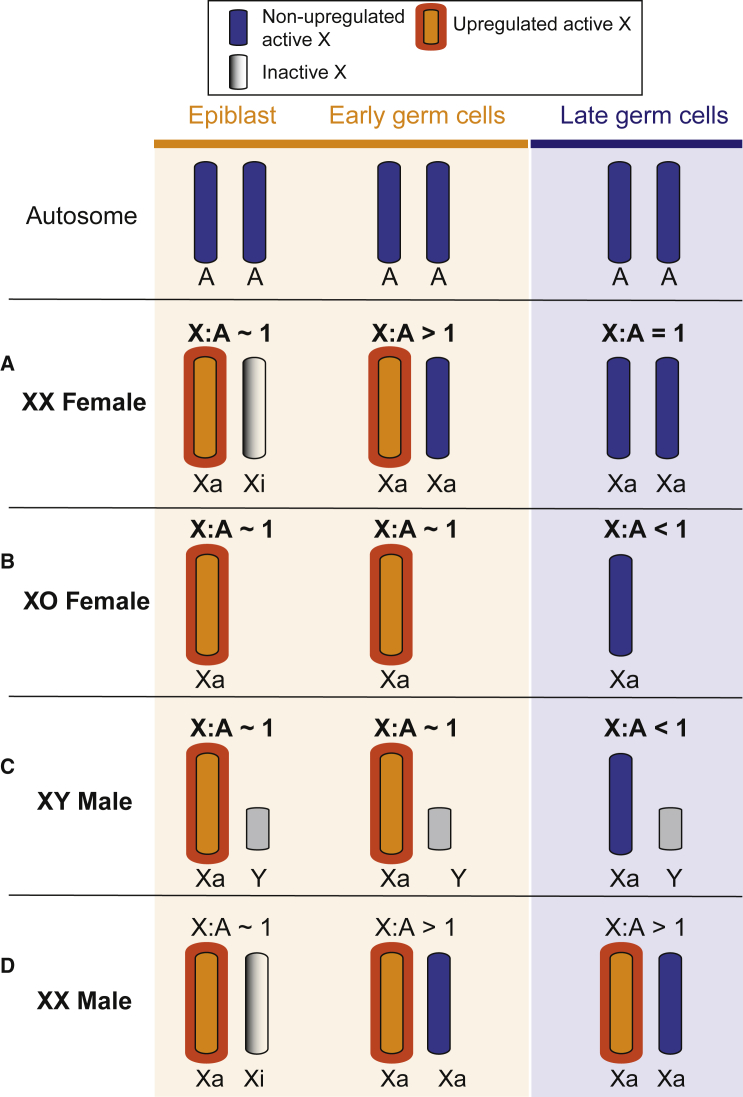
A Model for X Dosage Compensation Patterns A model for X dosage compensation patterns in the XX Female (A), XO female (B), XY male (C), and XX male (D) mammalian germline. Prior to reprogramming, XX female and XX male germ cells have one upregulated X chromosome (Xa, orange) and one inactive X chromosome (Xi, gray), while XY males and XO female cells have a single upregulated X chromosome (Xa, orange). In all four cases, the dosage of X genes is balanced with that of the autosomes (A, blue), i.e., the X:A ratio is 1. During reprogramming, upregulation of the active X chromosome is maintained in all four genotypes, but in XX females and XX males, the inactive X chromosome begins to reactivate (gray becomes blue). The outcome is X:A ratios greater than 1 in XX females and in XX males. Later, X upregulation is lost, reinstating X:A ratios in XX females to 1, but leaving XY males and XO females X dosage decompensated, with X:A ratios less than 1. XX males show germ cell loss from E15.5, which may be due to high X:A ratios seen.

Here, we have tracked X dosage compensation patterns in mouse and human germ cells from their inception through to their later development. We uncover unusual X dosage compensation states that differ between the germline and the soma and between males and females, and that are conserved in humans (Figure 6).

During their specification and migration, female and male germ cells exhibit X:A ratios of 1, showing that upregulation of the active X chromosome is present in both sexes during early germline development (Figures 6A–6D). Subsequently, however, X dosage compensation patterns become sexually dimorphic. During reprogramming in XX female mice, X:A ratios exceed 1, generating a state of X dosage excess that persists for 3 days from E12.5 to E14.5 (Figure 6A). We propose that the excess X chromosome output arises from reactivation of the inactive X chromosome, combined with ongoing upregulation of the active X chromosome. While supported by analysis of XO females, it is also possible that this excess X dosage in XX females results from a modest increase in expression from both X chromosomes. This alternative hypothesis could be tested in future by allele-specific RNA-seq. Upregulation of the active X chromosome is subsequently reversed between E14.5 and E15.5. In XX females, this corrects the hyperactive X state (Figure 6A), but in XO females it leads to X dosage decompensation (Figure 6B). The decompensated state in XO females is independent of meiotic silencing, which occurs later, at E16.5–E18.5 (Cloutier et al., 2015b). Our findings, together with previous work (Fukuda et al., 2015), show that X upregulation no longer occurs in XO and XX females after reprogramming.

As observed in female germ cells, output from the active X chromosome in XY male germ cells falls following reprogramming, resulting in X:A ratios below 1 (Figure 6C). At leptonema/zygonema, the extent of decompensation in XY males is similar to that of XO females. Nevertheless, reversal of X upregulation in XY males clearly occurs over a more protracted period (i.e., days) than in XO females (hours). Sexual dimorphisms are regulated by X dosage or X imprinting effects, or by the presence or absence of the Y chromosome (Arnold et al., 2012). Given that XO females and XY males both have a single X chromosome of maternal origin, differences between these models must be due to the Y chromosome. The Y chromosome could influence X dosage compensation states as a result of *Sry* determining male gonad development (i.e., phenotypic sex), or/and the continued effects of other Y-encoded genes that could modulate X dosage compensation states. It is already well established that the X chromosome is silenced during pachynema by the process of meiotic sex chromosome inactivation (MSCI) (Ichijima et al., 2012, Inagaki et al., 2010, Turner, 2007, Yan and McCarrey, 2009), but its activity before this time has not been thoroughly investigated. Our current findings show that expression from the X chromosome in fact undergoes a stepwise decrease during male germline development: prior to reprogramming, the X chromosome is fully upregulated, at leptonema/zygonema upregulation is lost, and subsequently, during pachynema, it is fully silenced by MSCI.

Finally, our studies are informative with respect to understanding the etiology of infertility in sex chromosome aneuploidies. XO females exhibit X chromosome decompensation reminiscent of that seen in wild-type males, while XX males exhibit X dosage excess like that in wild-type females. We suggest that this sex-reversed X dosage compensation pattern could contribute to the infertility phenotypes. For example, while it is accepted that infertility in XX males is due to reactivation of the inactive X chromosome (Hall et al., 2006, Mroz et al., 1999), one model attributes this specifically to a double dose of genes showing spermatogonial-specific expression (De Jonge and Barratt, 2006). Our data present an additional possibility, in which germ cell loss in XX males results from a mismatch between germline sex and X dosage compensation state. Previous work advocates a predominant role for dosage-sensitive X genes in sex chromosome aneuploid phenotypes (Pessia et al., 2012). In either scenario, preventing X chromosome reactivation or promoting loss of the excess X chromosome may prove of therapeutic benefit in Klinefelter syndrome infertility.

## STAR★Methods

### Key Resources Table

**Table:** 

Reagent or Resource	Source	Identifier
**Critical Commercial Assays**		

Ultralow input RNA-Seq Kit (SMARTER)	Clontech	Cat No #634935

**Deposited Data**		

Our raw and analyzed RNA-seq dataset	This paper	European Nucleotide Archive (EMBL-EBI) E-MTAB-4616

**Experimental Models: Organisms/Strains**		

Mouse: Oct-4 EGFP	Wolf Reik/Azim Surani, Cambridge, UK	-na-
Mouse: ROSA26- 540 Tomato-EGFP strain (ROSAmT/mG)	The Jackson Laboratory	Gt(ROSA)26Sortm4(ACTB-tdTomato,-EGFP)Luo
Mouse: XY- +Sry (B6)	Francis Crick Institute	-na-
Mouse: X^Y^O male	Francis Crick Institute, Burgoyne, 1998	-na

**Sequence-Based Reagents**		

Xist RNA FISH probes	Mahadevaiah et al., 2009	fosmid probe: W11-2363-H9

**Software and Algorithms**		

TopHat v2.0.13	Genome Biology 2013 14:R36	http://www.ccb.jhu.edu/software/tophat/index.shtml
Cufflinks v2.2.1	Trapnell et al., 2012	http://cole-trapnell-lab.github.io/cufflinks/
R v.3.2.0	R Core Team (2016)	https://www.R-project.org/
cummeRbund v2.7.2 (R package)	L. Goff, C. Trapnell, D. Kelley	http://compbio.mit.edu/cummeRbund/
pairwiseCI (R package)	Frank Schaarschmidt [aut, cre], Daniel Gerhard [aut]	https://cran.r-project.org/
Rtsne	Jesse Krijthe [aut, cre], Laurens van der Maaten [cph] (Author of original C++ code)	https://cran.r-project.org/

**Other**		

Smart-Seq2 protocol	Picelli et al., 2014	NA
Mouse genome and annotation	ftp://igenome:G3nom3s4u@ussd-ftp.illumina.com/Mus_musculus/UCSC/mm10/Mus_musculus_UCSC_mm10.tar.gz	mm10
Publically available data ES-cell RNA-seq datasets accessed	European Nucleotide Archive (EMBL-EBI)	SRR1448385, SRR1448386, SRR1448387, SRR1448388, SRR4241903, SRR4241904, SRR4241905, SRR4241906, SRR4241907, SRR4241908

### Contact for Reagent and Resource Sharing

Further information and requests for reagents may be directed to, and will be fulfilled by the Lead Contact, Dr. James Turner, james.turner@crick.ac.uk.

### Experimental Model Subject Details

#### Mice

Oct4-EGFP mice were obtained from the Reik lab (Babraham, Cambridge, UK) and maintained on a B6 background. The reporter strain was used to isolate fluorescently marked germ cells through FACs. Homozygous XY^−^ +*Sry* (C57B6) Oct4-EGFP males were crossed with XX Oct4-EGFP females to yield XX female, XY male and XX male embryos used in the study.

XO embryos were generated by crossing an MF1 X^Y^O male (Burgoyne, 1998) to an XX Oct4-EGFP female. Timed matings were undertaken, and noon on the date of the vaginal plug was taken to be E0.5. Embryos were subsequently harvested on the stipulated day.

Leptotene/zygotene stage male germ cells were isolated at postnatal Day 11, by crossing a Stra8-*cre* line with ROSA26-Tomato-EGFP strain (ROSA^mT/mG^ strain; Mouse strain #007676 from The Jackson Laboratory), which enabled the timely isolation of these germ cells during the first-wave of male meiosis through FACS.

Samples were sexed as female (XX or XO) or male (XY or XX *Sry* transgenic) by a combination of gonadal inspection, conventional PCR genotyping and the presence of Y chromosome-derived transcripts from RNA-Seq data. Each condition comprised of at least two replicates. All animal procedures were in accordance with the United Kingdom Animal Scientific Procedures Act 1986 and were subject to local ethical review.

### Method Details

#### Isolation of Cells for RNA-Seq Analysis

Cell populations derived and purified from a single embryo at any stated age constituted a biological replicate to be used in subsequent steps. Germ cell and somatic populations were isolated at different stages, ranging from E9.5, E11.5, E12.5, E14.5, E15.5, E16.5, E18.5, P2, and P11. RNA was purified from individual E6.5 epiblasts.

#### Fluorescence-Activated Cell Sorting (FACS)

Germ cells (GFP-positive) and associated somatic cells (GFP-negative) were isolated separately from individual embryos using FACs sorting on the MoFLo XDP or FACS Aria platforms. Live cells, i.e. only those staining negative for propidium iodide, were collected, and typically purity checking on the GFP negative populations was >99%.

#### Isolation of RNA and Construction of cDNA Libraries

RNA was isolated from FACs sorted purified cell populations using the Ambion RNA isolation kit (Ambion #AM1931). Eluted RNA was used to obtain double-stranded-cDNA using the Clontech (SMARTER) Ultralow input RNA-Seq Kit according to the manufacturer's protocol, or the Smart-Seq2 protocol (Picelli et al., 2014). cDNA was normalized to 10 ng in low-TE buffer and was sheared using Covaris S-series, and was cleaned up with Zymo DNA conc-5 and eluted in 12 ul low-TE buffer. Libraries of ∼300 bp were generated using Nugen Ovation Ultralow DR (part No. 0330) with 15 PCR cycles. Paired-end sequencing of 50 cycles was performed on the Illumina HiSeq2500 sequencer in house.

#### RNA FISH

Sample embryos were collected and fixed in 4% paraformaldehyde overnight at 4°C. Samples were then placed in 30% sucrose solution overnight. These were then placed in Optimal Cutting Temperature (OCT) compound and transferred to appropriate molds, quick-frozen and then stored at −80°C until the time of cryosectioning. 5 μm cryosections were collected and placed on coverglasses, and subsequently processed with *Xist* RNA FISH probes using an established protocol (Mahadevaiah et al., 2009). Germ cells at E9.5, E11.5 and E14.5 were identified by the detection of expression of the *Oct4*-EGFP reporter transgene and nuclear morphology as visualised with DAPI DNA staining.

### Quantification, Statistical Analysis and Additional Resources

#### Bioinformatic Analyses

Reads were aligned to the mouse genome (mm10) using Tophat2 v2.0.13. Transcript abundances were calculated using Cufflinks2 and Cuffdiff (Kim et al., 2013, Trapnell et al., 2012, Trapnell et al., 2013). At a minimum we had included two biological replicates for each condition that was analyzed (see Table S1). As recommended, annotated rRNA, mitochondrial transcripts and other very highly abundant transcripts were ignored from the analysis. Calculated transcript abundances in FPKM (Fragments per Kilobase per Million reads) were subsequently used in further analyses in R. Data visualization, hierarchical clustering and MDS were performed using the cummeRbund package. X:A ratios with 95% confidence intervals were calculated using the pairwiseCI package in R using 'Median.ratio' with 1,000,000 bootstrap replications.

We imposed an upper FPKM threshold that corresponded to the lowest 99^th^ centile FPKM value of expression across conditions. ES Cell RNA seq datasets were accessed from the European Nucleotide Archive database.

Relevant samples from Study accession numbers PRJNA342888 and PRJNA253304 were accessed (Run accession numbers used are as stated in Figure S3) (Lowe et al., 2015, von Meyenn et al., 2016). These samples were aligned using Hisat2 –v2.0.5 to the mouse genome (mm10). Expression values (FPKM) and X:A ratios were calculated as stated.

#### Retrogene Analysis

Annotated retrogenes were retrieved from the UCSC Genome Bioinformatics database from the mm10 mouse genome using Table Browser function and 18,456 records were obtained. X-linked genes from our ubiquitously expressed set of genes (Table S2) were cross-referred to this dataset. 14 out of 155 ubiquitous X-genes had autosomal retrogene counterparts which were intronless, putatively protein-coding and shared >80% nucleotide identity with their X-parental copies.

### Data and Software Availability

#### Software

All software programs used in this study were from publicly available resources. Please refer to Key Resources Table for more details.

#### Data Resources

The dataset has been submitted via ArrayExpress to the European Nucleotide Archive (EMBL-EBI) and is publicly available under accession number ArrayExpress: E-MTAB-4616

## Author Contributions

Conceptualization: M.S. and J.T.; Methodology: M.S. and J.T.; Investigation: M.S., S.K.M., and J.T.; Validation: H.R., M.B.S., and A.H.F.M.P.; Writing: M.S. and J.T.; Reviewing and Editing: M.S. and J.T.; Funding Acquisition: J.T. and M.S.; Resources: O.O., L.B., and A.S.; Supervision: J.T.

## Supplemental Information

Document S1. Figures S1–S5 and Tables S1 and S2

Table S3. Ubiquitously Expressed Mouse Genes: No Upper FPKM Threshold, Related to Figures 2D, 3B, 3D, 4B, and 4D

Table S4. Ubiquitously Expressed Mouse Genes: Upper FPKM Threshold, Related to Figures 2G, 3A, 3C, 4A, and 4C

Table S5. Ubiquitously Expressed Human Genes: No Upper FPKM Threshold, Related to Figures 5C and 5E

Table S6. Ubiquitously Expressed Human Genes: Upper FPKM Threshold, Related to Figures 5B and 5D

Document S2. Article plus Supplemental Information

## References

[bib1] Adler D.A., Rugarli E.I., Lingenfelter P.A., Tsuchiya K., Poslinski D., Liggitt H.D., Chapman V.M., Elliott R.W., Ballabio A., Disteche C.M. (1997). Evidence of evolutionary up-regulation of the single active X chromosome in mammals based on Clc4 expression levels in *Mus spretus* and *Mus musculus*. Proc. Natl. Acad. Sci. USA.

[bib2] Arnold A.P., Chen X., Itoh Y. (2012). What a difference an X or Y makes: sex chromosomes, gene dose, and epigenetics in sexual differentiation. Handb. Exp. Pharmacol..

[bib3] Baarends W.M., Wassenaar E., van der Laan R., Hoogerbrugge J., Sleddens-Linkels E., Hoeijmakers J.H., de Boer P., Grootegoed J.A. (2005). Silencing of unpaired chromatin and histone H2A ubiquitination in mammalian meiosis. Mol. Cell Biol..

[bib4] Bachtrog D. (2013). Y-chromosome evolution: emerging insights into processes of Y-chromosome degeneration. Nat. Rev. Genet..

[bib5] Birchler J.A. (2012). Claims and counterclaims of X-chromosome compensation. Nat. Struct. Mol. Biol..

[bib6] Bradley J., Baltus A., Skaletsky H., Royce-Tolland M., Dewar K., Page D.C. (2004). An X-to-autosome retrogene is required for spermatogenesis in mice. Nat. Genet..

[bib7] Burgoyne P.S. (1998). The mammalian Y chromosome: a new perspective. Bioessays.

[bib8] Burgoyne P.S., Baker T.G. (1981). Oocyte depletion in XO mice and their XX sibs from 12 to 200 days post partum. J. Reprod. Fertil..

[bib9] Burgoyne P.S., Baker T.G. (1985). Perinatal oocyte loss in XO mice and its implications for the aetiology of gonadal dysgenesis in XO women. J. Reprod. Fertil..

[bib10] Burgoyne P.S., Evans E.P., Holland K. (1983). XO monosomy is associated with reduced birthweight and lowered weight gain in the mouse. J. Reprod. Fertil..

[bib11] Burgoyne P.S., Tam P.P., Evans E.P. (1983). Retarded development of XO conceptuses during early pregnancy in the mouse. J. Reprod. Fertil..

[bib12] Charlesworth B. (1996). The evolution of chromosomal sex determination and dosage compensation. Curr. Biol..

[bib13] Charlesworth B., Charlesworth D. (2000). The degeneration of Y chromosomes. Philos. Trans. R. Soc. Lond. B Biol. Sci..

[bib14] Chuva de Sousa Lopes S.M., Hayashi K., Shovlin T.C., Mifsud W., Surani M.A., McLaren A. (2008). X chromosome activity in mouse XX primordial germ cells. PLoS Genet..

[bib15] Cloutier J.M., Mahadevaiah S.K., ElInati E., Nussenzweig A., Toth A., Turner J.M. (2015). Histone H2AFX links meiotic chromosome asynapsis to prophase I oocyte loss in mammals. PLoS Genet..

[bib16] Cloutier J.M., Mahadevaiah S.K., ElInati E., Toth A., Turner J. (2015). Mammalian meiotic silencing exhibits sexually dimorphic features. Chromosoma.

[bib17] Conrad T., Akhtar A. (2011). Dosage compensation in *Drosophila melanogaster*: epigenetic fine-tuning of chromosome-wide transcription. Nat. Rev. Genet..

[bib18] Cortez D., Marin R., Toledo-Flores D., Froidevaux L., Liechti A., Waters P.D., Grutzner F., Kaessmann H. (2014). Origins and functional evolution of Y chromosomes across mammals. Nature.

[bib19] Danshina P.V., Geyer C.B., Dai Q., Goulding E.H., Willis W.D., Kitto G.B., McCarrey J.R., Eddy E.M., O'Brien D.A. (2010). Phosphoglycerate kinase 2 (PGK2) is essential for sperm function and male fertility in mice. Biol. Reprod..

[bib20] De Jonge C.J., Barratt C. (2006). The Sperm Cell: Production, Maturation, Fertilization, Regeneration.

[bib21] de Napoles M., Nesterova T., Brockdorff N. (2007). Early loss of Xist RNA expression and inactive X chromosome associated chromatin modification in developing primordial germ cells. PLoS One.

[bib22] Deng X., Hiatt J.B., Nguyen D.K., Ercan S., Sturgill D., Hillier L.W., Schlesinger F., Davis C.A., Reinke V.J., Gingeras T.R. (2011). Evidence for compensatory upregulation of expressed X-linked genes in mammals, *Caenorhabditis elegans* and *Drosophila melanogaster*. Nat. Genet..

[bib23] Deng X., Berletch J.B., Ma W., Nguyen D.K., Hiatt J.B., Noble W.S., Shendure J., Disteche C.M. (2013). Mammalian X upregulation is associated with enhanced transcription initiation, RNA half-life, and MOF-mediated H4K16 acetylation. Dev. Cell.

[bib24] Deng X., Berletch J.B., Nguyen D.K., Disteche C.M. (2014). X chromosome regulation: diverse patterns in development, tissues and disease. Nat. Rev. Genet..

[bib25] Dupont C., Gribnau J. (2013). Different flavors of X-chromosome inactivation in mammals. Curr. Opin. Cell Biol..

[bib26] Efron B., Tibshirani R. (1993). An Introduction to the Bootstrap.

[bib27] Emerson J.J., Kaessmann H., Betran E., Long M. (2004). Extensive gene traffic on the mammalian X chromosome. Science.

[bib28] Faucillion M.L., Larsson J. (2015). Increased expression of X-linked genes in mammals is associated with a higher stability of transcripts and an increased ribosome density. Genome Biol. Evol..

[bib29] Fukuda A., Tanino M., Matoba R., Umezawa A., Akutsu H. (2015). Imbalance between the expression dosages of X-chromosome and autosomal genes in mammalian oocytes. Sci. Rep..

[bib30] Gelbart M.E., Kuroda M.I. (2009). *Drosophila* dosage compensation: a complex voyage to the X chromosome. Development.

[bib31] Gendrel A.V., Heard E. (2014). Noncoding RNAs and epigenetic mechanisms during X-chromosome inactivation. Annu. Rev. Cell Dev. Biol..

[bib32] Gkountela S., Zhang K.X., Shafiq T.A., Liao W.W., Hargan-Calvopina J., Chen P.Y., Clark A.T. (2015). DNA demethylation dynamics in the human prenatal germline. Cell.

[bib33] Guo F., Yan L., Guo H., Li L., Hu B., Zhao Y., Yong J., Hu Y., Wang X., Wei Y. (2015). The transcriptome and DNA methylome landscapes of human primordial germ cells. Cell.

[bib34] Gupta V., Parisi M., Sturgill D., Nuttall R., Doctolero M., Dudko O.K., Malley J.D., Eastman P.S., Oliver B. (2006). Global analysis of X-chromosome dosage compensation. J. Biol..

[bib35] Hall H., Hunt P., Hassold T. (2006). Meiosis and sex chromosome aneuploidy: how meiotic errors cause aneuploidy; how aneuploidy causes meiotic errors. Curr. Opin. Genet. Dev..

[bib36] Hayashi K., Ohta H., Kurimoto K., Aramaki S., Saitou M. (2011). Reconstitution of the mouse germ cell specification pathway in culture by pluripotent stem cells. Cell.

[bib37] Hayashi K., Ogushi S., Kurimoto K., Shimamoto S., Ohta H., Saitou M. (2012). Offspring from oocytes derived from in vitro primordial germ cell-like cells in mice. Science.

[bib38] Hilscher W. (1974). Kinetics of prespermatogenesis and spermatogenesis. Verh. Anat. Ges..

[bib39] Hughes J.F., Page D.C. (2015). The biology and evolution of mammalian Y chromosomes. Annu. Rev. Genet..

[bib40] Hunt P.A., Worthman C., Levinson H., Stallings J., LeMaire R., Mroz K., Park C., Handel M.A. (1998). Germ cell loss in the XXY male mouse: altered X-chromosome dosage affects prenatal development. Mol. Reprod. Dev..

[bib41] Ichijima Y., Sin H.S., Namekawa S.H. (2012). Sex chromosome inactivation in germ cells: emerging roles of DNA damage response pathways. Cell Mol. Life Sci..

[bib42] Inagaki A., Schoenmakers S., Baarends W.M. (2010). DNA double strand break repair, chromosome synapsis and transcriptional silencing in meiosis. Epigenetics.

[bib43] Julien P., Brawand D., Soumillon M., Necsulea A., Liechti A., Schutz F., Daish T., Grutzner F., Kaessmann H. (2012). Mechanisms and evolutionary patterns of mammalian and avian dosage compensation. PLoS Biol..

[bib44] Kaessmann H., Vinckenbosch N., Long M. (2009). RNA-based gene duplication: mechanistic and evolutionary insights. Nat. Rev. Genet..

[bib45] Khil P.P., Smirnova N.A., Romanienko P.J., Camerini-Otero R.D. (2004). The mouse X chromosome is enriched for sex-biased genes not subject to selection by meiotic sex chromosome inactivation. Nat. Genet..

[bib46] Khil P.P., Oliver B., Camerini-Otero R.D. (2005). X for intersection: retrotransposition both on and off the X chromosome is more frequent. Trends Genet..

[bib47] Kim D., Pertea G., Trapnell C., Pimentel H., Kelley R., Salzberg S.L. (2013). TopHat2: accurate alignment of transcriptomes in the presence of insertions, deletions and gene fusions. Genome Biol..

[bib48] Leitch H.G., Tang W.W., Surani M.A. (2013). Primordial germ-cell development and epigenetic reprogramming in mammals. Curr. Top. Dev. Biol..

[bib49] Lin H., Gupta V., Vermilyea M.D., Falciani F., Lee J.T., O'Neill L.P., Turner B.M. (2007). Dosage compensation in the mouse balances up-regulation and silencing of X-linked genes. PLoS Biol..

[bib50] Lin H., Halsall J.A., Antczak P., O'Neill L.P., Falciani F., Turner B.M. (2011). Relative overexpression of X-linked genes in mouse embryonic stem cells is consistent with Ohno's hypothesis. Nat. Genet..

[bib51] Lowe R., Gemma C., Rakyan V.K., Holland M.L. (2015). Sexually dimorphic gene expression emerges with embryonic genome activation and is dynamic throughout development. BMC Genomics.

[bib52] Mahadevaiah S.K., Odorisio T., Elliott D.J., Rattigan A., Szot M., Laval S.H., Washburn L.L., McCarrey J.R., Cattanach B.M., Lovell-Badge R. (1998). Mouse homologues of the human AZF candidate gene RBM are expressed in spermatogonia and spermatids, and map to a Y chromosome deletion interval associated with a high incidence of sperm abnormalities. Hum. Mol. Genet..

[bib53] Mahadevaiah S.K., Costa Y., Turner J.M. (2009). Using RNA FISH to study gene expression during mammalian meiosis. Methods Mol. Biol..

[bib54] McMahon A., Monk M. (1983). X-chromosome activity in female mouse embryos heterozygous for Pgk-1 and Searle's translocation, T(X; 16) 16H. Genet. Res..

[bib55] Mroz K., Carrel L., Hunt P.A. (1999). Germ cell development in the XXY mouse: evidence that X chromosome reactivation is independent of sexual differentiation. Dev. Biol..

[bib56] Mueller J.L., Mahadevaiah S.K., Park P.J., Warburton P.E., Page D.C., Turner J.M. (2008). The mouse X chromosome is enriched for multicopy testis genes showing postmeiotic expression. Nat. Genet..

[bib57] Mueller J.L., Skaletsky H., Brown L.G., Zaghlul S., Rock S., Graves T., Auger K., Warren W.C., Wilson R.K., Page D.C. (2013). Independent specialization of the human and mouse X chromosomes for the male germ line. Nat. Genet..

[bib58] Muller H.J. (1914). A factor for the fourth chromosome of *Drosophila*. Science.

[bib59] Muzumdar M.D., Tasic B., Miyamichi K., Li L., Luo L. (2007). A global double-fluorescent Cre reporter mouse. Genesis.

[bib60] Nguyen D.K., Disteche C.M. (2006). Dosage compensation of the active X chromosome in mammals. Nat. Genet..

[bib61] Ohno S. (1967). Sex Chromosomes and Sex-Linked Genes.

[bib62] Pessia E., Makino T., Bailly-Bechet M., McLysaght A., Marais G.A. (2012). Mammalian X chromosome inactivation evolved as a dosage-compensation mechanism for dosage-sensitive genes on the X chromosome. Proc. Natl. Acad. Sci. USA.

[bib63] Pessia E., Engelstadter J., Marais G.A. (2014). The evolution of X chromosome inactivation in mammals: the demise of Ohno's hypothesis?. Cell Mol. Life Sci..

[bib64] Picelli S., Faridani O.R., Bjorklund A.K., Winberg G., Sagasser S., Sandberg R. (2014). Full-length RNA-seq from single cells using Smart-seq2. Nat. Protoc..

[bib65] Ramskold D., Wang E.T., Burge C.B., Sandberg R. (2009). An abundance of ubiquitously expressed genes revealed by tissue transcriptome sequence data. PLoS Comput. Biol..

[bib66] Rohozinski J., Bishop C.E. (2004). The mouse juvenile spermatogonial depletion (jsd) phenotype is due to a mutation in the X-derived retrogene, mUtp14b. Proc. Natl. Acad. Sci. USA.

[bib67] Sadate-Ngatchou P.I., Payne C.J., Dearth A.T., Braun R.E. (2008). Cre recombinase activity specific to postnatal, premeiotic male germ cells in transgenic mice. Genesis.

[bib68] Saitou M. (2009). Germ cell specification in mice. Curr. Opin. Genet. Dev..

[bib69] Seisenberger S., Andrews S., Krueger F., Arand J., Walter J., Santos F., Popp C., Thienpont B., Dean W., Reik W. (2012). The dynamics of genome-wide DNA methylation reprogramming in mouse primordial germ cells. Mol. Cell.

[bib70] Straub T., Becker P.B. (2007). Dosage compensation: the beginning and end of generalization. Nat. Rev. Genet..

[bib71] Sugimoto M., Abe K. (2007). X chromosome reactivation initiates in nascent primordial germ cells in mice. PLoS Genet..

[bib72] Tang W.W., Dietmann S., Irie N., Leitch H.G., Floros V.I., Bradshaw C.R., Hackett J.A., Chinnery P.F., Surani M.A. (2015). A unique gene regulatory network resets the human germline epigenome for development. Cell.

[bib73] Trapnell C., Roberts A., Goff L., Pertea G., Kim D., Kelley D.R., Pimentel H., Salzberg S.L., Rinn J.L., Pachter L. (2012). Differential gene and transcript expression analysis of RNA-seq experiments with TopHat and Cufflinks. Nat. Protoc..

[bib74] Trapnell C., Hendrickson D.G., Sauvageau M., Goff L., Rinn J.L., Pachter L. (2013). Differential analysis of gene regulation at transcript resolution with RNA-seq. Nat. Biotechnol..

[bib75] Turner J.M. (2007). Meiotic sex chromosome inactivation. Development.

[bib76] Turner J.M., Mahadevaiah S.K., Fernandez-Capetillo O., Nussenzweig A., Xu X., Deng C.X., Burgoyne P.S. (2005). Silencing of unsynapsed meiotic chromosomes in the mouse. Nat. Genet..

[bib77] Vergouwen R.P., Jacobs S.G., Huiskamp R., Davids J.A., de Rooij D.G. (1991). Proliferative activity of gonocytes, Sertoli cells and interstitial cells during testicular development in mice. J. Reprod. Fertil..

[bib78] von Meyenn F., Berrens R.V., Andrews S., Santos F., Collier A.J., Krueger F., Osorno R., Dean W., Rugg-Gunn P.J., Reik W. (2016). Comparative principles of DNA methylation reprogramming during human and mouse in vitro primordial germ cell specification. Dev. Cell.

[bib79] Wang P.J. (2004). X chromosomes, retrogenes and their role in male reproduction. Trends Endocrinol. Metab..

[bib80] Wang P.J., Page D.C. (2002). Functional substitution for TAF(II)250 by a retroposed homolog that is expressed in human spermatogenesis. Hum. Mol. Genet..

[bib81] Wang P.J., McCarrey J.R., Yang F., Page D.C. (2001). An abundance of X-linked genes expressed in spermatogonia. Nat. Genet..

[bib82] Xiong Y., Chen X., Chen Z., Wang X., Shi S., Wang X., Zhang J., He X. (2010). RNA sequencing shows no dosage compensation of the active X-chromosome. Nat. Genet..

[bib83] Yan W., McCarrey J.R. (2009). Sex chromosome inactivation in the male. Epigenetics.

[bib84] Yildirim E., Sadreyev R.I., Pinter S.F., Lee J.T. (2012). X-chromosome hyperactivation in mammals via nonlinear relationships between chromatin states and transcription. Nat. Struct. Mol. Biol..

[bib85] Yin S., Deng W., Zheng H., Zhang Z., Hu L., Kong X. (2009). Evidence that the nonsense-mediated mRNA decay pathway participates in X chromosome dosage compensation in mammals. Biochem. Biophys. Res. Commun..

[bib86] Yoshimizu T., Sugiyama N., De Felice M., Yeom Y.I., Ohbo K., Masuko K., Obinata M., Abe K., Scholer H.R., Matsui Y. (1999). Germline-specific expression of the Oct-4/green fluorescent protein (GFP) transgene in mice. Dev. Growth Differ..

