# Supplementary material for: Non-Canonical and Sexually Dimorphic X Dosage Compensation States in the Mouse and Human Germline
Source: Dev Cell. 2017 Feb 6;40(3):289–301.e3. doi: 10.1016/j.devcel.2016.12.023 (PMC5300051; doi:10.1016/j.devcel.2016.12.023)
Supplement: Document S1. Figures S1–S5 and Tables S1 and S2 [file mmc1.pdf]

Developmental Cell, Volume *40*

Supplemental Information

Non-Canonical and Sexually Dimorphic X Dosage

Compensation States in the Mouse and Human Germline

Mahesh N. Sangrithi, Helene Royo, Shantha K. Mahadevaiah, Obah Ojarikre, Leena Bhaw, Abdul Sesay, Antoine H.F.M. Peters, Michael Stadler, and James M.A. Turner

INVENTORY OF SUPPLEMENTARY MATERIALS

Supplementary Figure 1 (Summarizes Method Details in the STAR Methods section).

Supplementary Figure 2 (Relates to Figure 1).

Supplementary Figure 3 (Relates to Figure 2; also see STAR methods).

Supplementary Figure 4 (Relates to Figure 3).

Supplementary Figure 5. Relative expression (FPKM) of X genes and their autosomal retrogene partners in XY male germ cells (Related to Figure 4).

Supplementary Table 1. Metrics of RNA-seq libraries used in this study (Related to Figure 1; refer to STAR methods (*Bioinformatic analysis*)).

Supplementary Table 2. Numbers and percentages of non-ubiquitous X and autosomal genes exhibiting FPKM ≥1 for samples shown in Figure 2A.

Supplementary Table 3. Ubiquitously expressed mouse genes - no upper FPKM threshold (Related to Figures 2D, 3B, 3D, 4B and 4D).

Supplementary Table 4. Ubiquitously expressed mouse genes - upper FPKM threshold (Related to Figures 2G, 3A, 3C, 4A and 4C).

Supplementary Table 5. Ubiquitously expressed human genes - no upper FPKM threshold (Related to Figures 5C and 5E).

Supplementary Table 6. Ubiquitously expressed human genes - upper FPKM threshold (Related to Figures 5B and 5D).

Supplementary Figure Legends:

Supplementary Figure 1 (Summarizes Method Details in the STAR Methods section).

Cartoon showing comparative time line of germ cell development and X chromosome activity status in mice and humans. Timing of expression of reporters used for mouse germ cell purification are also shown.

Supplementary Figure 2 (Relates to Figure 1)**.**

A) Two dimensional tSNE dimensionality reduction analysis of all samples in the dataset. tSNE was performed using the top 500 genes showing the highest variance across the samples. B) Matrix of replicate correlations. Spearmann correlations were calculated for biological replicates used in this study, and were used to plot the matrix shown.

Supplementary Figure 3 (Relates to Figure 2; also see STAR methods)**.**

X:A ratios in mouse ES and epiblast-like stem cells. SRA accession number of the datasets are stated in the figure. Male cells are underscored by a dark-blue bar and female ones by a pink bar.

Supplementary Figure 4 (Relates to Figure 3)**.**

X-chromosome activity charted in individual female epiblast cells and germ cells using *Xist* RNA FISH analysis. A). A cartoon depicting the X chromosome activity in female E6.5 epiblast, germ cells from E9.5, E11.5 and E14.5 embryos. B). (i) Epiblast cells (elongated white arrows), (ii) E9.5 migratory primordial germ cells (white arrows), E11.5 gonadal germ cells (white arrows) and E14.5 oogonia (white arrows). Asterisks denote somatic cells. C). Stacked bar-chart showing percentage of cells in each sample that were positive (blue) or negative (red) for *Xist* RNA FISH signals. A low percentage of female germ cells at E11.5 and E14.5 exhibited *Xist* RNA FISH

signals. This finding confirmed that these cells had reactivated their second X-chromosome. Numbers of cells counted in of the samples are stated at the top of each bar.

Supplementary Figure 5. Relative expression (FPKM) of X genes and their autosomal retrogene partners in XY male germ cells (Related to Figure 4).

Supplementary Table 1. Metrics of RNA-seq libraries used in this study (Related to Figure 1; refer to STAR methods (*Bioinformatic analysis*)).

Supplementary Table 2. Numbers and percentages of non-ubiquitous X and autosomal genes exhibiting FPKM ≥1 for samples shown in Figure 2A.

Supplementary Table 3. Ubiquitously expressed mouse genes - no upper FPKM threshold (Related to Figures 2D, 3B, 3D, 4B and 4D).

Supplementary Table 4. Ubiquitously expressed mouse genes - upper FPKM threshold (Related to Figures 2G, 3A, 3C, 4A and 4C).

Supplementary Table 5. Ubiquitously expressed human genes - no upper FPKM threshold (Related to Figures 5C and 5E).

Supplementary Table 6. Ubiquitously expressed human genes - upper FPKM threshold (Related to Figures 5B and 5D).

Supplementary Figure 1

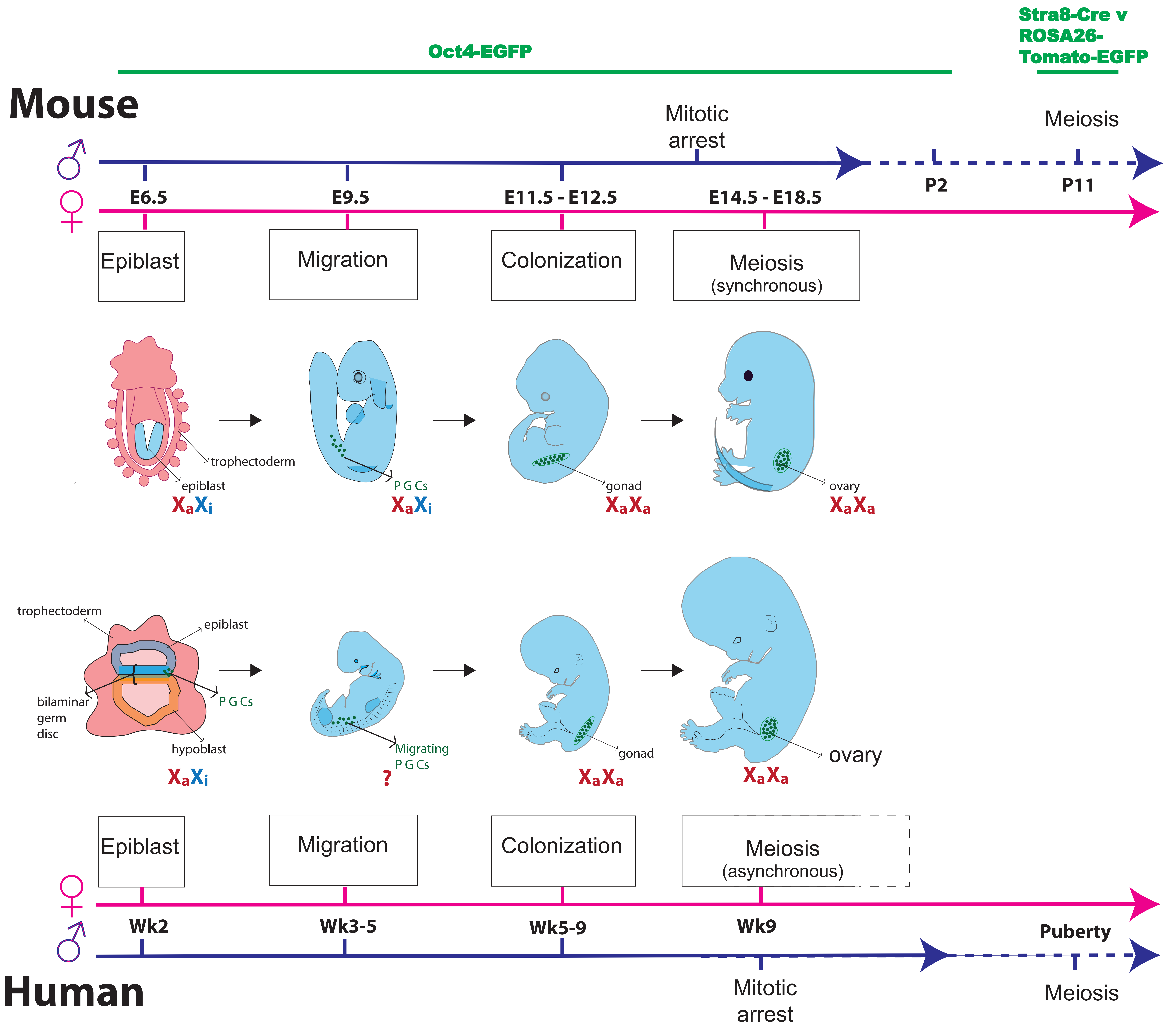

Supplementary Figure 2

A

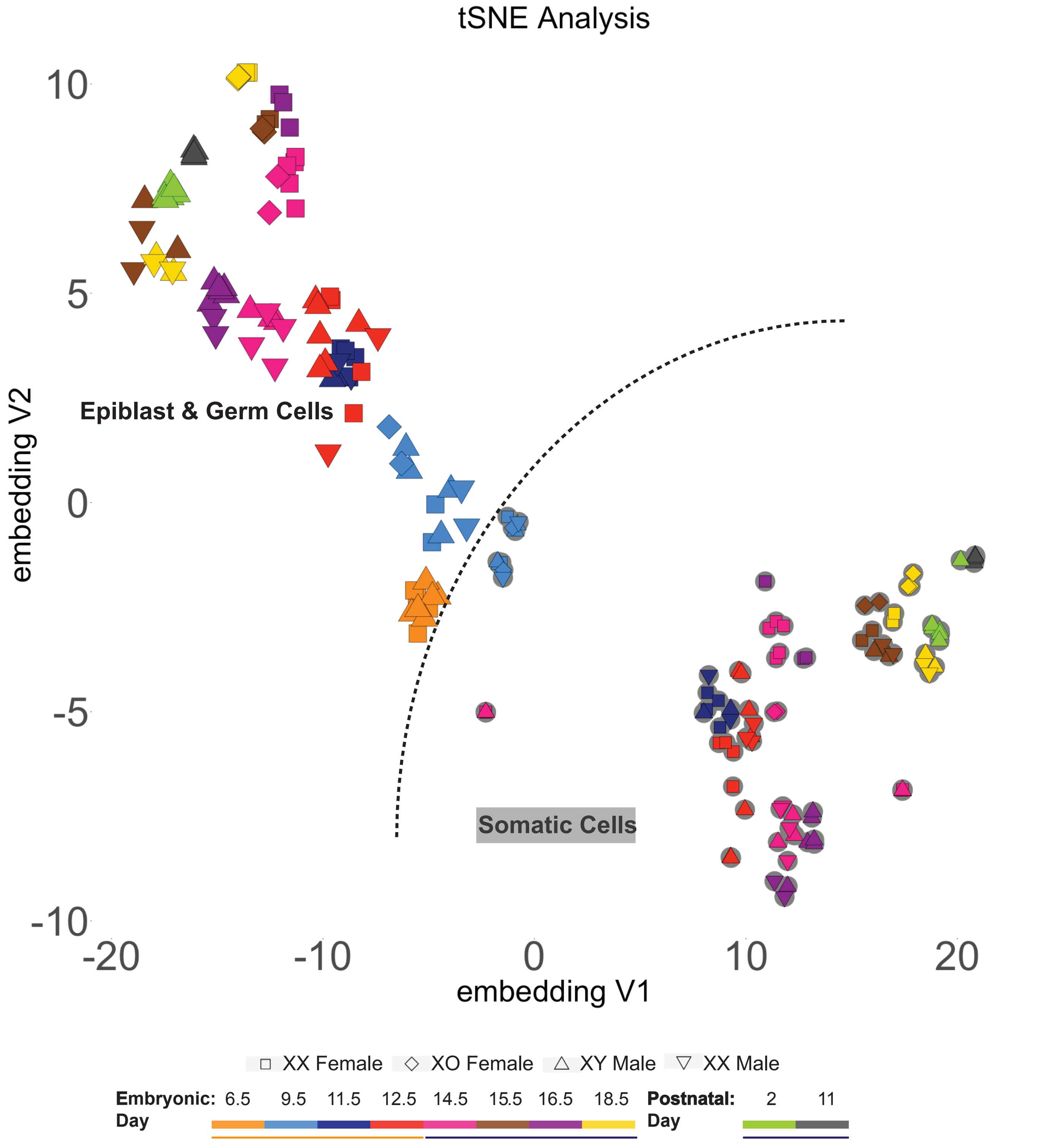

B

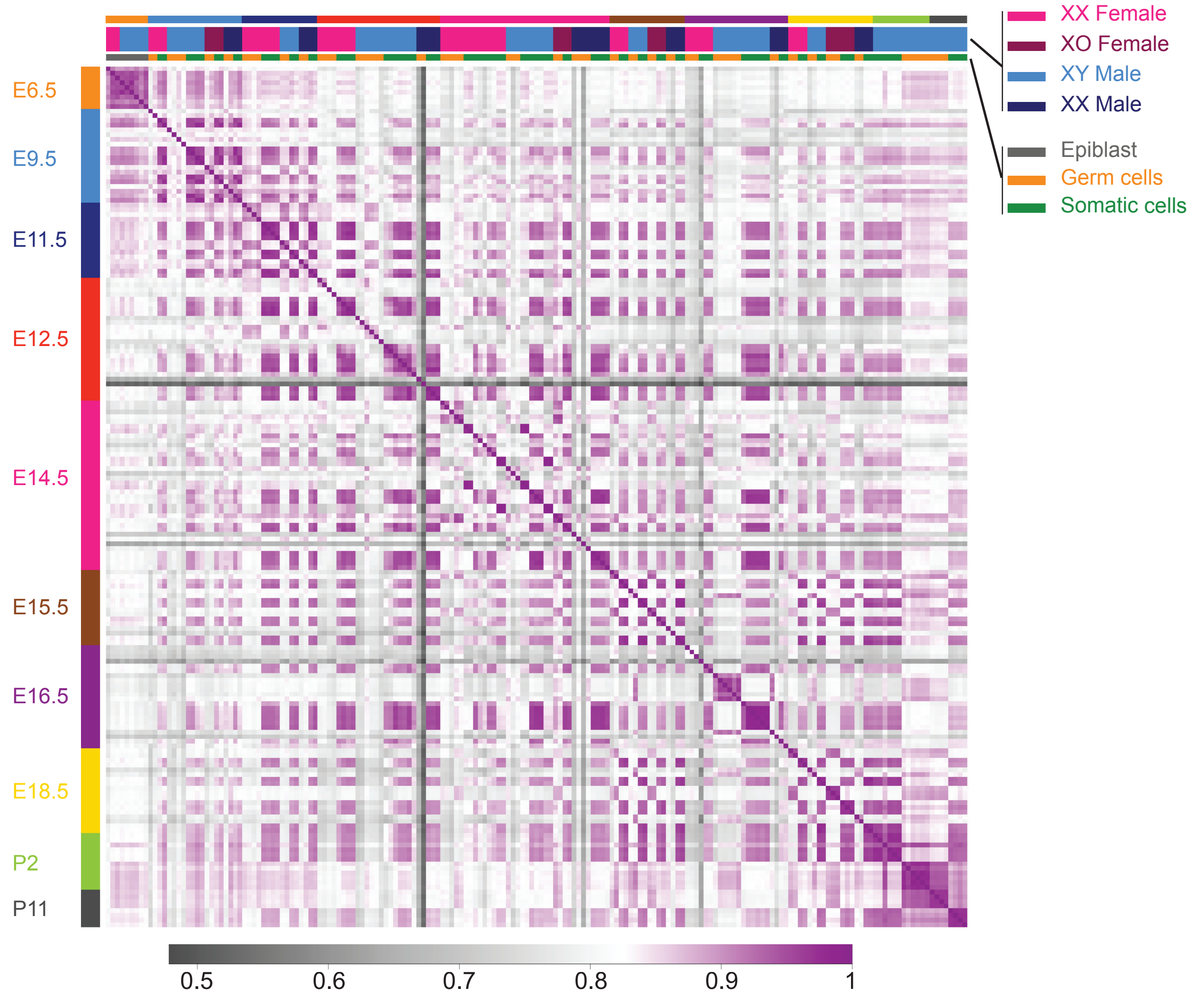

Supplementary Figure 3

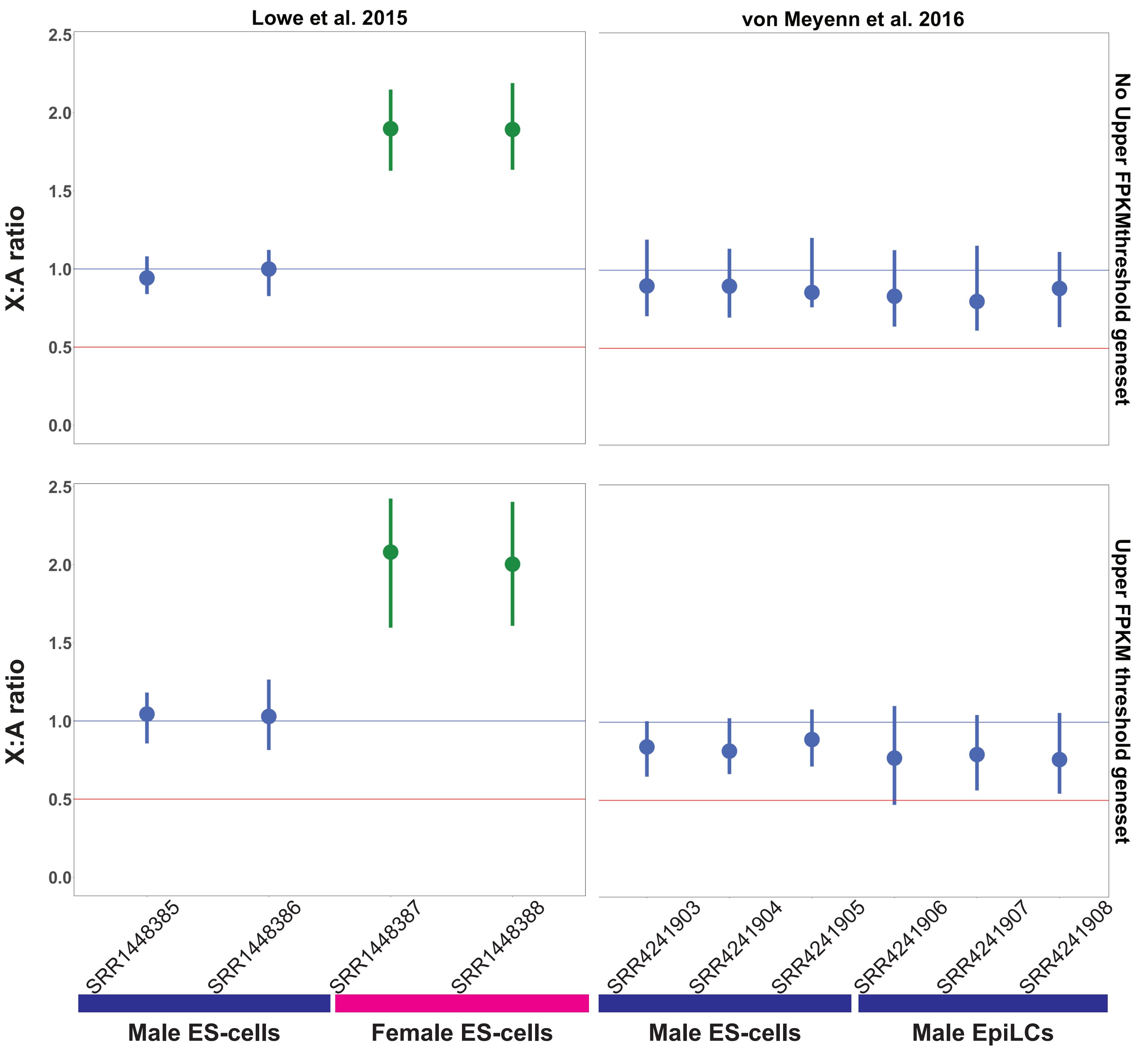

Supplementary Figure 4

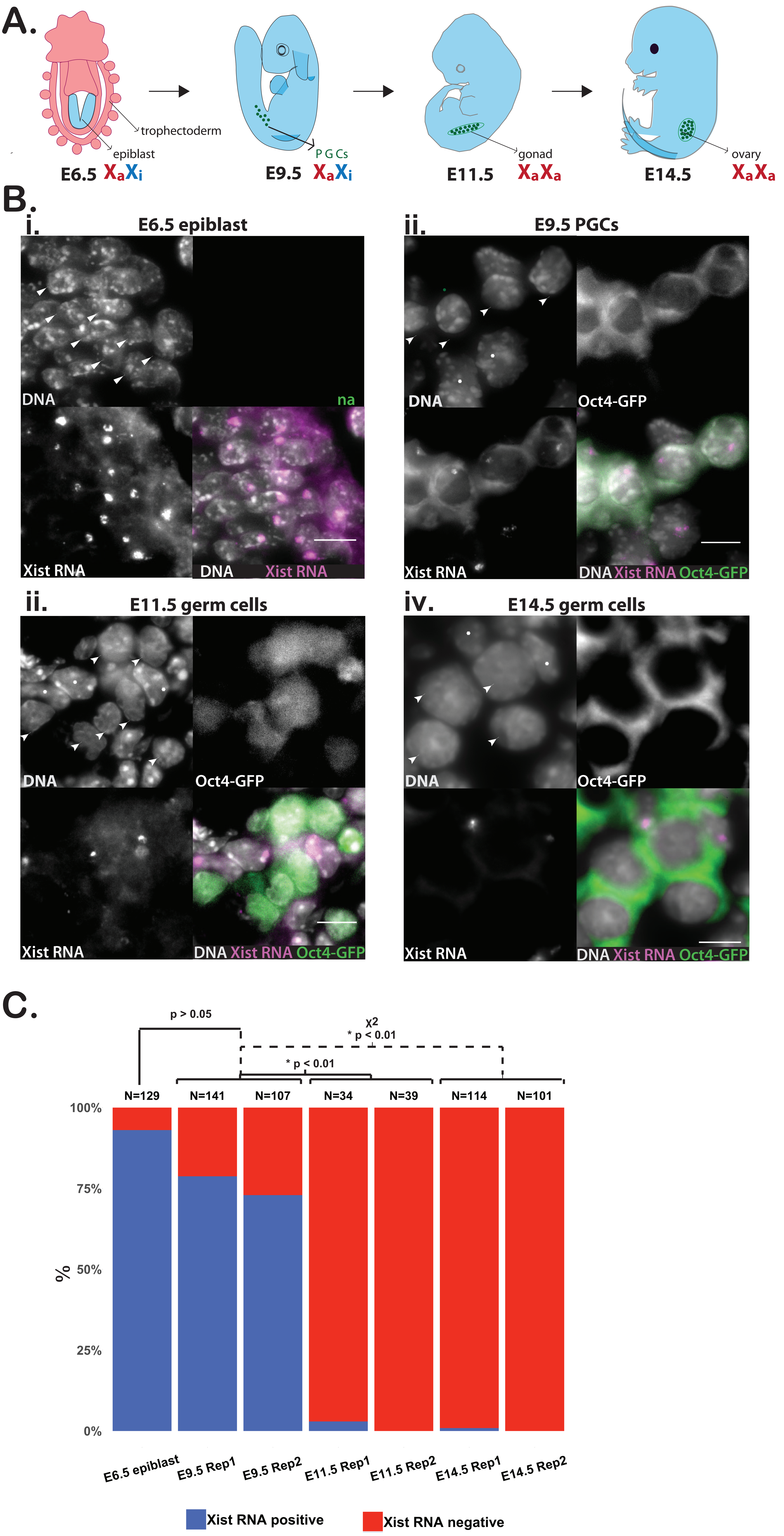

Supplementary Figure 5

A

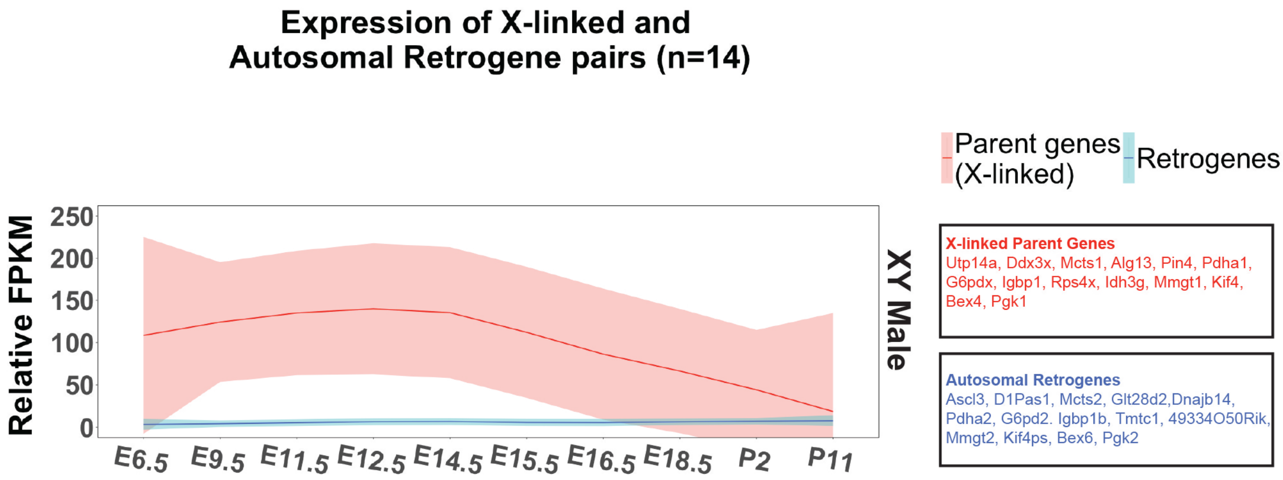

B

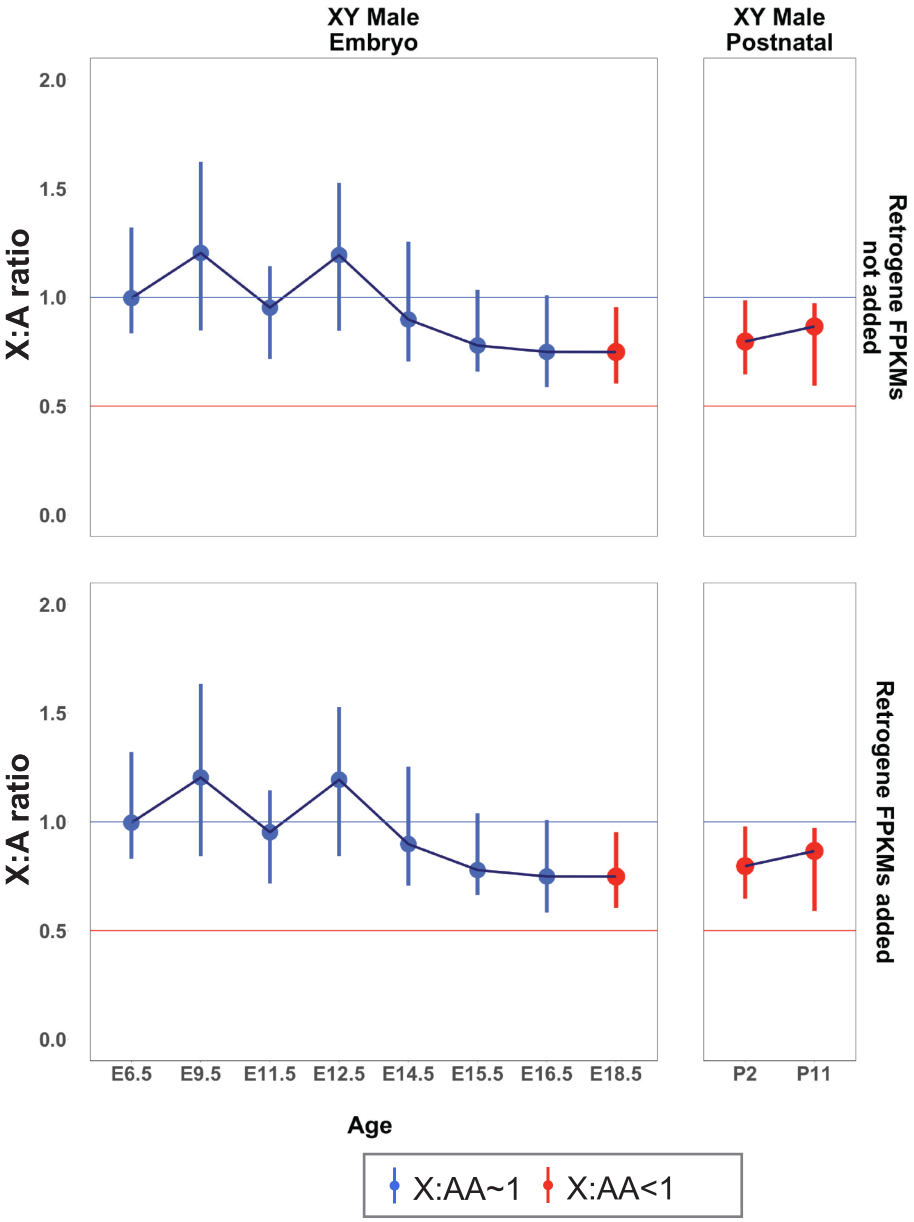

Supplementary Table 1

Samples	No. of Reads	Mapped	Overall read mapping rate %	Aligned pairs	Concordant pair alignment rate %
E9.5 XX F_GC_1	46050987	44723246	96.4	43258239	84.4
E9.5 XX F_GC_2	12272046	9243118	65.7	5454098	40.3
E9.5 XX F_SC_1	28386893	27003689	94.5	25648978	83
E9.5 XX F_SC_2	39713464	30943305	69.3	18167101	40.6
E9.5 XY M_GC_1	35965922	34418799	94.9	33117669	83.7
E9.5 XY M_GC_2	32802948	31399363	95	30300224	84.4
E9.5 XY M_GC_3	12132025	8094386	57.3	3901486	29.3
E9.5 XY M_GC_4	12043405	6643460	47.6	3540557	26.9
E9.5 XY M_SC_1	40406772	39405220	96.8	38192623	86
E9.5 XY M_SC_2	34355568	33592787	97	32540220	87.2
E9.5 XY M_SC_3	31512671	25420228	71.9	15780303	45.9
E9.5 XY M_SC_4	30045729	23386215	69.2	13729884	39
E9.5 XO F_GC_1	37914973	36484288	95.5	35032264	84
E9.5 XO F_GC_2	8963060	6278449	61.5	3509494	35.2
E9.5 XO F_SC_1	34802214	32533136	94.8	31498499	84.3
E9.5 XO F_SC_2	18415941	14009047	66.7	7804824	38.4
E9.5 XX M_GC_1	43964197	42393202	95.9	40807530	84.4
E9.5 XX M_GC_2	24306023	17943254	64.7	9708158	35.1
E9.5 XX M_SC_1	30513797	29558243	96.3	28457328	85
E9.5 XX M_SC_2	35128536	26768732	68.4	15890052	39.4
E11.5 XX F_GC_1	73772964	68591692	87	57518202	72.4
E11.5 XX F_GC_2	34691212	32350831	92	30206793	81.5
E11.5 XX F_GC_3	34632289	32189351	91.6	30797381	81.2
E11.5 XX F_GC_4	48389242	44559132	90.6	42332211	79
E11.5 XX F_SC_1	101557389	95261714	91.5	89686788	82.6
E11.5 XX F_SC_2	42827118	41192689	94.7	38825189	85.7
E11.5 XX F_SC_3	40658848	37854016	90.5	34249747	80.6
E11.5 XX F_SC_4	37396111	35343045	93.1	33890872	85.5
E11.5 XY M_GC_1	33854553	30771895	89	28341334	78.6
E11.5 XY M_GC_2	42203053	39697612	91.7	36424848	81
E11.5 XY M_SC_1	33674860	30983184	88.2	28109938	79.1
E11.5 XY M_SC_2	39919065	37140265	90.8	33765986	79.3
E11.5 XX M_GC_1	59879438	55967272	91.4	52626402	79.6
E11.5 XX M_GC_2	46968013	44044077	91.8	40631593	81.2
E11.5 XX M_SC_1	82063958	77603361	92.4	73359684	83.7
E11.5 XX M_SC_2	59220762	56706050	94.3	54415364	86.4
E12.5 XX F_GC_1	24889038	23011598	92	2238340	83.3
E12.5 XX F_GC_2	23877394	20770051	86.4	20198415	79.5
E12.5 XX F_GC_3	29595261	24276197	78.8	22034536	68.1
E12.5 XX F_GC_4	19184826	17045783	85.6	15572621	74
E12.5 XX F_SC_1	37656653	35296548	93.1	34420154	86.3

E12.5 XX F_SC_2	40697757	39031619	95.5	38245346	88.1
E12.5 XX F_SC_3	41140216	39440860	94.6	37960715	84.8
E12.5 XX F_SC_4	53578777	47892570	85.3	43364536	75.9
E12.5 XY M_GC_1	27962154	23874580	85	23160407	76.2
E12.5 XY M_GC_2	22147573	18487970	84	17824661	74.2
E12.5 XY M_GC_3	44748739	42812390	95.2	41848301	86.6
E12.5 XY M_GC_4	33340239	29120956	84.6	26900294	75.4
E12.5 XY M_GC_5	23070443	19383093	81.3	17899313	72
E12.5 XY M_GC_6	42084166	39484187	92.6	37948967	81.3
E12.5 XY M_SC_1	36271525	34502780	94.7	33733751	85.8
E12.5 XY M_SC_2	33733751	34252766	94.3	33407328	85.3
E12.5 XY M_SC_3	34724307	33354940	95.6	32706051	88
E12.5 XY M_SC_4	38771368	35673063	88.6	32723615	79.4
E12.5 XY M_SC_5	57732271	51842560	87.1	48262175	78.5
E12.5 XY M_SC_6	55926177	53325614	94.1	51207143	83.5
E12.5 XY M_SC_7	59473246	56641939	94	54381277	81.5
E12.5 XX M_GC_1	45296228	42615943	92.8	40838252	80.9
E12.5 XX M_GC_2	36182313	32421491	88.3	31111659	78.6
E12.5 XX M_SC_1	55321814	52929254	94.2	50560078	82.8
E12.5 XX M_SC_2	61733520	59190346	94.5	56729164	83.1
E12.5 XX M_SC_3	56729164	42666594	94.5	41018816	83.9
E14.5 XX F_GC_1	41018816	39702042	86.3	38449446	78.7
E14.5 XX F_GC_2	32428012	29868949	91.6	28981396	82.8
E14.5 XX F_GC_3	13545004	13036597	95.6	12738072	87.5
E14.5 XX F_GC_4	48548985	46968449	96.1	45963824	88.3
E14.5 XX F_GC_5	30482637	28968429	94.2	28226291	86.8
E14.5 XX F_SC_1	28684766	24567640	85.2	23829681	77.6
E14.5 XX F_SC_2	34532659	31053730	89.5	30288871	82.5
E14.5 XX F_SC_3	33131758	31843685	95.6	31183314	86.9
E14.5 XX F_SC_4	73635998	71187000	96	69555969	87.7
E14.5 XX F_SC_5	42493965	40454694	94.7	39059815	8.6
E14.5 XX F_Liver_1	48128368	46041897	94.8	44313655	85.5
E14.5 XX F_Liver_2	38376194	35319990	92.1	34088571	85.1
E14.5 XX F_Tail_1	37997850	36370339	95.4	35427812	89.7
E14.5 XX F_Tail_2	37995367	35728020	92.9	33729043	82.5
E14.5 XY M_GC_1	34616031	21053355	60.4	20284101	53.2
E14.5 XY M_GC_2	41199611	39244561	94.6	38318778	84.2
E14.5 XY M_GC_3	46688710	44218035	94	43053816	84.2
E14.5 XY M_SC_1	34496700	31823754	91.7	30836924	83.5
E14.5 XY M_SC_2	43689113	41979491	95.3	40924235	86.6
E14.5 XY M_SC_3	46679089	44764624	95	39750456	73.2
E14.5 XY M_Liver_1	34755780	32880912	94.4	31879420	88.6
E14.5 XY M_Liver_2	38195831	34512065	89.8	31837925	73.7
E14.5 XY M_Tail_1	35390365	33853408	95.4	33003213	89.8
E14.5 XY M_Tail_2	43067474	40882344	94.4	39158762	84.1
E14.5 XO F_GC_1	47098681	45385531	95.8	44086503	86.8

E14.5 XO F_GC_2	38041178	36440676	95.2	35366796	85.5
E14.5 XO F_SC_1	39239386	37949572	96	36834229	89.1
E14.5 XO F_SC_2	32480821	30990535	95	30115913	83.9
E14.5 XX M_GC_1	26601186	23440005	87.7	22831328	78.6
E14.5 XX M_GC_2	27935387	19958229	71	19273160	63.7
E14.5 XX M_GC_3	46478194	44289447	94.7	43271076	85.7
E14.5 XX M_GC_4	41324593	39049794	93.7	37993740	85
E14.5 XX M_SC_1	29361836	27950598	94.7	27192328	86
E14.5 XX M_SC_2	54993866	51931134	94	50491174	85.7
E14.5 XX M_SC_3	45769709	43878136	95.3	42913247	87.8
E14.5 XX M_SC_4	36990417	35140204	94.5	34341003	86.3
E15.5 XX F_GC_1	24562858	23583586	94.7	22454190	83.2
E15.5 XX F_GC_2	30126159	29140055	95.5	27844432	84.4
E15.5 XX F_SC_1	28514601	26796037	92.6	24837749	81.4
E15.5 XX F_SC_2	32215715	31082466	95.1	29495233	84.6
E15.5 XY M_GC_1	22999653	21811724	93.5	20599928	78.7
E15.5 XY M_GC_2	36692459	3535122	94.9	33615059	83.8
E15.5 XY M_SC_1	28423766	27239708	94.5	25683169	84
E15.5 XY M_SC_2	15787172	4072125	24.6	2597719	11.5
E15.5 XO F_GC_1	17209494	12005881	67.5	8841721	47
E15.5 XO F_GC_2	22322724	12475591	53.8	8482879	34.4
E15.5 XO F_SC_1	14871538	9258070	59.2	5902126	37.4
E15.5 XO F_SC_2	1387034	9561488	65.5	6188434	42.1
E15.5 XX M_GC_1	13084008	12278518	92.5	11616017	78.1
E15.5 XX M_GC_2	10748356	10009585	91.7	9456938	79.6
E15.5 XX M_SC_1	25704154	2462872	94.5	23249523	84.2
E15.5 XX M_SC_2	19185459	6091071	30.5	3946978	14
E16.5 XX F_GC_1	37033925	33920950	90.9	32977946	81.8
E16.5 XX F_GC_2	48735981	43025796	87.4	41461471	78.5
E16.5 XX F_GC_3	37249217	27382726	72.7	26304026	65.7
E16.5 XX F_SC_1	28931301	23476810	80.8	22971189	74.6
E16.5 XX F_SC_2	50347998	45699607	89.9	44207323	82.5
E16.5 XX F_SC_3	45708379	42300228	91.8	41085580	82.8
E16.5 XY M_GC_1	33701593	31124317	91.9	30236405	83
E16.5 XY M_GC_2	42243377	39675055	93.2	38653985	86
E16.5 XY M_GC_3	41666965	39587020	94.5	38658827	86.2
E16.5 XY M_GC_4	46730071	44367965	94.4	43350655	85.8
E16.5 XY M_GC_5	34303651	29570514	85.6	2853436	77.1
E16.5 XY M_GC_6	47255051	4340570	91	42002524	82.1
E16.5 XY M_SC_1	36359008	34550460	94.5	33712084	85.6
E16.5 XY M_SC_2	44795431	41823387	95	41823387	87.5
E16.5 XY M_SC_3	51011762	48915913	95.3	47809968	86.8
E16.5 XY M_SC_4	49824698	47769959	95.1	46595308	86.8
E16.5 XY M_SC_5	51821711	49803136	95.3	48617264	86.4
E16.5 XY M_SC_6	56368532	53670090	94.5	52440733	86.8
E16.5 XX M_GC_1	35841408	33691571	93.6	32812276	85.7

E16.5 XX M_GC_2	33405218	30398339	90.6	29462370	81
E16.5 XX M_SC_1	34665737	33574729	95.5	31965371	85.6
E16.5 XX M_SC_2	31308282	27756906	88.1	26779878	81
E18.5 XX F_GC_1	25377373	24415984	94.8	23255643	81.3
E18.5 XX F_GC_2	20402095	19550930	94.4	18628334	80.3
E18.5 XX F_SC_1	23560761	22543112	94.2	21299141	84.3
E18.5 XX F_SC_2	24122318	22962466	93.8	21753605	83.4
E18.5 XY M_GC_1	18906068	17823036	92.9	16820899	81
E18.5 XY M_GC_2	20423787	19512366	94.2	18504354	82.3
E18.5 XY M_SC_1	34665737	33574729	95.5	31965371	85.6
E18.5 XY M_SC_2	25638416	24846300	95.6	23680270	85.5
E18.5 XO F_GC_1	16431599	10664402	62.7	7646527	42.1
E18.5 XO F_GC_2	21088612	14130776	64.5	9692248	42.1
E18.5 XO F_GC_3	25136687	17018070	65.5	12382340	45.1
E18.5 XO F_SC_1	14820024	10121610	66.2	7137610	43.7
E18.5 XO F_SC_2	22662925	14779217	62.7	10044528	40
E18.5 XO F_SC_3	18261077	11505744	60.7	8028450	40.1
E18.5 XX M_GC_1	17191493	16403698	94	15494336	81.7
E18.5 XX M_GC_2	17703494	16090331	89.7	15273967	78.1
E18.5 XX M_SC_1	25718808	24946892	95.6	23724475	85.6
E18.5 XX M_SC_2	31407625	30531677	95.8	29127649	86.3
P2 XY M_GC_1	18583157	12706584	65.7	8872046	44.7
P2 XY M_GC_2	3997290	2322648	55.6	1551579	36.1
P2 XY M_GC_3	30046869	20103504	64.3	13936989	43.5
P2 XY M_GC_4	29534347	23537047	70.7	14431119	45.5
P2 XY M_GC_5	21985954	16737085	67.4	9891983	41
P2 XY M_GC_6	18359348	14248641	68.6	8494578	41.7
P2 XY M_SC_1	13302158	8466447	60.8	5696896	39.9
P2 XY M_SC_2	11518279	8115387	67.8	5799549	46.7
P2 XY M_SC_3	27273713	17882296	62.9	12163666	41.7
P2 XY M_SC_4	27862693	23080045	73.1	14502515	48.1
P2 XY M_SC_5	18359348	14248641	68.6	8494578	41.7
P2 XY M_SC_6	25726046	21123945	73.3	13449903	48.5
P11 XY M_GC_1	13302526	9393124	68.2	6085352	41
P11 XY M_GC_2	12677352	9263228	71	6280926	45.8
P11 XY M_GC_3	13472876	9366075	67.3	6048895	39.9
P11 XY M_GC_4	13198601	9443830	69.4	6261337	41.9
P11 XY M_SC_1	17570170	13575601	74.4	9659348	50
P11 XY M_SC_2	13375021	10206036	74.4	7300346	50.5
P11 XY M_SC_3	15625677	11454370	71.5	7888115	45.1
P11 XY M_SC_4	12599227	9124244	70.5	6163872	44.1
E6.5 XX F_1	17663893	1527982	8.8	202764	1.1
E6.5 XX F_2	16736555	4616881	26	660576	3.6
E6.5 XX F_3	21720331	4836561	20.8	794618	3.3
E6.5 XX F_4	16734020	7049084	41.5	3520223	19.3
E6.5 XY M_1	24876254	12463271	47.9	4257756	15.8

E6.5 XY M_2	30819788	19591773	60.8	9557711	28.4
E6.5 XY M_3	34004398	19434483	54.9	8412661	22.7
E6.5 XY M_4	23118529	8312502	34.5	5293986	19.6
E6.5 XY M_5	11416541	4552555	38.8	2460959	14.9
E6.5 XY M_6	19515827	4287107	20.5	900422	4.2

Supplementary Table 2

Chrom_class	Condition	No. of genes FPKM>1	% Percentage of genes
X-CH	E9.5.XY.M_SC	375	36.90944882
X-CH	E11.5.XY.M_SC	415	40.84645669
X-CH	E12.5.XY.M_SC	464	45.66929134
X-CH	E14.5.XY.M_SC	438	43.11023622
X-CH	E15.5.XY.M_SC	466	45.86614173
X-CH	E16.5.XY.M_SC	437	43.01181102
X-CH	E18.5.XY.M_SC	455	44.78346457
X-CH	P2_SC	480	47.24409449
X-CH	P11_SC	516	50.78740157
X-CH	E9.5.XX.F_SC	383	37.69685039
X-CH	E11.5.XX.F_SC	426	41.92913386
X-CH	E12.5.XX.F_SC	444	43.7007874
X-CH	E14.5.XX.F_SC	504	49.60629921
X-CH	E15.5.XX.F_SC	473	46.55511811
X-CH	E16.5.XX.F_SC	457	44.98031496
X-CH	E18.5.XX.F_SC	474	46.65354331
X-CH	E14.5.XX.F_liver	371	36.51574803
X-CH	E14.5.XY.M_liver	376	37.00787402
X-CH	E14.5.XX.F_tail	462	45.47244094
X-CH	E14.5.XY.M_tail	457	44.98031496
Autosome	E9.5.XY.M_SC	10424	47.31299927
Autosome	E11.5.XY.M_SC	11343	51.48420479
Autosome	E12.5.XY.M_SC	13129	59.5905955
Autosome	E14.5.XY.M_SC	12384	56.20915033
Autosome	E15.5.XY.M_SC	12814	58.16085694
Autosome	E16.5.XY.M_SC	12192	55.33769063
Autosome	E18.5.XY.M_SC	12326	55.94589688
Autosome	P2_SC	12701	57.64796659
Autosome	P11_SC	12615	57.25762527
Autosome	E9.5.XX.F_SC	10628	48.2389252
Autosome	E11.5.XX.F_SC	11536	52.36020334
Autosome	E12.5.XX.F_SC	12006	54.49346405
Autosome	E14.5.XX.F_SC	13387	60.76161946
Autosome	E15.5.XX.F_SC	13040	59.18663762
Autosome	E16.5.XX.F_SC	12665	57.4845679
Autosome	E18.5.XX.F_SC	13011	59.05501089
Autosome	E9.5.XX.M_SC	10364	47.04066812
Autosome	E14.5.XX.F_liver	11333	51.43881627
Autosome	E14.5.XY.M_liver	11359	51.55682643
Autosome	E14.5.XX.F_tail	12804	58.11546841
Autosome	E14.5.XY.M_tail	12720	57.73420479
