# Supplementary material for: Non-Canonical and Sexually Dimorphic X Dosage Compensation States in the Mouse and Human Germline
Source: Dev Cell. 2017 Feb 6;40(3):289–301.e3. doi: 10.1016/j.devcel.2016.12.023 (PMC5300051; doi:10.1016/j.devcel.2016.12.023)
Supplement: Document S2. Article plus Supplemental Information [file mmc6.pdf]

Developmental Cell

Non-Canonical and Sexually Dimorphic X Dosage Compensation States in the Mouse and Human Germline

Highlights

- X dosage compensation in germ cells is reset during GWR
- PGCs exhibit X upregulation before GWR, in keeping with Ohno's hypothesis
- X upregulation is lost during GWR
- Mouse and human germ cells exhibit X dosage states that are sexually dimorphic

Authors

Mahesh N. Sangrithi, Helene Royo, Shantha K. Mahadevaiah, ..., Antoine H.F.M. Peters, Michael Stadler, James M.A. Turner

Correspondence

james.turner@crick.ac.uk

In Brief

Germ cells reset their epigenome and transcriptome prior to meiosis. Sangrithi et al. show that unique X chromosome dosage compensation states prevail in germ cells. These states are determined by the number of X chromosomes present rather than phenotypic sex, providing a different perspective on infertility associated with sex chromosome aneuploidy.

Data Resources

E-MTAB-4616

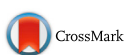

Non-Canonical and Sexually Dimorphic X Dosage Compensation States in the Mouse and Human Germline

Maresh N. Sangrithi,^{1,4} Helene Royo,^{2,3} Shantha K. Mahadevaiah,¹ Obah Ojarikre,¹ Leena Bhaw,¹ Abdul Sesay,¹ Antoine H.F.M. Peters,² Michael Stadler,^{2,3} and James M.A. Turner^{1,5,*}

¹Mill Hill Laboratory, The Francis Crick Institute, The Ridgeway, Mill Hill, London NW7 1AA, UK

²Friedrich Miescher Institute for Biomedical Research (FMI), 4058 Basel, Switzerland

³Swiss Institute of Bioinformatics, 4058 Basel, Switzerland

⁴UCL EGA Institute for Women's Health UCL, Medical School Building, 74 Huntley Street, London WC1E 6AU, UK

⁵Lead Contact

*Correspondence: james.turner@crick.ac.uk

<http://dx.doi.org/10.1016/j.devcel.2016.12.023>

SUMMARY

Somatic X dosage compensation requires two mechanisms: X inactivation balances X gene output between males (XY) and females (XX), while X upregulation, hypothesized by Ohno and documented *in vivo*, balances X gene with autosomal gene output. Whether X dosage compensation occurs in germ cells is unclear. We show that mouse and human germ cells exhibit non-canonical X dosage states that differ from the soma and between the sexes. Prior to genome-wide reprogramming, X upregulation is present, consistent with Ohno's hypothesis. Subsequently, however, it is erased. In females, erasure follows loss of X inactivation, causing X dosage excess. Conversely, in males, erasure leads to permanent X dosage decompensation. Sex chromosomally abnormal models exhibit a "sex-reversed" X dosage state: XX males, like XX females, develop X dosage excess, while XO females, like XY males, develop X dosage decompensation. Thus, germline X dosage compensation states are determined by X chromosome number, not phenotypic sex. These unexpected differences in X dosage compensation states between germline and soma offer unique perspectives on sex chromosome infertility.

INTRODUCTION

Male and female mammals carry the same complement of autosomes but differ with respect to their sex chromosomes: females have two X chromosomes (XX) while males have one X chromosome and one Y chromosome (XY). The X and Y chromosomes evolved from a pair of ancestral autosomes following the acquisition of the male-determining locus *Sry* on the proto-Y chromosome. The subsequent appearance of sexually antagonistic alleles near *Sry* caused progressive suppression of X-Y recombination (Bachtrog, 2013; Cortez et al., 2014; Hughes and Page, 2015; Muller, 1914). The X chromosome managed

to retain most of its ancestral genes through ongoing X-X recombination in the female germline. In contrast, without a partner with which to recombine, the Y chromosome lost most of its original gene content through genetic drift (Charlesworth, 1996; Charlesworth and Charlesworth, 2000).

Evolutionary loss of genes from the Y chromosome led to a disparity in the dosage of X chromosome versus autosomal genes, with males becoming monosomic for X-linked gene products. Susumu Ohno proposed that to rectify this imbalance, expression of X chromosome genes was increased 2-fold to match the output of the diploid autosomal complement, i.e., giving an X-to-autosome ratio (X:A) of 1 (termed Ohno's hypothesis) (Ohno, 1967). This process, X chromosome upregulation, was also acquired in females, leading to a 2-fold excess in X gene expression compared with males. To equalize this resulting sex difference in X gene output, mammals subsequently evolved X chromosome inactivation, the global silencing of one of the two X chromosomes in females (Dupont and Gribnau, 2013; Gendrel and Heard, 2014). Together, X upregulation and X inactivation ensure equalization of gene dosage both within, and between, the sexes.

Consistent with Ohno's hypothesis, X upregulation has been observed in multiple organisms including *Drosophila melanogaster* (Conrad and Akhtar, 2011; Gelbart and Kuroda, 2009; Straub and Becker, 2007), *Caenorhabditis elegans* (Gupta et al., 2006), and mammals (Adler et al., 1997; Gupta et al., 2006; Lin et al., 2007; Nguyen and Disteche, 2006). More recently, RNA-sequencing (RNA-seq) analyses showed that the X:A ratio in males and females is nearer 0.5, and therefore that X upregulation does not occur (Julien et al., 2012; Xiong et al., 2010). The discrepancy between these studies has been attributed to the choice of genes used to assay X upregulation. The X chromosome is enriched in tissue-specific genes, including those expressed in the testis and ovary (Deng et al., 2014; Khil et al., 2004; Mueller et al., 2008, 2013). These genes are silent in the soma, and thus their inclusion can artificially lower estimations of the somatic X:A ratio (Deng et al., 2011). A reappraisal of X:A ratios using expression thresholds that exclude such genes has confirmed the existence of X upregulation (Deng et al., 2011; Lin et al., 2011; Yildirim et al., 2012), and mechanistic studies have identified transcriptional and post-transcriptional mechanisms by which upregulation is achieved (Deng et al., 2013; Faucillon and Larsson, 2015; Yildirim et al., 2012; Yin et al.,

2009). X upregulation preferentially affects a subset of expressed X genes with dosage-sensitive housekeeping functions (Birchler, 2012; Pessia et al., 2012, 2014).

To date, studies of X upregulation have focused on somatic tissues, and it is therefore unclear whether germ cells also conform to Ohno's hypothesis. In mice, primordial germ cells (PGCs) arise from the post-implantation epiblast and migrate along the hindgut endoderm before colonizing the gonad. During this time, they undergo genome-wide reprogramming in which the pluripotency gene network is reactivated, somatic genes are repressed, and genomic imprints are removed (Gkoutela et al., 2015; Guo et al., 2015; Leitch et al., 2013; Seisenberger et al., 2012; Tang et al., 2015). In females, one of the two X chromosomes is already inactive prior to PGC specification (Hayashi et al., 2012; McMahon and Monk, 1983; Sugimoto and Abe, 2007). During germline reprogramming, the inactive X chromosome is subsequently reactivated (Chuva de Sousa Lopes et al., 2008; de Napoles et al., 2007; Sugimoto and Abe, 2007). However, expression from the active X chromosome during and after reprogramming has not been examined, and therefore the status of X dosage compensation throughout male and female germline development is unclear.

To address this point, we have generated extensive RNA-seq datasets from wild-type XY male and XX female, as well as sex chromosomally abnormal XO female (Turner syndrome) and XX male (Klinefelter syndrome variant) mouse germ cells before, during, and after reprogramming. Consistent with Ohno's hypothesis, early male and female germ cells exhibit upregulation of the active X chromosome. Later, however, they display unusual and sexually dimorphic dosage compensation patterns. Female germ cells exhibit a phase of X dosage excess, during which X:A ratios exceed 1, while male germ cells, conversely, exhibit X dosage decompensation, with X:A ratios falling below 1. These X dosage compensation patterns are conserved in human germ cells. Intriguingly, sex chromosome variant mice manifest a "sex-reversed" dosage compensation state: XO female germ cells become dosage decompensated like XY males, while XX male germ cells exhibit X dosage excess like XX females. Our studies reveal important differences in X dosage compensation states between the germline and soma and provide fresh insight into the etiology of subfertility caused by sex chromosome abnormalities.

RESULTS

Creation of Germ Cell and Somatic Cell RNA-Seq Datasets for Analysis of X Dosage Compensation

For our analysis, we wished to track X dosage compensation states throughout the entirety of embryonic germ cell development (Figure S1). In mice, PGCs arise from the post-implantation epiblast at embryonic day 7.25 (E7.25) (Saitou, 2009). These PGCs migrate along the hindgut endoderm and colonize the gonad between E10.5 and E11.5 (Saitou, 2009). Following sex determination at E11.5, germ cells develop in a sexually dimorphic manner. In females, they divide mitotically until E12.5, before entering meiosis. In males, in contrast, germ cells divide mitotically until E16.5, when they undergo quiescence at the G₀ phase of the cell cycle (Hilscher, 1974; Vergouwen et al.,

1991). After birth, they resume mitosis and enter meiosis at around postnatal day 10 (P10).

As a baseline reflecting the epigenetic state of PGC and somatic cell precursors, we isolated E6.5 epiblasts (Seisenberger et al., 2012). For later time points, we used mice carrying a previously described Oct4-EGFP transgene (Yoshimizu et al., 1999). We used fluorescence-activated cell sorting (FACS) to collect highly purified populations of germ cells (EGFP-positive) and gonadal somatic cells (EGFP-negative) from both sexes at E9.5, E11.5, E12.5, E14.5, E15.5, E16.5, and E18.5, as well as purified spermatogonia at P2. For purification of leptotene/zygotene spermatocytes at P11, we used an alternative reporter, ROSA26-Tomato-EGFP (Muzumdar et al., 2007) carrying a Stra8-Cre transgene (Sadate-Ngatchou et al., 2008). Non-gonadal somatic cell control datasets were generated from E14.5 male and female liver and tail. Germ cells from individual embryos were processed to make cDNA libraries and served as biological replicates. We generated 184 libraries for our analysis from a total of 60 separate conditions (Table S1). To assess replicate correlation, we performed t-distributed stochastic neighbor embedding (t-SNE) analysis on our dataset, which revealed a high degree of cross-replicate clustering (Figure S2A). We also computed Spearman correlation coefficients between samples, which were typically higher than 0.8, confirming a high level of replicate correlation (Figure S2B).

Unsupervised hierarchical clustering of RNA-seq profiles revealed that early germ cells, late germ cells, and somatic (gonadal and non-gonadal) cells formed three distinct branches (59 of our 60 conditions), suggesting that our transcriptomic data recapitulated the ontology of germ cell development (Figure 1A). We further interrogated the data using multi-dimensional scaling (MDS), which once again highlighted the transcriptional distinction between these three groups. The first dimension separated somatic cells from germ cells, and developmental progression was noted along the second dimension, indicating progressive germ cell differentiation after PGC colonization of the gonad (Figure 1B). The single outlier condition in hierarchical clustering (E12.5 XX male) clearly segregated as germ cells during MDS. We assayed the expression of previously described pluripotency and meiotic genes (Seisenberger et al., 2012) in our germ cell populations. In both male and female germ cells, pluripotency genes were upregulated following the time of PGC specification at E7.25, and were subsequently repressed following sex determination (Figure 1C). Meiotic genes were upregulated in female germ cells from E12.5 but remained repressed at this time point in males (Figure 1D). Thus, our germ cell population faithfully recapitulated the orderly program of sex-specific changes that occur during germ cell development.

Upregulation of the Active X Chromosome in Non-gonadal and Gonadal Somatic Cells

Next, we ascertained whether X upregulation occurs in somatic cells. We analyzed X chromosome activity in XX and XY non-gonadal somatic cells (E14.5 liver and tail) and gonadal somatic cells (E9.5–E18.5). In both males and females, these cells carry one active X chromosome, as females undergo somatic X chromosome inactivation. In order to assay expression at

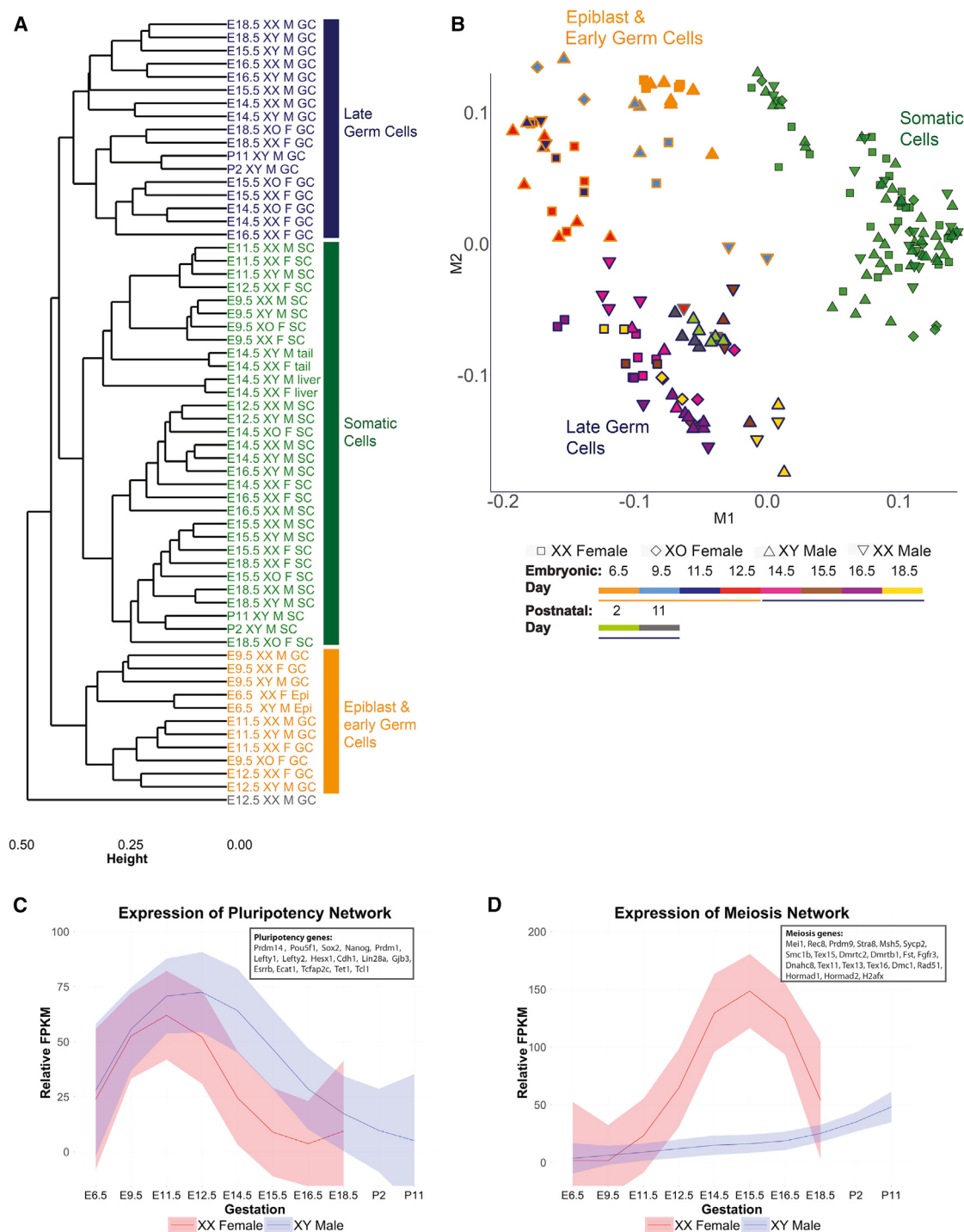

Figure 1. Transcriptome Profiling of Mouse Germ Cells

(A) Unsupervised hierarchical clustering of all dataset samples. The dendrogram shown is based on Jensen-Shannon distances between conditions. Dark orange, epiblast and early germ cells; dark blue, late germ cells; green, somatic cells; gray, E12.5 XX male germ cells.

(B) MDS plot of gene expression in all replicates within the dataset.

(C) Time course of relative expression (FPKM) for pluripotency genes in XY males and XX females.

(D) Time course of relative expression (FPKM) for meiosis genes in XY males and XX females.

a chromosome-wide level, we charted median X chromosome expression in relation to that of median expression from the autosomes as a comparison. This X:A ratio was calculated as the ratio of the respective medians, with 95% confidence intervals of the ratio computed using the bootstrap method, which involves random sampling from a distribution with replacement (Efron and Tibshirani, 1993).

Consistent with earlier studies (Julien et al., 2012; Xiong et al., 2010), when all genes with a fragments per kilobase of transcript per million reads (FPKM) ≥ 0 were included in our analysis, X:A ratios were low in non-gonadal and gonadal somatic tissues, implying that X upregulation does not occur (Figure 2A). However, the X chromosome is enriched relative to the autosomes in genes that are silent in somatic cells, and therefore including these genes artificially lowers X:A ratios (Deng et al., 2011; Yildirim et al., 2012). When we subsequently implemented increasing thresholds of expression ranging from FPKM 0.25 to 1, X:A ratios increased in both non-gonadal and gonadal somatic cells (Figure 2A). Hereafter, we implement an FPKM of ≥ 1 , which, based on other studies (Deng et al., 2011; Lin et al., 2011; Yildirim et al., 2012), is appropriate for assaying X upregulation (see Table S2 for numbers of genes exhibiting FPKM ≥ 1 for each sample).

While our data supported Ohno's prediction that expression from the single active X chromosome is upregulated in male and female somatic tissues (Ohno, 1967), two points warranted attention. Firstly, contrary to expectations, confidence intervals for X:A ratios in many of our somatic samples did not cross 1 when an FPKM of ≥ 1 was used (Figure 2A). Upregulation affects a subset of X chromosome genes with dosage-sensitive housekeeping functions (Birchler, 2012). Inclusion of expressed but non-dosage-compensated X genes dilutes X:A ratios to less than 1 (Pessia et al., 2012). We therefore refined our approach, employing a strategy (Ramskold et al., 2009) in which autosomal and X chromosome genes with housekeeping functions were identified by virtue of being ubiquitously expressed, i.e., exhibiting an FPKM ≥ 1 , throughout our entire RNA-seq sample dataset. To identify this gene set, we added samples in a stepwise manner, on each occasion retaining only genes that were expressed in all conditions. As expected with this approach, addition of successive samples initially resulted in a drop in the number of genes. However, the number subsequently stabilized at 5,656 autosomal and 155 X chromosomal genes (Figure 2B; Table S3). This ubiquitous gene set represented a sizable proportion of all expressed genes (FPKM ≥ 1), for example, 49.7% of autosomal and 41.2% of X-linked genes in E14.5 male liver. The number of ubiquitous genes did not change when liver, which expresses a lower number of genes compared with other tissues (Ramskold et al., 2009), was excluded. Gene ontology term enrichment analysis provided strong evidence that these genes had essential housekeeping roles, for example, in the cell cycle, RNA processing, and protein localization (Figure 2C). When we repeated our analysis focusing on these ubiquitous genes, X:A ratio confidence intervals in somatic tissues crossed 1 (Figure 2D). Our approach therefore successfully enriched for X genes that are subject to upregulation.

A second consideration was that the X chromosome is over-represented in genes expressed in reproductive tissues, including gonadal somatic cells and germ cells (Deng et al.,

2011; Khil et al., 2004; Mueller et al., 2008, 2013; Wang et al., 2001). Such genes have tissue-biased functions, and are therefore less likely to require dosage compensation. The inclusion of such highly expressed genes can skew X:A ratios to higher levels (Deng et al., 2011). Indeed, we noted that X:A ratios were higher in some of our gonadal somatic populations than in non-gonadal somatic ones (Figures 2A and 2D). To investigate this difference further, we conducted a pairwise analysis of gene expression ratios comparing E14.5 XY male gonadal somatic cells to E14.5 XY male liver (Figure 2E). There was a significant difference in the distribution of expression from the X chromosome compared with the autosomes, and a greater proportion of highly expressed genes on the X chromosome were expressed in gonadal somatic cells (Kolmogorov-Smirnov test: $p = 0.0004$; Figure 2E). We controlled for this effect by imposing an upper FPKM threshold set to exclude the top centile of gene expression (Figure 2F; see STAR Methods for details). A total of 4,188 autosomal and 102 X-linked loci remained from the original ubiquitous gene set upon imposing this threshold (Table S4). As expected, when we included this threshold, X:A ratios in gonadal somatic cell populations fell, now resembling those seen in non-gonadal somatic populations (Figure 2G). In light of these findings, we decided to analyze X dosage compensation patterns during germline development both in the presence and absence of an upper FPKM threshold. Finally, we applied our criteria to existing RNA-seq datasets from embryonic stem cells (ESCs) and epiblast-like cells (Hayashi et al., 2011; Lowe et al., 2015; von Meyenn et al., 2016). We observed X:A ratios crossing 1 in male ESCs and epiblast-like cells, and crossing 2 in female ESCs (Figure S3).

XX Female Germ Cells Exhibit Excess X Chromosome Dosage During Reprogramming

Next, we analyzed X dosage compensation patterns during and after reprogramming in the XX female germline. Previous work has shown that one of the two X chromosomes is already inactive in the epiblast prior to PGC specification (Hayashi et al., 2012; McMahon and Monk, 1983; Sugimoto and Abe, 2007), and subsequently reactivates during germline reprogramming (Chuva de Sousa Lopes et al., 2008; de Napoles et al., 2007; Sugimoto and Abe, 2007). However, the behavior of the active X chromosome during this period has not been examined.

X:A ratios approximated 1 in epiblast cells at E6.5, whether or not an upper FPKM threshold was used (Figures 3A and 3B). Given that, in these cells, one of the two X chromosomes is silenced, this finding shows that the active X chromosome is already upregulated prior to PGC specification (see next section for confirmation). X dosage compensation was thereafter retained during germ cell migration at E9.5 (Figures 3A and 3B). Interestingly, however, from E11.5, X:A ratios increased, exceeding 1, resulting in a state of X dosage excess (Figures 3A and 3B). The relative overexpression of X genes persisted for 3 days until E14.5, i.e., after gonadal colonization and entry into meiosis (Figures 3A and 3B). This period is noteworthy since it corresponds to the time during which genes on the inactive X chromosome gradually reactivate (Chuva de Sousa Lopes et al., 2008; de Napoles et al., 2007; Sugimoto and Abe, 2007). We reappraised the behavior of the inactive X chromosome from E6.5 to E14.5 using *Xist* RNA fluorescence in situ

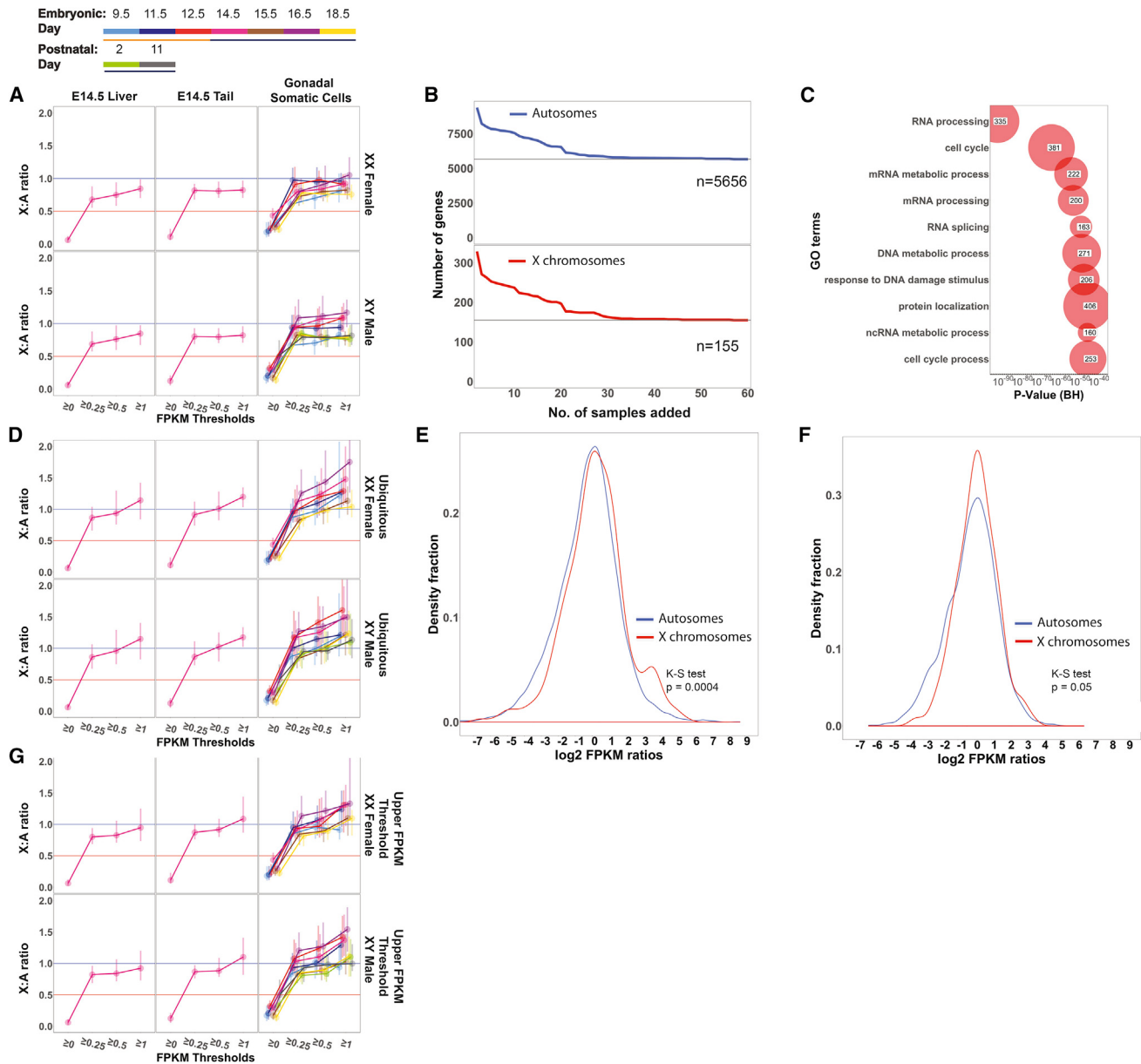

Figure 2. Analysis of X:A Ratios in Somatic Cells

(A) Bootstrapped X:A ratios from E14.5 liver and tail and gonadal somatic cell populations (E9.5–P11; E9.5 refers to caudal embryo somatic cells) using different lower FPKM thresholds, and focusing on all genes (i.e., “non-ubiquitous genes”).

(B) Definition of a ubiquitous gene set by addition of sequential samples of germ cells and somatic cells. The number of genes expressed in “all samples” is predictably high when the number of samples included is low and decreases as more samples are added before plateauing at a stable gene set.

(C) Gene ontology (GO) enrichment analysis, defined using Database for Annotation, Visualization and Integrated Discovery (DAVID), of ubiquitously expressed genes (5,656 on the autosomes and 155 on the X chromosome). Benjamini-Hochberg-corrected p values were plotted against the top ten “biological processes” GO terms.

(D) X:A ratios for the same samples as in (A), but considering only ubiquitous genes.

(E) Density plot of log₂ FPKM ratios of expression in E14.5 XY male gonadal somatic cells and E14.5 XY male liver for the X chromosome versus the autosomes. (F) Density plot of log₂ FPKM ratios of expression in E14.5 XY male gonadal somatic cells and E14.5 XY male liver for the X chromosome versus the autosomes, after imposition of an upper FPKM expression threshold.

(G) X:A ratios for the same samples as in (A) and (D) considering only ubiquitous genes together with an upper FPKM threshold.

hybridization (FISH) (Figure S4), and found that the major loss of *Xist* clouds occurred between E9.5 and E11.5, precisely coincident with the shift to X dosage excess as determined by X:A ratio calculations (Figure S4). Subsequently, X dosage compensation

was reinstated, with X:A ratios returning to 1 at E15.5 and remaining so thereafter (Figures 3A and 3B). Thus, XX female germ cells show X dosage compensation states that are dynamic and that differ from the soma.

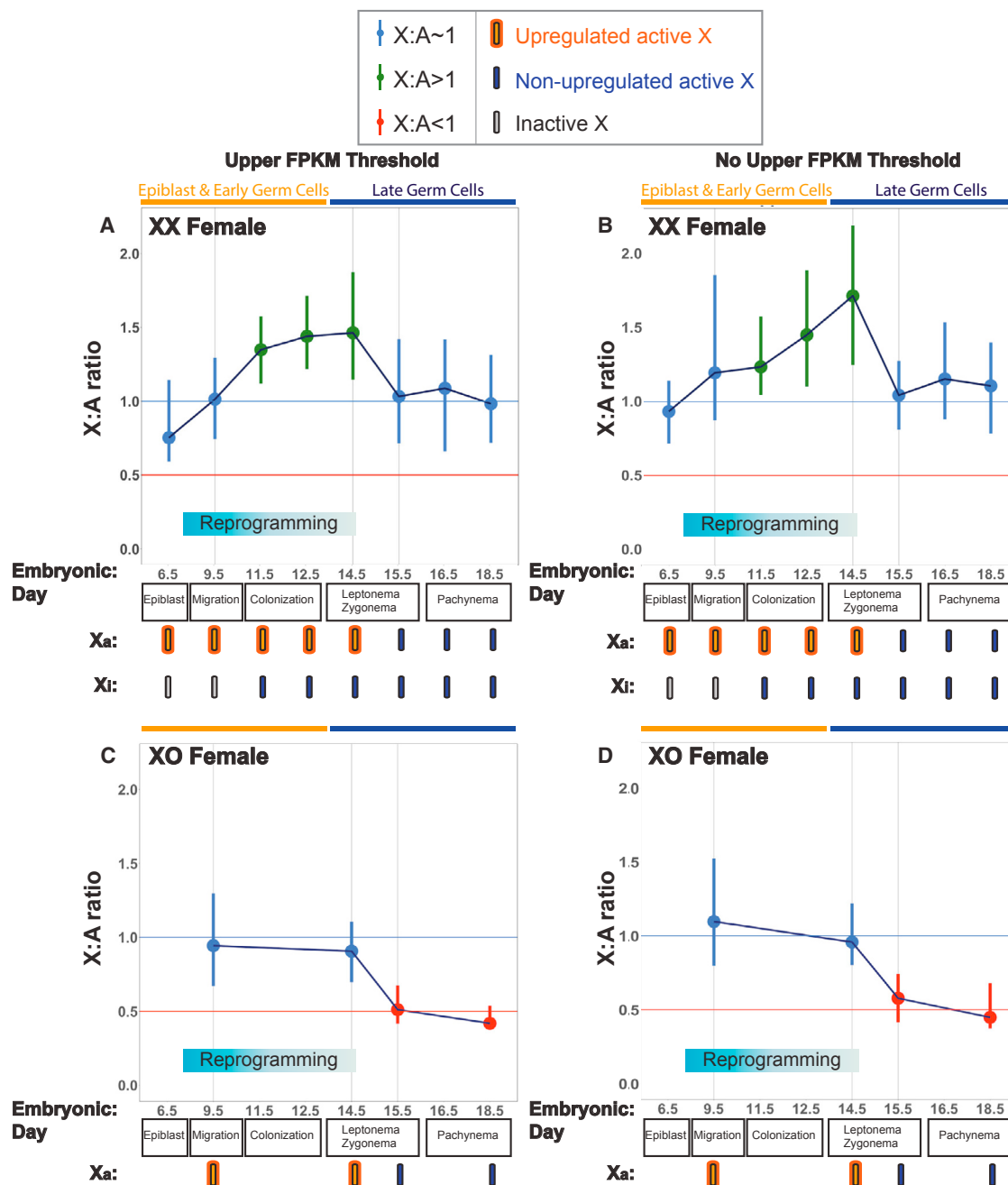

Figure 3. X:A Ratios in XX and XO Female Mouse Germ Cells

(A) X:A ratios of ubiquitously expressed genes in XX females using an FPKM ≥ 1 and an upper expression threshold. (B) X:A ratios of ubiquitously expressed genes in XX females using an FPKM ≥ 1 and no upper expression threshold. (C) X:A ratios of ubiquitously expressed genes in XO females using an FPKM ≥ 1 and an upper expression threshold. (D) X:A ratios of ubiquitously expressed genes in XO females using an FPKM ≥ 1 and no upper expression threshold. Dark orange, epiblast and early germ cells; dark blue, late germ cells.

XO Female Germ Cells Exhibit X Dosage Decompensation Following Reprogramming

Our findings demonstrated that during germline reprogramming in XX female germ cells, expression of X genes undergoes dynamic changes relative to those of the autosomes, resulting in a period of excess X chromosome dosage. How-

ever, we could not decipher the relative contribution of the two X chromosomes to this unusual X dosage compensation state. To better understand this phenomenon, we repeated our analysis using germ cells from Turner syndrome female (XO) mice, which carry one rather than two X chromosomes (Figures 3C and 3D). XO mice are viable (Burgoyne et al.,

1983a, 1983b), and therefore presumably achieve X dosage compensation through upregulation of their single active X chromosome. By assaying the behavior of this single active X chromosome in isolation, the cause of the X dosage excess observed in XX females can subsequently be deduced. We focused on four time points that in XX females represented states of initial X dosage compensation (E9.5), X dosage excess (E14.5), and reinstatement of X dosage balance (E15.5 and E18.5; [Figures 3A and 3B](#)). XO females are subfertile, but the timing of germ cell loss in these mice initiates later, at E19.5 ([Burgoyne and Baker, 1981, 1985](#)). Thus, our data were not confounded by germ cell elimination.

As observed in XX germ cells ([Figures 3A and 3B](#)), X:A ratios in XO germ cells at E9.5 approximated 1, confirming that XO PGCs achieve dosage compensation through upregulation of the single active X chromosome ([Figures 3C and 3D](#)). Notably, however, at E14.5, when X dosage excess was observed in XX germ cells ([Figures 3A and 3B](#)), XO germ cells retained X dosage balance ([Figures 3C and 3D](#)). A pairwise comparison revealed that the distribution of X chromosome expression was significantly different between XO and XX females at this age (Wilcoxon test, $p = 8 \times 10^{-4}$). Thus, in the female germline, upregulation of the active X chromosome is maintained at E14.5. By deduction, the state of X dosage excess in XX females at E14.5 ([Figures 3A and 3B](#)) results from the additive effects of persistent upregulation of the active X chromosome, and reactivation of the previously inactive X chromosome.

Interestingly, dosage compensation patterns in XO female germ cells changed markedly between E14.5 and E15.5. At E15.5, when XX germ cells regained X:A balance, XO germ cells became X dosage decompensated, i.e., the X:A ratio fell below 1 ([Figures 3C and 3D](#)). Thus, upregulation of the active X chromosome is lost during a 24 hr period in early meiosis. Subsequently, at E18.5, X:A ratios decreased further ([Figures 3C and 3D](#)). At this stage, XO germ cells have entered pachynema, when meiotic silencing begins to inactivate genes on the single unsynapsed X chromosome ([Baarends et al., 2005; Turner et al., 2005](#)). Meiotic silencing affects only 50% of XO oocytes and occurs in an inefficient manner ([Cloutier et al., 2015a, 2015b](#)), potentially explaining why X:A ratios did not decrease more dramatically. We conclude that germline reprogramming in females is associated not only with reactivation of the inactive X chromosome but also with loss of upregulation of the active X chromosome, and that the completion of these events by E15.5 permits reinstatement of X dosage balance in E15.5 XX female germ cells. In addition, XX and XO females exhibit markedly different dosage compensation patterns.

XY Male Germ Cells Exhibit X Dosage Decompensation Following Reprogramming

Our findings showed that in the female germline, upregulation of the active X chromosome is lost following reprogramming, restoring X:A balance in XX females but causing X dosage decompensation in XO females. XY males, like XO females, have a single X chromosome. We therefore asked whether X dosage decompensation was also observed in the male germline. To address this, we tracked dosage compensation patterns throughout male germ cell development at equivalent time points to those analyzed in XX females.

As noted in XX and XO females, XY epiblast cells exhibited X dosage compensation at E6.5, and X:A ratios subsequently remained at 1 from E9.5 and E14.5 ([Figures 4A and 4B](#)). However, at later time points, X:A ratios in XY male germ cells fell, with upper confidence intervals no longer crossing 1. This phenomenon was observed from E15.5 when an upper FPKM threshold was imposed and from E18.5 when no upper threshold was imposed. In both scenarios, the X dosage decompensated state persisted postnatally in spermatogonia and even in leptotene/zygotene spermatocytes. At E15.5, the distribution of X chromosome expression differed significantly between XY males and XO females (Wilcoxon test, $p = 0.0041$), suggesting that the extent of decompensation in XY male germ cells was not as pronounced as in XO females. However, at E15.5, male germ cells are in mitosis, while female germ cells are in leptotene/zygonema. When we compared stage-matched XY male (P11) and XO female (E15.5) leptotene/zygotene cells, the X chromosome expression distributions did not differ (Wilcoxon test, $p = 0.2362$). We conclude that XY males exhibit X dosage decompensation after reprogramming, and that XO females exhibit a “sex-reversed” X dosage compensation pattern, i.e., one that is reminiscent of XY males rather than XX females.

XX Male Germ Cells Exhibit Excess X Chromosome Dosage During Reprogramming

Our analysis demonstrated that X dosage compensation patterns in XO females resemble those in XY males. We next asked the converse question, namely whether dosage compensation patterns in XX males are similar to those in XX females. We calculated X:A ratios in germ cells derived from Klinefelter syndrome variant (XX) males. These mice have two X chromosomes, and thus undergo X chromosome inactivation, but they are male due to presence of a sex reversing Sry transgene ([Mahadevaiah et al., 1998](#)). XX males exhibited dosage balance at E9.5, E11.5 and E12.5 ([Figures 4C and 4D](#)). However, as observed in XX females ([Figures 3A and 3B](#)), at E14.5 X:A ratios in XX males exceeded 1 ([Figures 4C and 4D](#)), and the distribution of X chromosome expression at this stage differed significantly from that in XY males (Wilcoxon test: $p = 9 \times 10^{-4}$). Germ cell loss is first evident in Klinefelter male mice from E15.5 ([Hunt et al., 1998](#)); we therefore avoided drawing conclusions about X:A ratios at this stage and after. Our findings show that, like XX females, XX males exhibit excess X chromosome dosage during reprogramming.

Dosage Decompensation in XY Male Germ Cells is not Corrected by Expression of Autosomal Retrogenes

A number of X genes, including some within our dataset, e.g., *Pgk1*, *Pdha1*, have duplicate copies known as retrogenes. These arise by reverse transcription of X-derived RNAs and subsequent integration at autosomal sites, and thus differ from their parental copies in being intronless ([Kaessmann et al., 2009; Khil et al., 2005; Wang, 2004](#)). Retrogenes show testis-biased expression, and some encode proteins with similar functions to their X-linked progenitors ([Bradley et al., 2004; Danshina et al., 2010; Emerson et al., 2004; Rohozinski and Bishop, 2004; Wang and Page, 2002](#)). We wondered if X-derived retrogenes were expressed after germline reprogramming and, if so, whether their expression could compensate for the drop in X chromosome output that we

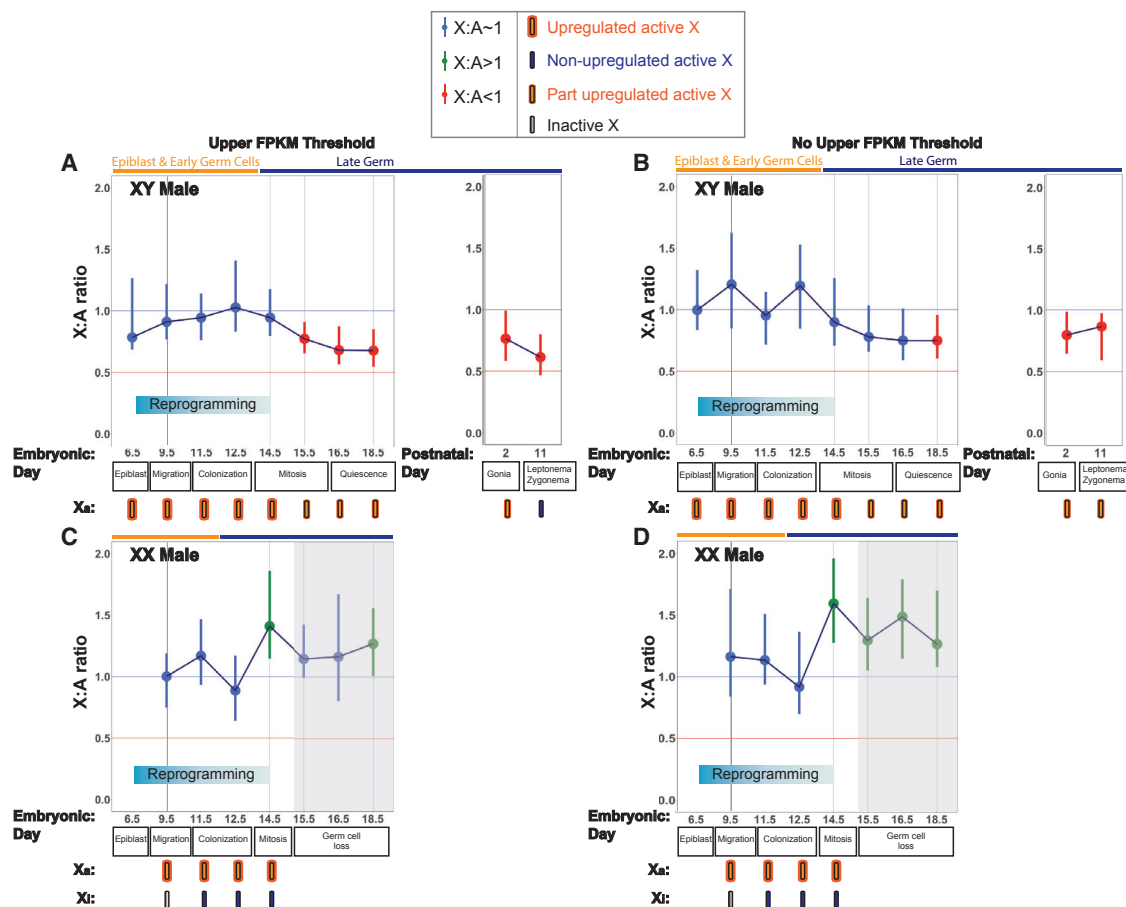

Figure 4. X:A Ratios in XY and XX Sry Male Mouse Germ Cells

(A) X:A ratios of ubiquitously expressed genes in XY males using an FPKM ≥ 1 and an upper expression threshold.
 (B) X:A ratios of ubiquitously expressed genes in XY males using an FPKM ≥ 1 and no upper expression threshold.
 (C) X:A ratios of ubiquitously expressed genes in XX Sry males using an FPKM ≥ 1 and an upper expression threshold.
 (D) X:A ratios of ubiquitously expressed genes in XX Sry males using an FPKM ≥ 1 and no upper expression threshold.
 Dark orange, epiblast and early germ cells; dark blue, late germ cells.

observed in XY males during this period. Using the criteria that they should be intronless, putatively protein coding, autosomally encoded, and share $>80\%$ nucleotide identity with their X-parental copies, we identified retrogenes for 14 of our 155 ubiquitous X genes (Figure S5A). These retrogenes were expressed at very low levels relative to their X counterparts from E6.5–P11 (Figure S5A). This finding was consistent with previous work showing that X-derived retrogenes initiate expression later, during, or after pachynema (Kaessmann et al., 2009; Khil et al., 2005; Wang, 2004). The X dosage decompensated state in XY male germ cells was not altered when we combined regene-derived RNA-seq FPKMs with those from their parental X-encoded progenitors at each stage of germ cell (Figure S5B). We conclude that retrogenes do not compensate for the decrease in expression of their X-linked progenitors.

Conserved Dosage Compensation Patterns During Human Germline Development

We next turned our attention to X chromosome activity during human germline reprogramming. We analyzed existing

RNA-seq datasets that comprised human gonadal somatic cells and XY ESCs, as well as germ cells purified using FACS for c-KIT alone (Gkoutela et al., 2015) or both c-KIT and TNAP (Tang et al., 2015). The data encompassed a broad timeline of human PGC development including early germ cells, when germline reprogramming occurs (weeks 5.5, 7, and 9) and later germ cells, when sexually dimorphic development takes place (Figure S1). Where available, biological replicates were pooled as indicated (Tang et al., 2015). We used this entire sample set to generate a set of ubiquitously expressed (FPKM ≥ 1) genes, in a manner similar to our approach in mice. We identified 8,226 autosomal and 259 X-linked ubiquitously expressed genes (Table S5), which fell to 7,719 autosomal and 236 X-linked genes when an upper FPKM threshold was imposed (Table S6). We observed X:A ratios of 1 for these genes in XY ESCs and gonadal somatic cells, in the presence or absence of an upper FPKM threshold (Figure 5A), indicating that X upregulation occurs in these populations. Notably, however, germ cells exhibited dosage compensation patterns that differed from the soma, and

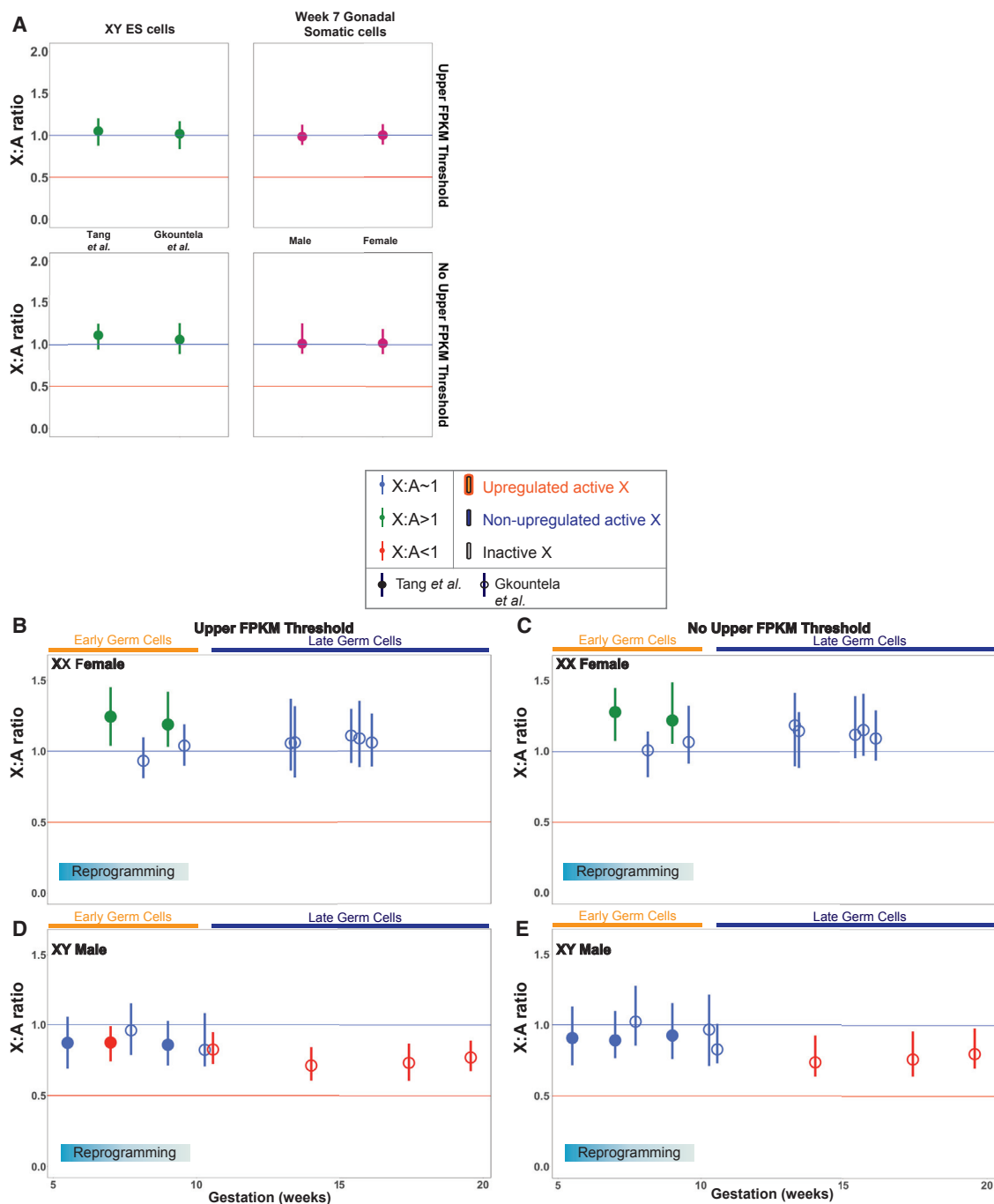

Figure 5. X:A Ratios in Human XY ESCs, Gonadal Somatic Cells, and Germ Cells

(A) X:A ratios of ubiquitously expressed genes in XY ESCs, and week 7 male gonadal somatic cells and female gonadal somatic cells using an FPKM ≥ 1 and the presence or absence of an upper FPKM threshold.

(B) X:A ratios of ubiquitously expressed genes in XX female germ cells using an FPKM ≥ 1 and an upper expression threshold. Data from Tang *et al.* (2015) are shown with closed circles; data from Gkoutela *et al.* (2015) are shown with open circles.

(C) X:A ratios of ubiquitously expressed genes in XX female germ cells using an FPKM ≥ 1 and no upper expression threshold.

(D) X:A ratios of ubiquitously expressed genes in XY male germ cells using an FPKM ≥ 1 and an upper expression threshold.

(E) X:A ratios of ubiquitously expressed genes in XY male germ cells using an FPKM ≥ 1 and no upper expression threshold.

Dark orange, early germ cells; dark blue, late germ cells.

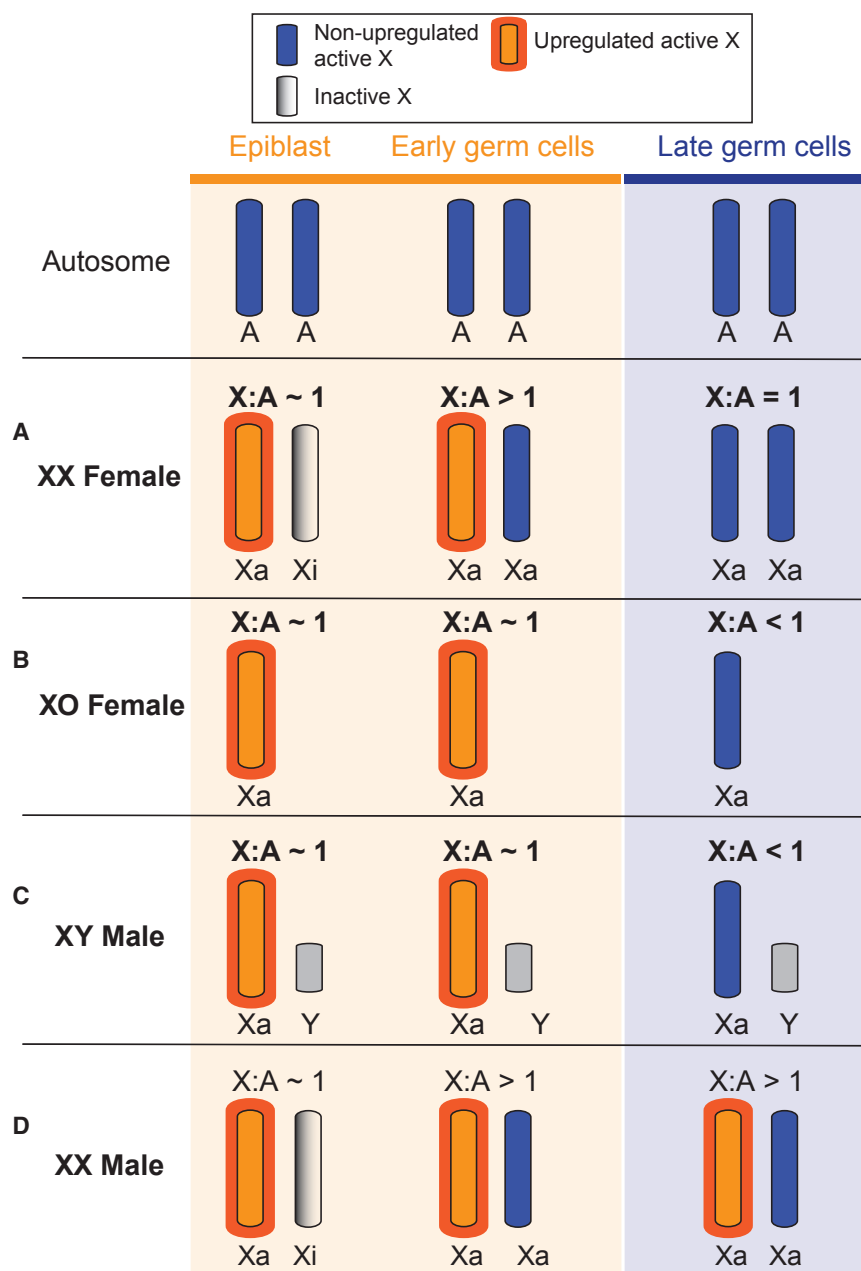

Figure 6. A Model for X Dosage Compensation Patterns

A model for X dosage compensation patterns in the XX Female (A), XO female (B), XY male (C), and XX male (D) mammalian germline. Prior to reprogramming, XX female and XX male germ cells have one upregulated X chromosome (Xa, orange) and one inactive X chromosome (Xi, gray), while XY males and XO female cells have a single upregulated X chromosome (Xa, orange). In all four cases, the dosage of X genes is balanced with that of the autosomes (A, blue), i.e., the X:A ratio is 1. During reprogramming, upregulation of the active X chromosome is maintained in all four genotypes, but in XX females and XX males, the inactive X chromosome begins to reactivate (gray becomes blue). The outcome is X:A ratios greater than 1 in XX females and in XX males. Later, X upregulation is lost, reinstating X:A ratios in XX females to 1, but leaving XY males and XO females X dosage decompensated, with X:A ratios less than 1. XX males show germ cell loss from E15.5, which may be due to high X:A ratios seen.

DISCUSSION

Here, we have tracked X dosage compensation patterns in mouse and human germ cells from their inception through to their later development. We uncover unusual X dosage compensation states that differ between the germline and the soma and between males and females, and that are conserved in humans (Figure 6).

During their specification and migration, female and male germ cells exhibit X:A ratios of 1, showing that upregulation of the active X chromosome is present in both sexes during early germline development (Figures 6A–6D). Subsequently, however, X dosage compensation patterns become sexually dimorphic. During reprogramming in XX female mice, X:A ratios exceed 1, generating a state of X dosage excess that persists for 3 days from E12.5 to E14.5 (Figure 6A). We propose that the excess X chromosome output arises from reactivation of the inactive X chromosome, combined with ongoing upregulation of the active X chromosome. While supported by analysis of XO females, it is also possible that this excess X dosage in XX females results from a modest increase in expression from both X chromosomes. This alternative hypothesis could be tested in future by allele-specific RNA-seq. Upregulation of the active X chromosome is subsequently reversed between E14.5 and E15.5. In XX females, this corrects the hyperactive X state (Figure 6A), but in XO females it leads to X dosage decompensation (Figure 6B). The decompensated state in XO females is independent of meiotic silencing, which occurs later, at E16.5–E18.5 (Cloutier et al., 2015b). Our findings, together with previous work (Fukuda et al., 2015), show

were reminiscent of those seen in mouse germ cells. During germline reprogramming (weeks 7–9), when reactivation of the inactive X chromosome takes place (Tang et al., 2015), XX female samples derived from Tang et al. (2015) exhibited X dosage excess, both in the presence or absence of an upper FPKM threshold (Figures 5B and 5C). This phenomenon was not observed in males (Figures 5D and 5E). Furthermore, after germline reprogramming, while X:A ratios in females returned to 1 (Figures 5B and 5C), those in males fell below 1, resulting in an X dosage decompensated state (Figures 5D and 5E). We conclude that differences in X dosage compensation states exhibited between somatic and germ cells, and between females and males, are conserved in mice and humans.

we propose that the excess X chromosome output arises from reactivation of the inactive X chromosome, combined with ongoing upregulation of the active X chromosome. While supported by analysis of XO females, it is also possible that this excess X dosage in XX females results from a modest increase in expression from both X chromosomes. This alternative hypothesis could be tested in future by allele-specific RNA-seq. Upregulation of the active X chromosome is subsequently reversed between E14.5 and E15.5. In XX females, this corrects the hyperactive X state (Figure 6A), but in XO females it leads to X dosage decompensation (Figure 6B). The decompensated state in XO females is independent of meiotic silencing, which occurs later, at E16.5–E18.5 (Cloutier et al., 2015b). Our findings, together with previous work (Fukuda et al., 2015), show

that X upregulation no longer occurs in XO and XX females after reprogramming.

As observed in female germ cells, output from the active X chromosome in XY male germ cells falls following reprogramming, resulting in X:A ratios below 1 (Figure 6C). At leptoneuma/zygoneuma, the extent of decompensation in XY males is similar to that of XO females. Nevertheless, reversal of X upregulation in XY males clearly occurs over a more protracted period (i.e., days) than in XO females (hours). Sexual dimorphisms are regulated by X dosage or X imprinting effects, or by the presence or absence of the Y chromosome (Arnold et al., 2012). Given that XO females and XY males both have a single X chromosome of maternal origin, differences between these models must be due to the Y chromosome. The Y chromosome could influence X dosage compensation states as a result of *Sry* determining male gonad development (i.e., phenotypic sex), or/and the continued effects of other Y-encoded genes that could modulate X dosage compensation states. It is already well established that the X chromosome is silenced during pachynema by the process of meiotic sex chromosome inactivation (MSCI) (Ichijima et al., 2012; Inagaki et al., 2010; Turner, 2007; Yan and McCarrey, 2009), but its activity before this time has not been thoroughly investigated. Our current findings show that expression from the X chromosome in fact undergoes a stepwise decrease during male germline development: prior to reprogramming, the X chromosome is fully upregulated, at leptoneuma/zygoneuma upregulation is lost, and subsequently, during pachynema, it is fully silenced by MSCI.

Finally, our studies are informative with respect to understanding the etiology of infertility in sex chromosome aneuploidies. XO females exhibit X chromosome decompensation reminiscent of that seen in wild-type males, while XX males exhibit X dosage excess like that in wild-type females. We suggest that this sex-reversed X dosage compensation pattern could contribute to the infertility phenotypes. For example, while it is accepted that infertility in XX males is due to reactivation of the inactive X chromosome (Hall et al., 2006; Mroz et al., 1999), one model attributes this specifically to a double dose of genes showing spermatogonial-specific expression (De Jonge and Barratt, 2006). Our data present an additional possibility, in which germ cell loss in XX males results from a mismatch between germline sex and X dosage compensation state. Previous work advocates a predominant role for dosage-sensitive X genes in sex chromosome aneuploid phenotypes (Pessia et al., 2012). In either scenario, preventing X chromosome reactivation or promoting loss of the excess X chromosome may prove of therapeutic benefit in Klinefelter syndrome infertility.

STAR★METHODS

Detailed methods are provided in the online version of this paper and include the following:

- KEY RESOURCES TABLE
- CONTACT FOR REAGENT AND RESOURCE SHARING
- EXPERIMENTAL MODEL SUBJECT DETAILS
  - Mice
- METHOD DETAILS
  - Isolation of Cells for RNA-Seq Analysis
  - Fluorescence-Activated Cell Sorting (FACS)

- Isolation of RNA and Construction of cDNA Libraries
- RNA FISH
- QUANTIFICATION, STATISTICAL ANALYSIS AND ADDITIONAL RESOURCES
  - Bioinformatic Analyses
  - Retrogene Analysis
- DATA AND SOFTWARE AVAILABILITY
  - Software
  - Data Resources

SUPPLEMENTAL INFORMATION

Supplemental Information includes five figures and six tables and can be found with this article online at <http://dx.doi.org/10.1016/j.devcel.2016.12.023>.

AUTHOR CONTRIBUTIONS

Conceptualization: M.S. and J.T.; Methodology: M.S. and J.T.; Investigation: M.S., S.K.M., and J.T.; Validation: H.R., M.B.S., and A.H.F.M.P.; Writing: M.S. and J.T.; Reviewing and Editing: M.S. and J.T.; Funding Acquisition: J.T. and M.S.; Resources: O.O., L.B., and A.S.; Supervision: J.T.

ACKNOWLEDGMENTS

We are grateful to Wolf Reik and Azim Surani for kindly providing the *Oct4*-EGFP mouse strain. We are grateful to members of the Turner and Niakan laboratories and to Ian Henderson for critical reading of the manuscript, to Fanny Decarpentrie for generating Figure 6, the Crick FACS STP for cell purifications and the Crick Histology STP for the preparation of cryosections. We are also grateful to Christine Disteche for initial advice on study design, and Donald Bell for initial advice with programming in R. This work was supported by the Francis Crick Institute, which receives its core funding from Cancer Research UK, the UK Medical Research Council (U117588498), and the Wellcome Trust, as well as the NIHR Clinical Lectureship (M.S.) and Academy of Medical Sciences grant (AMS-SGCL5, M.S.).

Received: April 6, 2016

Revised: November 1, 2016

Accepted: December 27, 2016

Published: January 26, 2017

REFERENCES

- Adler, D.A., Rugarli, E.I., Lingenfelter, P.A., Tsuchiya, K., Poslinski, D., Liggitt, H.D., Chapman, V.M., Elliott, R.W., Ballabio, A., and Disteche, C.M. (1997). Evidence of evolutionary up-regulation of the single active X chromosome in mammals based on *Cic4* expression levels in *Mus spretus* and *Mus musculus*. *Proc. Natl. Acad. Sci. USA* 94, 9244–9248.
- Arnold, A.P., Chen, X., and Itoh, Y. (2012). What a difference an X or Y makes: sex chromosomes, gene dose, and epigenetics in sexual differentiation. *Handb. Exp. Pharmacol.* 67–88.
- Baarends, W.M., Wassenaar, E., van der Laan, R., Hoogerbrugge, J., Sleddens-Linkels, E., Hoijmakers, J.H., de Boer, P., and Grootegoed, J.A. (2005). Silencing of unpaired chromatin and histone H2A ubiquitination in mammalian meiosis. *Mol. Cell Biol.* 25, 1041–1053.
- Bachtrog, D. (2013). Y-chromosome evolution: emerging insights into processes of Y-chromosome degeneration. *Nat. Rev. Genet.* 14, 113–124.
- Birchler, J.A. (2012). Claims and counterclaims of X-chromosome compensation. *Nat. Struct. Mol. Biol.* 19, 3–5.
- Bradley, J., Baltus, A., Skaletsky, H., Royce-Tolland, M., Dewar, K., and Page, D.C. (2004). An X-to-autosome retrogene is required for spermatogenesis in mice. *Nat. Genet.* 36, 872–876.
- Burgoyne, P.S. (1998). The mammalian Y chromosome: a new perspective. *Bioessays* 20, 363–366.

- Burgoyne, P.S., and Baker, T.G. (1981). Oocyte depletion in XO mice and their XX sibs from 12 to 200 days post partum. *J. Reprod. Fertil.* **61**, 207–212.
- Burgoyne, P.S., and Baker, T.G. (1985). Perinatal oocyte loss in XO mice and its implications for the aetiology of gonadal dysgenesis in XO women. *J. Reprod. Fertil.* **75**, 633–645.
- Burgoyne, P.S., Evans, E.P., and Holland, K. (1983a). XO monosomy is associated with reduced birthweight and lowered weight gain in the mouse. *J. Reprod. Fertil.* **68**, 381–385.
- Burgoyne, P.S., Tam, P.P., and Evans, E.P. (1983b). Retarded development of XO conceptuses during early pregnancy in the mouse. *J. Reprod. Fertil.* **68**, 387–393.
- Charlesworth, B. (1996). The evolution of chromosomal sex determination and dosage compensation. *Curr. Biol.* **6**, 149–162.
- Charlesworth, B., and Charlesworth, D. (2000). The degeneration of Y chromosomes. *Philos. Trans. R. Soc. Lond. B Biol. Sci.* **355**, 1563–1572.
- Chuva de Sousa Lopes, S.M., Hayashi, K., Shovlin, T.C., Mifsud, W., Surani, M.A., and McLaren, A. (2008). X chromosome activity in mouse XX primordial germ cells. *PLoS Genet.* **4**, e30.
- Cloutier, J.M., Mahadevaiah, S.K., Ellnati, E., Nussenzweig, A., Toth, A., and Turner, J.M. (2015a). Histone H2AFX links meiotic chromosome asynapsis to prophase I oocyte loss in mammals. *PLoS Genet.* **11**, e1005462.
- Cloutier, J.M., Mahadevaiah, S.K., Ellnati, E., Toth, A., and Turner, J. (2015b). Mammalian meiotic silencing exhibits sexually dimorphic features. *Chromosoma* **125**, 215–226.
- Conrad, T., and Akhtar, A. (2011). Dosage compensation in *Drosophila melanogaster*: epigenetic fine-tuning of chromosome-wide transcription. *Nat. Rev. Genet.* **13**, 123–134.
- Cortez, D., Marin, R., Toledo-Flores, D., Froidevaux, L., Liechti, A., Waters, P.D., Grutzner, F., and Kaessmann, H. (2014). Origins and functional evolution of Y chromosomes across mammals. *Nature* **508**, 488–493.
- Danshina, P.V., Geyer, C.B., Dai, Q., Goulding, E.H., Willis, W.D., Kitto, G.B., McCarrey, J.R., Eddy, E.M., and O'Brien, D.A. (2010). Phosphoglycerate kinase 2 (PGK2) is essential for sperm function and male fertility in mice. *Biol. Reprod.* **82**, 136–145.
- De Jonge, C.J., and Barratt, C. (2006). *The Sperm Cell: Production, Maturation, Fertilization, Regeneration* (Cambridge University Press).
- de Napoles, M., Nesterova, T., and Brockdorff, N. (2007). Early loss of Xist RNA expression and inactive X chromosome associated chromatin modification in developing primordial germ cells. *PLoS One* **2**, e860.
- Deng, X., Hiatt, J.B., Nguyen, D.K., Ercan, S., Sturgill, D., Hillier, L.W., Schlesinger, F., Davis, C.A., Reinke, V.J., Gingeras, T.R., et al. (2011). Evidence for compensatory upregulation of expressed X-linked genes in mammals, *Caenorhabditis elegans* and *Drosophila melanogaster*. *Nat. Genet.* **43**, 1179–1185.
- Deng, X., Berletch, J.B., Ma, W., Nguyen, D.K., Hiatt, J.B., Noble, W.S., Shendure, J., and Distech, C.M. (2013). Mammalian X upregulation is associated with enhanced transcription initiation, RNA half-life, and MOF-mediated H4K16 acetylation. *Dev. Cell* **25**, 55–68.
- Deng, X., Berletch, J.B., Nguyen, D.K., and Distech, C.M. (2014). X chromosome regulation: diverse patterns in development, tissues and disease. *Nat. Rev. Genet.* **15**, 367–378.
- Dupont, C., and Gribnau, J. (2013). Different flavors of X-chromosome inactivation in mammals. *Curr. Opin. Cell Biol.* **25**, 314–321.
- Efron, B., and Tibshirani, R. (1993). *An Introduction to the Bootstrap* (Chapman & Hall).
- Emerson, J.J., Kaessmann, H., Betran, E., and Long, M. (2004). Extensive gene traffic on the mammalian X chromosome. *Science* **303**, 537–540.
- Faucillion, M.L., and Larsson, J. (2015). Increased expression of X-linked genes in mammals is associated with a higher stability of transcripts and an increased ribosome density. *Genome Biol. Evol.* **7**, 1039–1052.
- Fukuda, A., Tanino, M., Matoba, R., Umezawa, A., and Akutsu, H. (2015). Imbalance between the expression dosages of X-chromosome and autosomal genes in mammalian oocytes. *Sci. Rep.* **5**, 14101.
- Gelbart, M.E., and Kuroda, M.I. (2009). *Drosophila* dosage compensation: a complex voyage to the X chromosome. *Development* **136**, 1399–1410.
- Gendrel, A.V., and Heard, E. (2014). Noncoding RNAs and epigenetic mechanisms during X-chromosome inactivation. *Annu. Rev. Cell Dev. Biol.* **30**, 561–580.
- Gkoutela, S., Zhang, K.X., Shafiq, T.A., Liao, W.W., Hargan-Calvopina, J., Chen, P.Y., and Clark, A.T. (2015). DNA demethylation dynamics in the human prenatal germline. *Cell* **161**, 1425–1436.
- Guo, F., Yan, L., Guo, H., Li, L., Hu, B., Zhao, Y., Yong, J., Hu, Y., Wang, X., Wei, Y., et al. (2015). The transcriptome and DNA methylome landscapes of human primordial germ cells. *Cell* **161**, 1437–1452.
- Gupta, V., Parisi, M., Sturgill, D., Nuttall, R., Doctolero, M., Dudko, O.K., Malley, J.D., Eastman, P.S., and Oliver, B. (2006). Global analysis of X-chromosome dosage compensation. *J. Biol.* **5**, 3.
- Hall, H., Hunt, P., and Hassold, T. (2006). Meiosis and sex chromosome aneuploidy: how meiotic errors cause aneuploidy; how aneuploidy causes meiotic errors. *Curr. Opin. Genet. Dev.* **16**, 323–329.
- Hayashi, K., Ohta, H., Kurimoto, K., Aramaki, S., and Saitou, M. (2011). Reconstitution of the mouse germ cell specification pathway in culture by pluripotent stem cells. *Cell* **146**, 519–532.
- Hayashi, K., Ogushi, S., Kurimoto, K., Shimamoto, S., Ohta, H., and Saitou, M. (2012). Offspring from oocytes derived from in vitro primordial germ cell-like cells in mice. *Science* **338**, 971–975.
- Hilscher, W. (1974). Kinetics of prespermatogenesis and spermatogenesis. *Verh. Anat. Ges.* **68**, 39–62, [in German].
- Hughes, J.F., and Page, D.C. (2015). The biology and evolution of mammalian Y chromosomes. *Annu. Rev. Genet.* **49**, 507–527.
- Hunt, P.A., Worthman, C., Levinson, H., Stallings, J., LeMaire, R., Mroz, K., Park, C., and Handel, M.A. (1998). Germ cell loss in the XXY male mouse: altered X-chromosome dosage affects prenatal development. *Mol. Reprod. Dev.* **49**, 101–111.
- Ichijima, Y., Sin, H.S., and Namekawa, S.H. (2012). Sex chromosome inactivation in germ cells: emerging roles of DNA damage response pathways. *Cell Mol. Life Sci.* **69**, 2559–2572.
- Inagaki, A., Schoenmakers, S., and Baarends, W.M. (2010). DNA double strand break repair, chromosome synapsis and transcriptional silencing in meiosis. *Epigenetics* **5**, 255–266.
- Julien, P., Brawand, D., Soumillon, M., Necseulea, A., Liechti, A., Schutz, F., Daish, T., Grutzner, F., and Kaessmann, H. (2012). Mechanisms and evolutionary patterns of mammalian and avian dosage compensation. *PLoS Biol.* **10**, e1001328.
- Kaessmann, H., Vinckenbosch, N., and Long, M. (2009). RNA-based gene duplication: mechanistic and evolutionary insights. *Nat. Rev. Genet.* **10**, 19–31.
- Khil, P.P., Smirnova, N.A., Romanienko, P.J., and Camerini-Otero, R.D. (2004). The mouse X chromosome is enriched for sex-biased genes not subject to selection by meiotic sex chromosome inactivation. *Nat. Genet.* **36**, 642–646.
- Khil, P.P., Oliver, B., and Camerini-Otero, R.D. (2005). X for intersection: retrotransposition both on and off the X chromosome is more frequent. *Trends Genet.* **21**, 3–7.
- Kim, D., Pertea, G., Trapnell, C., Pimentel, H., Kelley, R., and Salzberg, S.L. (2013). TopHat2: accurate alignment of transcriptomes in the presence of insertions, deletions and gene fusions. *Genome Biol.* **14**, R36.
- Leitch, H.G., Tang, W.W., and Surani, M.A. (2013). Primordial germ-cell development and epigenetic reprogramming in mammals. *Curr. Top. Dev. Biol.* **104**, 149–187.
- Lin, H., Gupta, V., Vermilyea, M.D., Falciani, F., Lee, J.T., O'Neill, L.P., and Turner, B.M. (2007). Dosage compensation in the mouse balances up-regulation and silencing of X-linked genes. *PLoS Biol.* **5**, e326.
- Lin, H., Halsall, J.A., Antczak, P., O'Neill, L.P., Falciani, F., and Turner, B.M. (2011). Relative overexpression of X-linked genes in mouse embryonic stem cells is consistent with Ohno's hypothesis. *Nat. Genet.* **43**, 1169–1170, author reply 1171–1162.

- Lowe, R., Gemma, C., Rakyan, V.K., and Holland, M.L. (2015). Sexually dimorphic gene expression emerges with embryonic genome activation and is dynamic throughout development. *BMC Genomics* 16, 295.
- Mahadevaiah, S.K., Odorisio, T., Elliott, D.J., Rattigan, A., Szot, M., Laval, S.H., Washburn, L.L., McCarrey, J.R., Cattanch, B.M., Lovell-Badge, R., et al. (1998). Mouse homologues of the human AZF candidate gene RBM are expressed in spermatogonia and spermatids, and map to a Y chromosome deletion interval associated with a high incidence of sperm abnormalities. *Hum. Mol. Genet.* 7, 715–727.
- Mahadevaiah, S.K., Costa, Y., and Turner, J.M. (2009). Using RNA FISH to study gene expression during mammalian meiosis. *Methods Mol. Biol.* 558, 433–444.
- McMahon, A., and Monk, M. (1983). X-chromosome activity in female mouse embryos heterozygous for Pgk-1 and Searle's translocation, T(X; 16) 16H. *Genet. Res.* 41, 69–83.
- Mroz, K., Carrel, L., and Hunt, P.A. (1999). Germ cell development in the XXY mouse: evidence that X chromosome reactivation is independent of sexual differentiation. *Dev. Biol.* 207, 229–238.
- Mueller, J.L., Mahadevaiah, S.K., Park, P.J., Warburton, P.E., Page, D.C., and Turner, J.M. (2008). The mouse X chromosome is enriched for multicopy testis genes showing postmeiotic expression. *Nat. Genet.* 40, 794–799.
- Mueller, J.L., Skaletsky, H., Brown, L.G., Zaghul, S., Rock, S., Graves, T., Auger, K., Warren, W.C., Wilson, R.K., and Page, D.C. (2013). Independent specialization of the human and mouse X chromosomes for the male germ line. *Nat. Genet.* 45, 1083–1087.
- Muller, H.J. (1914). A factor for the fourth chromosome of *Drosophila*. *Science* 39, 906.
- Muzumdar, M.D., Tasic, B., Miyamichi, K., Li, L., and Luo, L. (2007). A global double-fluorescent Cre reporter mouse. *Genesis* 45, 593–605.
- Nguyen, D.K., and Distech, C.M. (2006). Dosage compensation of the active X chromosome in mammals. *Nat. Genet.* 38, 47–53.
- Ohno, S. (1967). *Sex Chromosomes and Sex-Linked Genes* (Springer-Verlag).
- Pessia, E., Makino, T., Bailly-Bechet, M., McLysaght, A., and Marais, G.A. (2012). Mammalian X chromosome inactivation evolved as a dosage-compensation mechanism for dosage-sensitive genes on the X chromosome. *Proc. Natl. Acad. Sci. USA* 109, 5346–5351.
- Pessia, E., Engelstadter, J., and Marais, G.A. (2014). The evolution of X chromosome inactivation in mammals: the demise of Ohno's hypothesis? *Cell Mol. Life Sci.* 71, 1383–1394.
- Picelli, S., Faridani, O.R., Bjorklund, A.K., Winberg, G., Sagasser, S., and Sandberg, R. (2014). Full-length RNA-seq from single cells using Smart-seq2. *Nat. Protoc.* 9, 171–181.
- Ramskold, D., Wang, E.T., Burge, C.B., and Sandberg, R. (2009). An abundance of ubiquitously expressed genes revealed by tissue transcriptome sequence data. *PLoS Comput. Biol.* 5, e1000598.
- Rohozinski, J., and Bishop, C.E. (2004). The mouse juvenile spermatogonial depletion (jsd) phenotype is due to a mutation in the X-derived retrogene, mUtp14b. *Proc. Natl. Acad. Sci. USA* 101, 11695–11700.
- Sadate-Ngatchou, P.I., Payne, C.J., Dearth, A.T., and Braun, R.E. (2008). Cre recombinase activity specific to postnatal, premeiotic male germ cells in transgenic mice. *Genesis* 46, 738–742.
- Saitou, M. (2009). Germ cell specification in mice. *Curr. Opin. Genet. Dev.* 19, 386–395.
- Seisenberger, S., Andrews, S., Krueger, F., Arand, J., Walter, J., Santos, F., Popp, C., Thienpont, B., Dean, W., and Reik, W. (2012). The dynamics of genome-wide DNA methylation reprogramming in mouse primordial germ cells. *Mol. Cell* 48, 849–862.
- Straub, T., and Becker, P.B. (2007). Dosage compensation: the beginning and end of generalization. *Nat. Rev. Genet.* 8, 47–57.
- Sugimoto, M., and Abe, K. (2007). X chromosome reactivation initiates in nascent primordial germ cells in mice. *PLoS Genet.* 3, e116.
- Tang, W.W., Dietmann, S., Irie, N., Leitch, H.G., Floros, V.I., Bradshaw, C.R., Hackett, J.A., Chinnery, P.F., and Surani, M.A. (2015). A unique gene regulatory network resets the human germline epigenome for development. *Cell* 161, 1453–1467.
- Trapnell, C., Roberts, A., Goff, L., Pertea, G., Kim, D., Kelley, D.R., Pimentel, H., Salzberg, S.L., Rinn, J.L., and Pachter, L. (2012). Differential gene and transcript expression analysis of RNA-seq experiments with TopHat and Cufflinks. *Nat. Protoc.* 7, 562–578.
- Trapnell, C., Hendrickson, D.G., Sauvageau, M., Goff, L., Rinn, J.L., and Pachter, L. (2013). Differential analysis of gene regulation at transcript resolution with RNA-seq. *Nat. Biotechnol.* 31, 46–53.
- Turner, J.M. (2007). Meiotic sex chromosome inactivation. *Development* 134, 1823–1831.
- Turner, J.M., Mahadevaiah, S.K., Fernandez-Capetillo, O., Nussenzweig, A., Xu, X., Deng, C.X., and Burgoyne, P.S. (2005). Silencing of unsynapsed meiotic chromosomes in the mouse. *Nat. Genet.* 37, 41–47.
- Vergouwen, R.P., Jacobs, S.G., Huiskamp, R., Davids, J.A., and de Rooij, D.G. (1991). Proliferative activity of gonocytes, Sertoli cells and interstitial cells during testicular development in mice. *J. Reprod. Fertil.* 93, 233–243.
- von Meyenn, F., Berrens, R.V., Andrews, S., Santos, F., Collier, A.J., Krueger, F., Osorno, R., Dean, W., Rugg-Gunn, P.J., and Reik, W. (2016). Comparative principles of DNA methylation reprogramming during human and mouse in vitro primordial germ cell specification. *Dev. Cell* 39, 104–115.
- Wang, P.J. (2004). X chromosomes, retrogenes and their role in male reproduction. *Trends Endocrinol. Metab.* 15, 79–83.
- Wang, P.J., and Page, D.C. (2002). Functional substitution for TAF(II)250 by a retroposed homolog that is expressed in human spermatogenesis. *Hum. Mol. Genet.* 11, 2341–2346.
- Wang, P.J., McCarrey, J.R., Yang, F., and Page, D.C. (2001). An abundance of X-linked genes expressed in spermatogonia. *Nat. Genet.* 27, 422–426.
- Xiong, Y., Chen, X., Chen, Z., Wang, X., Shi, S., Wang, X., Zhang, J., and He, X. (2010). RNA sequencing shows no dosage compensation of the active X-chromosome. *Nat. Genet.* 42, 1043–1047.
- Yan, W., and McCarrey, J.R. (2009). Sex chromosome inactivation in the male. *Epigenetics* 4, 452–456.
- Yildirim, E., Sadreyev, R.I., Pinter, S.F., and Lee, J.T. (2012). X-chromosome hyperactivation in mammals via nonlinear relationships between chromatin states and transcription. *Nat. Struct. Mol. Biol.* 19, 56–61.
- Yin, S., Deng, W., Zheng, H., Zhang, Z., Hu, L., and Kong, X. (2009). Evidence that the nonsense-mediated mRNA decay pathway participates in X chromosome dosage compensation in mammals. *Biochem. Biophys. Res. Commun.* 383, 378–382.
- Yoshimizu, T., Sugiyama, N., De Felice, M., Yeom, Y.I., Ohbo, K., Masuko, K., Obinata, M., Abe, K., Scholer, H.R., and Matsui, Y. (1999). Germline-specific expression of the Oct-4/green fluorescent protein (GFP) transgene in mice. *Dev. Growth Differ.* 41, 675–684.

STAR★METHODS

KEY RESOURCES TABLE

Reagent or Resource	Source	Identifier
Critical Commercial Assays		
Ultralow input RNA-Seq Kit (SMARTER)	Clontech	Cat No #634935
Deposited Data		
Our raw and analyzed RNA-seq dataset	This paper	European Nucleotide Archive (EMBL-EBI) E-MTAB-4616
Experimental Models: Organisms/Strains		
Mouse: Oct-4 EGFP	Wolf Reik/Azim Surani, Cambridge, UK	-na-
Mouse: ROSA26-540 Tomato-EGFP strain (ROSA ^{mT/mG})	The Jackson Laboratory	Gt(ROSA)26Sortm4(ACTB-tdTomato,-EGFP)Luo
Mouse: XY- +Sry (B6)	Francis Crick Institute	-na-
Mouse: X ^Y O male	Francis Crick Institute, Burgoyne, 1998	-na
Sequence-Based Reagents		
Xist RNA FISH probes	Mahadevaiah et al., 2009	fosmid probe: W11-2363-H9
Software and Algorithms		
TopHat v2.0.13	Genome Biology 2013 14:R36	http://www.ccb.jhu.edu/software/tophat/index.shtml
Cufflinks v2.2.1	Trapnell et al., 2012	http://cole-trapnell-lab.github.io/cufflinks/
R v.3.2.0	R Core Team (2016)	https://www.R-project.org/
cummeRbund v2.7.2 (R package)	L. Goff, C. Trapnell, D. Kelley	http://compbio.mit.edu/cummeRbund/
pairwiseCI (R package)	Frank Schaarschmidt [aut, cre], Daniel Gerhard [aut]	https://cran.r-project.org/
Rtsne	Jesse Krijthe [aut, cre], Laurens van der Maaten [cph] (Author of original C++ code)	https://cran.r-project.org/
Other		
Smart-Seq2 protocol	Picelli et al., 2014	NA
Mouse genome and annotation	ftp://igenome:G3nom3s4u@ussd-ftp.illumina.com/Mus_musculus/UCSC/mm10/Mus_musculus_UCSC_mm10.tar.gz	mm10
Publicly available data ES-cell RNA-seq datasets accessed	European Nucleotide Archive (EMBL-EBI)	SRR1448385, SRR1448386, SRR1448387, SRR1448388, SRR4241903, SRR4241904, SRR4241905, SRR4241906, SRR4241907, SRR4241908

CONTACT FOR REAGENT AND RESOURCE SHARING

Further information and requests for reagents may be directed to, and will be fulfilled by the Lead Contact, Dr. James Turner, james.turner@crick.ac.uk.

EXPERIMENTAL MODEL SUBJECT DETAILS

Mice

Oct4-EGFP mice were obtained from the Reik lab (Babraham, Cambridge, UK) and maintained on a B6 background. The reporter strain was used to isolate fluorescently marked germ cells through FACs. Homozygous XY⁻ +Sry (C57B6) Oct4-EGFP males were crossed with XX Oct4-EGFP females to yield XX female, XY male and XX male embryos used in the study.

XO embryos were generated by crossing an MF1 X^YO male ([Burgoyne, 1998](#)) to an XX Oct4-EGFP female. Timed matings were undertaken, and noon on the date of the vaginal plug was taken to be E0.5. Embryos were subsequently harvested on the stipulated day.

Leptotene/zygotene stage male germ cells were isolated at postnatal Day 11, by crossing a *Stra8-cre* line with ROSA26-Tomato-EGFP strain (ROSA^{mT/mG} strain; Mouse strain #007676 from The Jackson Laboratory), which enabled the timely isolation of these germ cells during the first-wave of male meiosis through FACS.

Samples were sexed as female (XX or XO) or male (XY or XX *Sry* transgenic) by a combination of gonadal inspection, conventional PCR genotyping and the presence of Y chromosome-derived transcripts from RNA-Seq data. Each condition comprised of at least two replicates. All animal procedures were in accordance with the United Kingdom Animal Scientific Procedures Act 1986 and were subject to local ethical review.

METHOD DETAILS

Isolation of Cells for RNA-Seq Analysis

Cell populations derived and purified from a single embryo at any stated age constituted a biological replicate to be used in subsequent steps. Germ cell and somatic populations were isolated at different stages, ranging from E9.5, E11.5, E12.5, E14.5, E15.5, E16.5, E18.5, P2, and P11. RNA was purified from individual E6.5 epiblasts.

Fluorescence-Activated Cell Sorting (FACS)

Germ cells (GFP-positive) and associated somatic cells (GFP-negative) were isolated separately from individual embryos using FACS sorting on the MoFlo XDP or FACS Aria platforms. Live cells, i.e. only those staining negative for propidium iodide, were collected, and typically purity checking on the GFP negative populations was >99%.

Isolation of RNA and Construction of cDNA Libraries

RNA was isolated from FACS sorted purified cell populations using the Ambion RNA isolation kit (Ambion #AM1931). Eluted RNA was used to obtain double-stranded-cDNA using the Clontech (SMARTer) Ultralow input RNA-Seq Kit according to the manufacturer's protocol, or the Smart-Seq2 protocol (Picelli et al., 2014). cDNA was normalized to 10 ng in low-TE buffer and was sheared using Covaris S-series, and was cleaned up with Zymo DNA conc-5 and eluted in 12 ul low-TE buffer. Libraries of ~300 bp were generated using Nugen Ovation Ultralow DR (part No. 0330) with 15 PCR cycles. Paired-end sequencing of 50 cycles was performed on the Illumina HiSeq2500 sequencer in house.

RNA FISH

Sample embryos were collected and fixed in 4% paraformaldehyde overnight at 4°C. Samples were then placed in 30% sucrose solution overnight. These were then placed in Optimal Cutting Temperature (OCT) compound and transferred to appropriate molds, quick-frozen and then stored at -80°C until the time of cryosectioning. 5 µm cryosections were collected and placed on cover-glasses, and subsequently processed with *Xist* RNA FISH probes using an established protocol (Mahadevaiah et al., 2009). Germ cells at E9.5, E11.5 and E14.5 were identified by the detection of expression of the *Oct4*-EGFP reporter transgene and nuclear morphology as visualised with DAPI DNA staining.

QUANTIFICATION, STATISTICAL ANALYSIS AND ADDITIONAL RESOURCES

Bioinformatic Analyses

Reads were aligned to the mouse genome (mm10) using Tophat2 v2.0.13. Transcript abundances were calculated using Cufflinks2 and Cuffdiff (Kim et al., 2013; Trapnell et al., 2012, 2013). At a minimum we had included two biological replicates for each condition that was analyzed (see Table S1). As recommended, annotated rRNA, mitochondrial transcripts and other very highly abundant transcripts were ignored from the analysis. Calculated transcript abundances in FPKM (Fragments per Kilobase per Million reads) were subsequently used in further analyses in R. Data visualization, hierarchical clustering and MDS were performed using the *cummeRbund* package. X:A ratios with 95% confidence intervals were calculated using the *pairwiseCI* package in R using 'Median.ratio' with 1,000,000 bootstrap replications.

We imposed an upper FPKM threshold that corresponded to the lowest 99th centile FPKM value of expression across conditions. ES Cell RNA seq datasets were accessed from the European Nucleotide Archive database.

Relevant samples from Study accession numbers PRJNA342888 and PRJNA253304 were accessed (Run accession numbers used are as stated in Figure S3) (Lowe et al., 2015; von Meyenn et al., 2016). These samples were aligned using Hisat2 -v2.0.5 to the mouse genome (mm10). Expression values (FPKM) and X:A ratios were calculated as stated.

Retrotransposon Analysis

Annotated retrotransposons were retrieved from the UCSC Genome Bioinformatics database from the mm10 mouse genome using Table Browser function and 18,456 records were obtained. X-linked genes from our ubiquitously expressed set of genes (Table S2) were cross-referred to this dataset. 14 out of 155 ubiquitous X-genes had autosomal retrotransposon counterparts which were intronless, putatively protein-coding and shared >80% nucleotide identity with their X-parental copies.

DATA AND SOFTWARE AVAILABILITY

Software

All software programs used in this study were from publicly available resources. Please refer to [Key Resources Table](#) for more details.

Data Resources

The dataset has been submitted via ArrayExpress to the European Nucleotide Archive (EMBL-EBI) and is publicly available under accession number ArrayExpress: E-MTAB-4616

Developmental Cell, Volume 40

Supplemental Information

Non-Canonical and Sexually Dimorphic X Dosage

Compensation States in the Mouse and Human Germline

Mahesh N. Sangrithi, Helene Royo, Shantha K. Mahadevaiah, Obah Ojarikre, Leena Bhaw, Abdul Sesay, Antoine H.F.M. Peters, Michael Stadler, and James M.A. Turner

INVENTORY OF SUPPLEMENTARY MATERIALS

Supplementary Figure 1 (Summarizes Method Details in the STAR Methods section).

Supplementary Figure 2 (Relates to Figure 1).

Supplementary Figure 3 (Relates to Figure 2; also see STAR methods).

Supplementary Figure 4 (Relates to Figure 3).

Supplementary Figure 5. Relative expression (FPKM) of X genes and their autosomal retrogene partners in XY male germ cells (Related to Figure 4).

Supplementary Table 1. Metrics of RNA-seq libraries used in this study (Related to Figure 1; refer to STAR methods (*Bioinformatic analysis*)).

Supplementary Table 2. Numbers and percentages of non-ubiquitous X and autosomal genes exhibiting FPKM ≥ 1 for samples shown in Figure 2A.

Supplementary Table 3. Ubiquitously expressed mouse genes - no upper FPKM threshold (Related to Figures 2D, 3B, 3D, 4B and 4D).

Supplementary Table 4. Ubiquitously expressed mouse genes - upper FPKM threshold (Related to Figures 2G, 3A, 3C, 4A and 4C).

Supplementary Table 5. Ubiquitously expressed human genes - no upper FPKM threshold (Related to Figures 5C and 5E).

Supplementary Table 6. Ubiquitously expressed human genes - upper FPKM threshold (Related to Figures 5B and 5D).

Supplementary Figure Legends:

Supplementary Figure 1 (Summarizes Method Details in the STAR Methods section).

Cartoon showing comparative time line of germ cell development and X chromosome activity status in mice and humans. Timing of expression of reporters used for mouse germ cell purification are also shown.

Supplementary Figure 2 (Relates to Figure 1).

A) Two dimensional tSNE dimensionality reduction analysis of all samples in the dataset. tSNE was performed using the top 500 genes showing the highest variance across the samples. B) Matrix of replicate correlations. Spearmann correlations were calculated for biological replicates used in this study, and were used to plot the matrix shown.

Supplementary Figure 3 (Relates to Figure 2; also see STAR methods).

X:A ratios in mouse ES and epiblast-like stem cells. SRA accession number of the datasets are stated in the figure. Male cells are underscored by a dark-blue bar and female ones by a pink bar.

Supplementary Figure 4 (Relates to Figure 3).

X-chromosome activity charted in individual female epiblast cells and germ cells using *Xist* RNA FISH analysis. A). A cartoon depicting the X chromosome activity in female E6.5 epiblast, germ cells from E9.5, E11.5 and E14.5 embryos. B). (i) Epiblast cells (elongated white arrows), (ii) E9.5 migratory primordial germ cells (white arrows), E11.5 gonadal germ cells (white arrows) and E14.5 oogonia (white arrows). Asterisks denote somatic cells. C). Stacked bar-chart showing percentage of cells in each sample that were positive (blue) or negative (red) for *Xist* RNA FISH signals. A low percentage of female germ cells at E11.5 and E14.5 exhibited *Xist* RNA FISH

signals. This finding confirmed that these cells had reactivated their second X-chromosome. Numbers of cells counted in of the samples are stated at the top of each bar.

Supplementary Figure 5. Relative expression (FPKM) of X genes and their autosomal retrogene partners in XY male germ cells (Related to Figure 4).

Supplementary Table 1. Metrics of RNA-seq libraries used in this study (Related to Figure 1; refer to STAR methods (*Bioinformatic analysis*)).

Supplementary Table 2. Numbers and percentages of non-ubiquitous X and autosomal genes exhibiting FPKM ≥ 1 for samples shown in Figure 2A.

Supplementary Table 3. Ubiquitously expressed mouse genes - no upper FPKM threshold (Related to Figures 2D, 3B, 3D, 4B and 4D).

Supplementary Table 4. Ubiquitously expressed mouse genes - upper FPKM threshold (Related to Figures 2G, 3A, 3C, 4A and 4C).

Supplementary Table 5. Ubiquitously expressed human genes - no upper FPKM threshold (Related to Figures 5C and 5E).

Supplementary Table 6. Ubiquitously expressed human genes - upper FPKM threshold (Related to Figures 5B and 5D).

Supplementary Figure 1

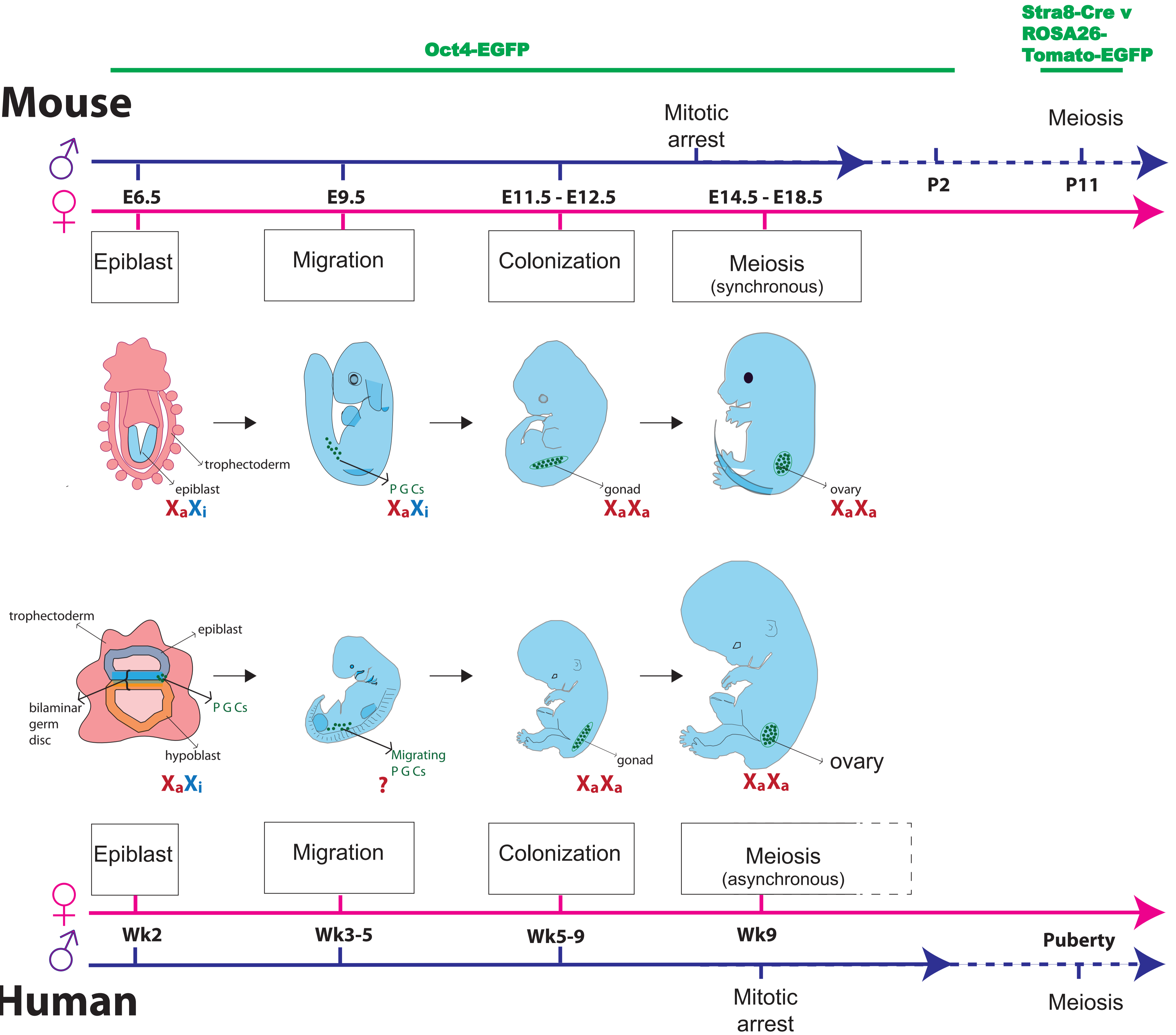

Supplementary Figure 2

A

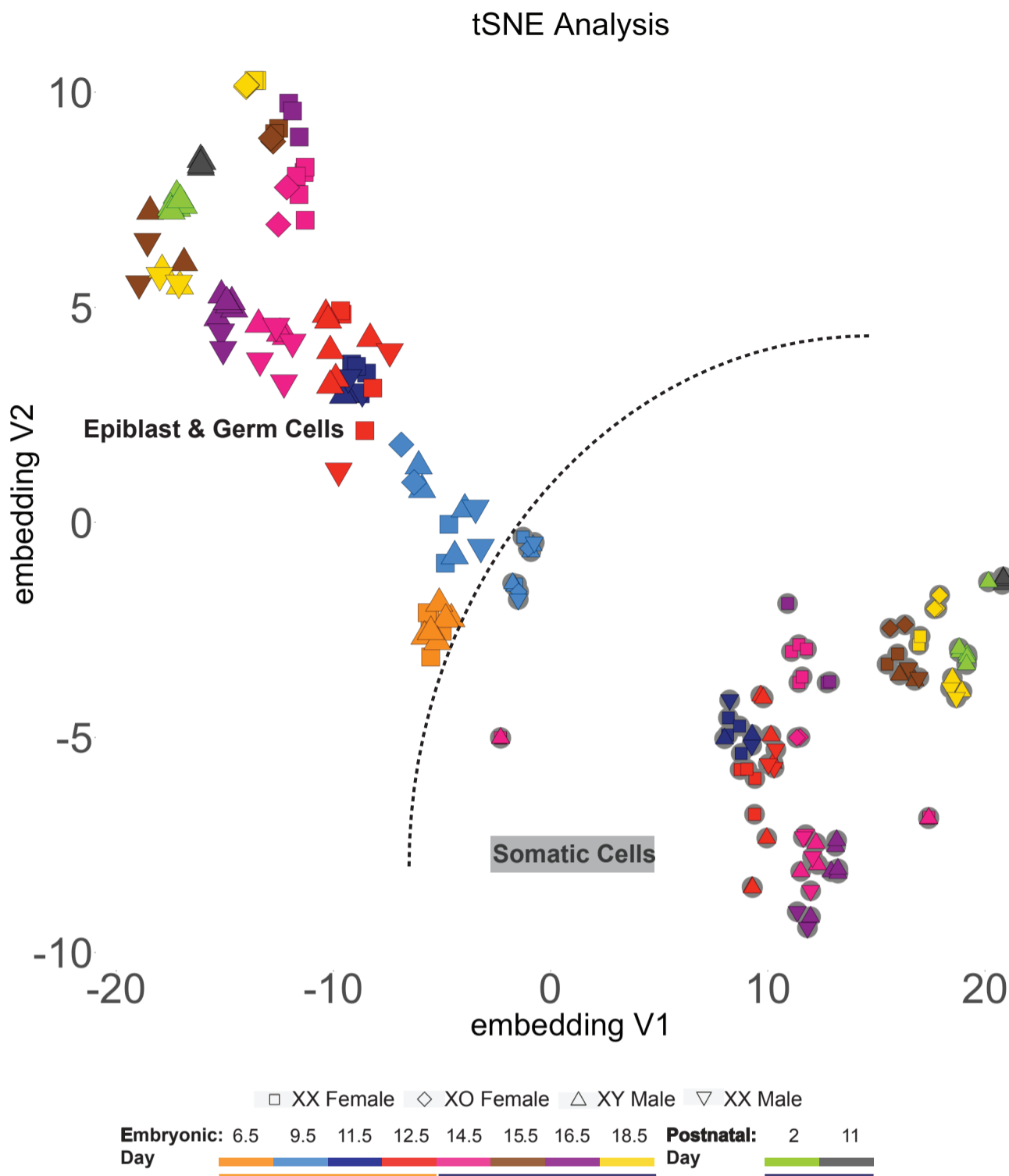

B

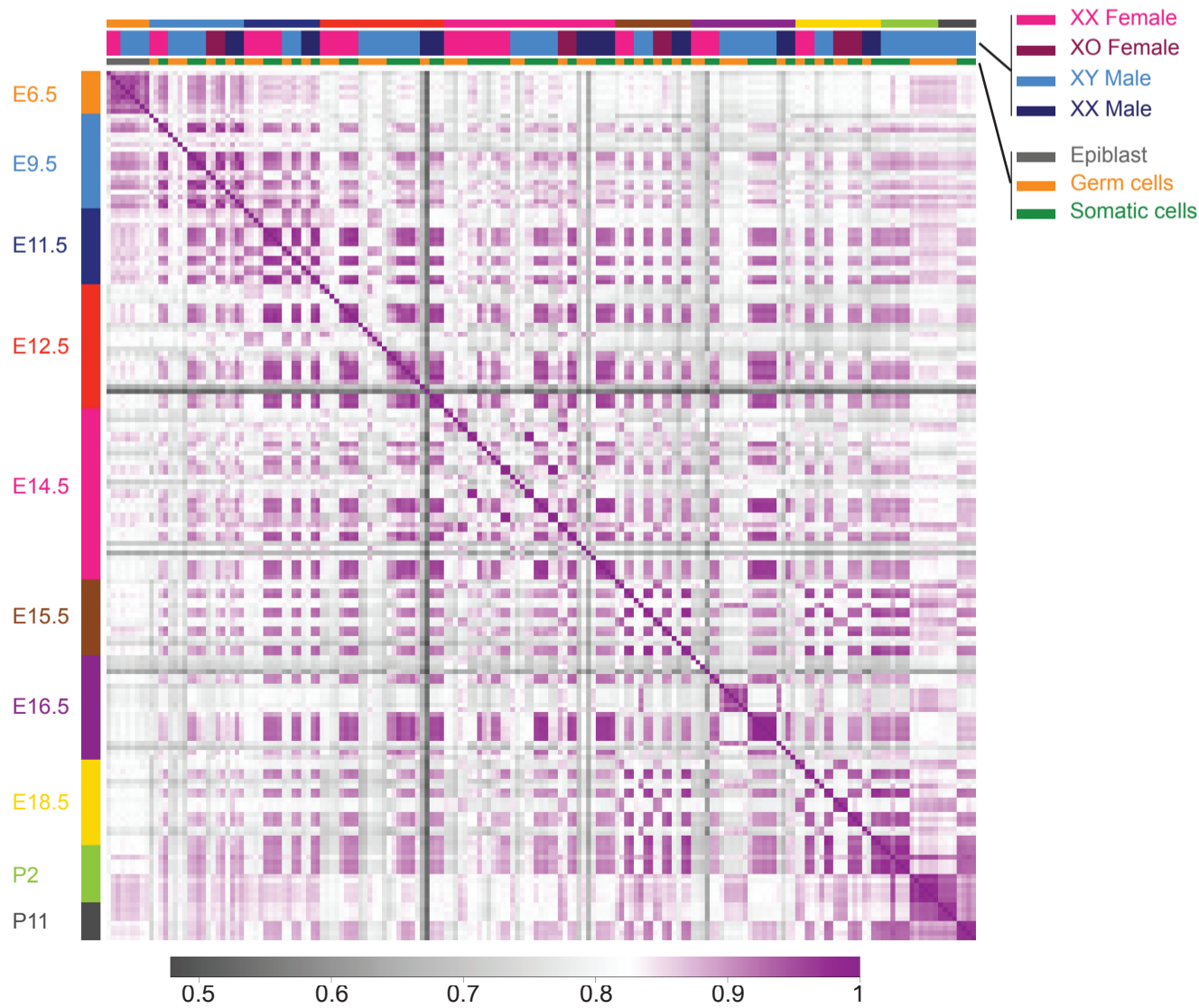

Supplementary Figure 3

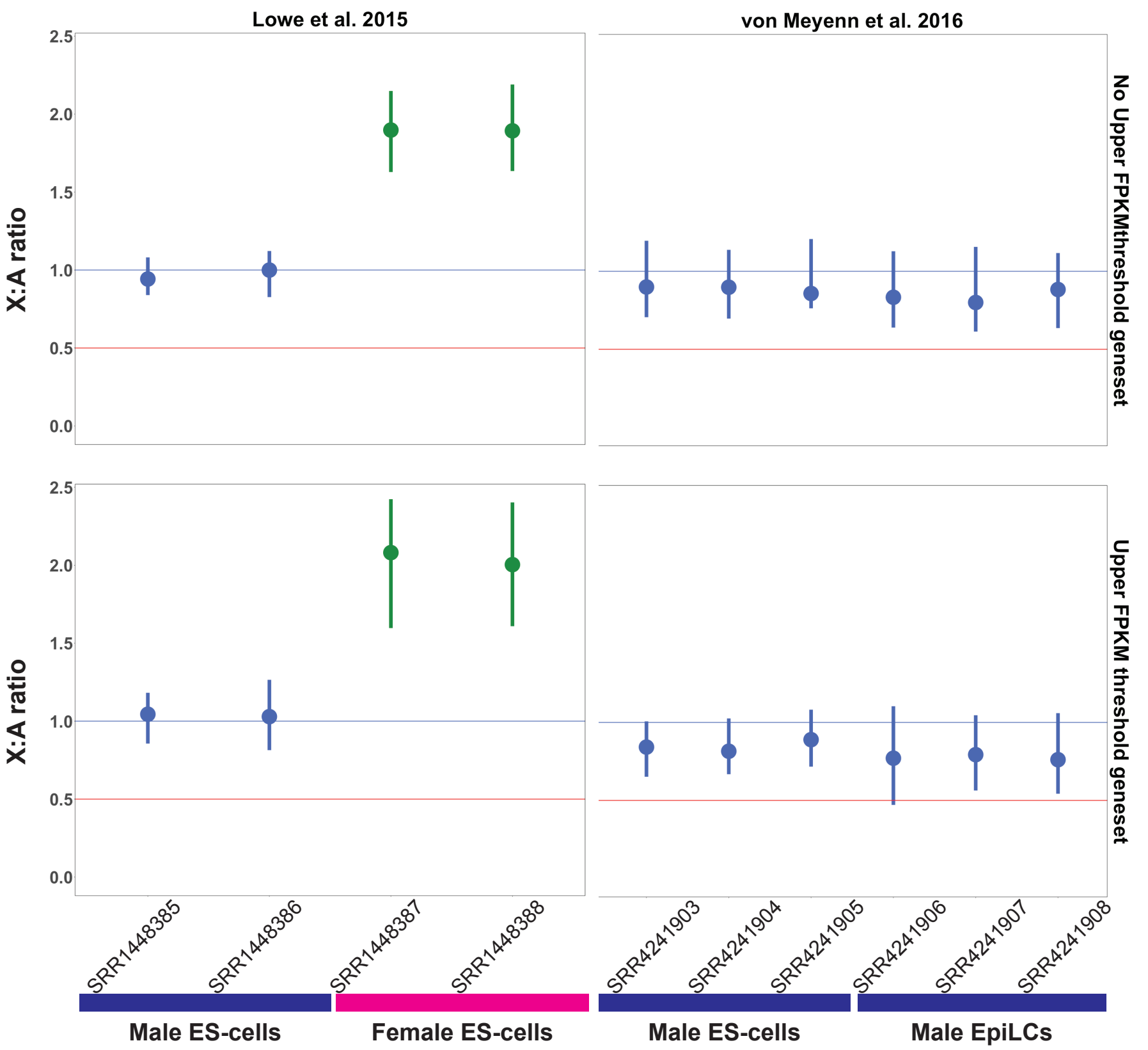

Supplementary Figure 4

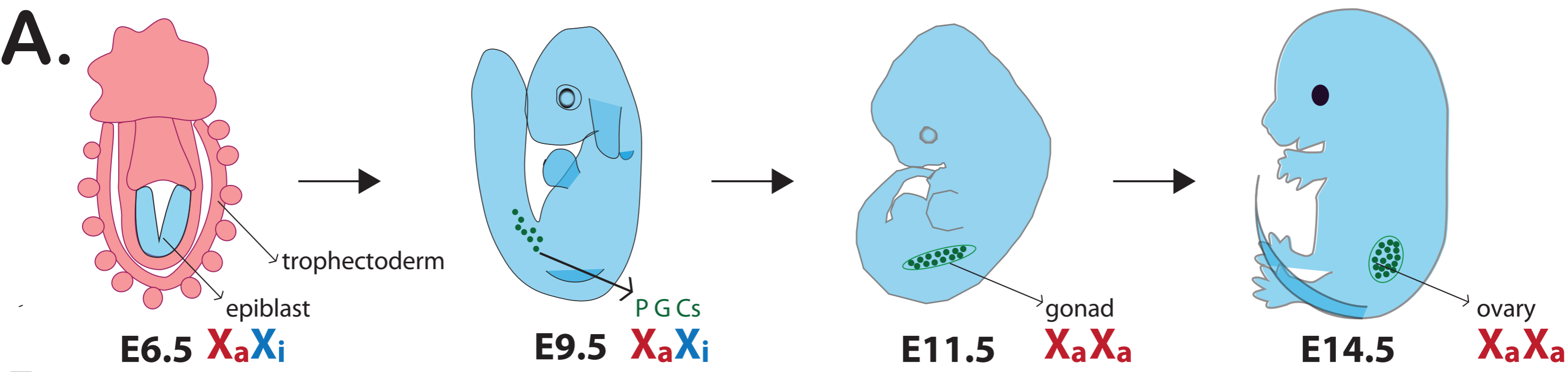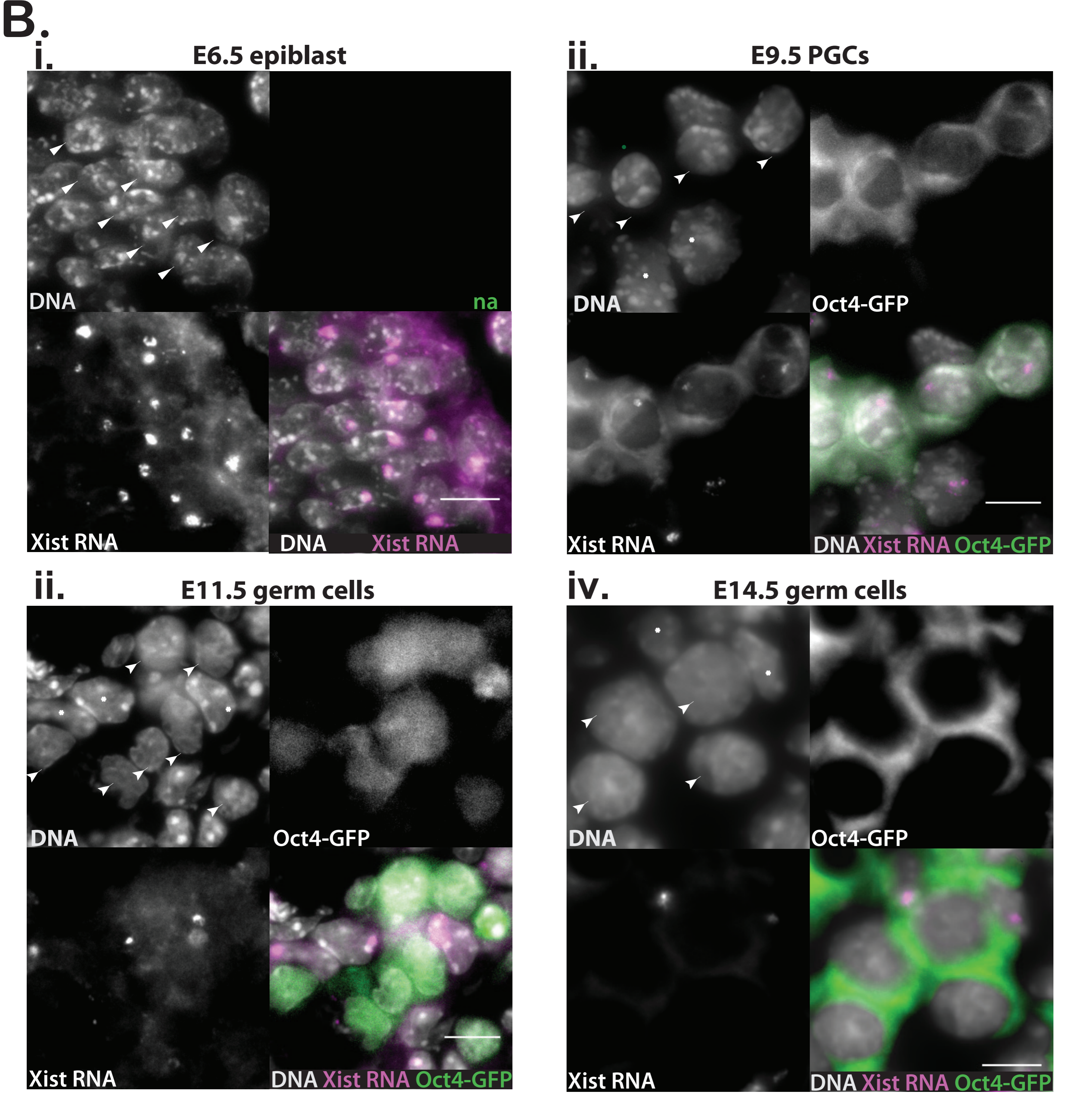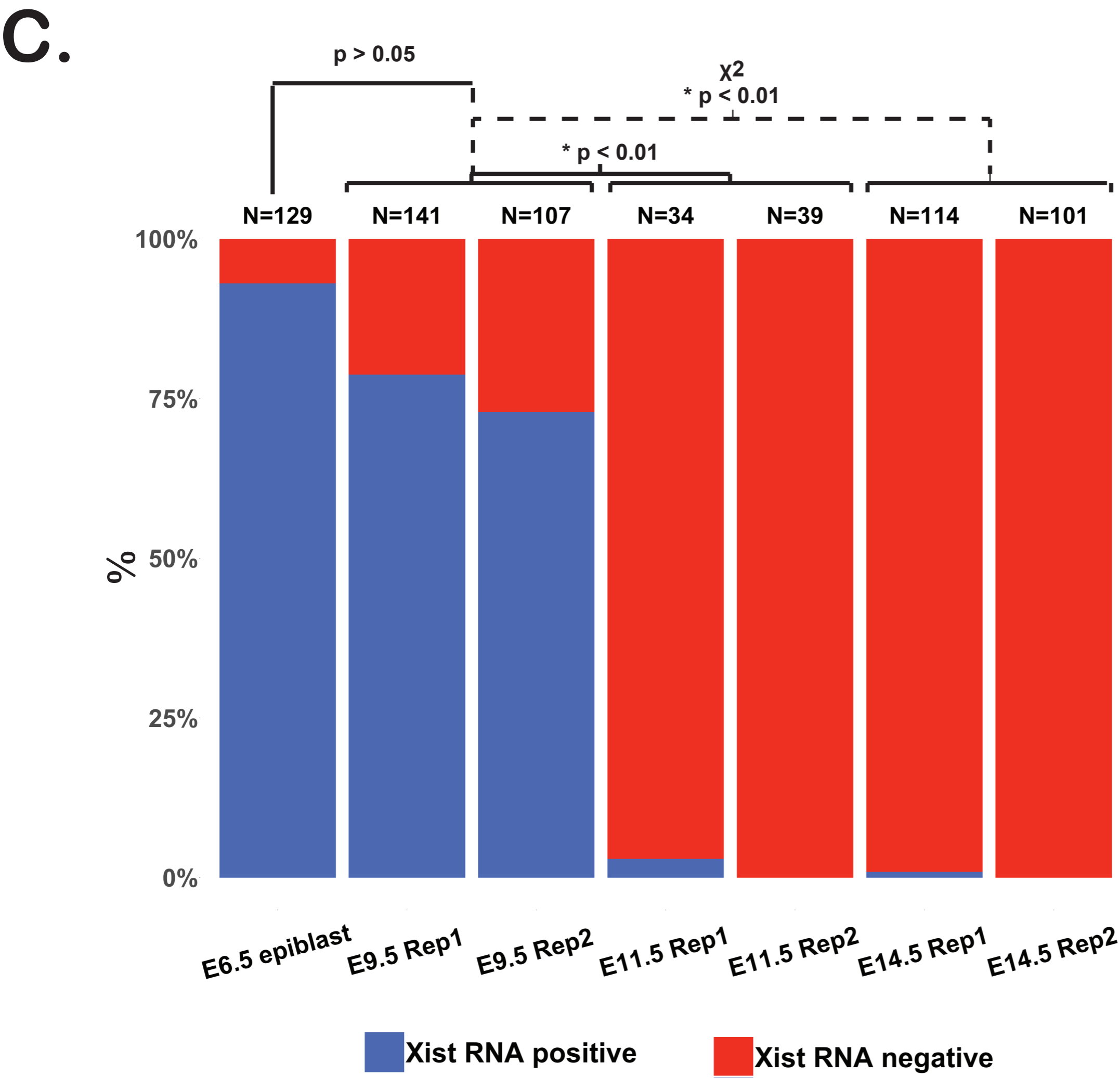

Supplementary Figure 5

A

Expression of X-linked and Autosomal Retrogene pairs (n=14)

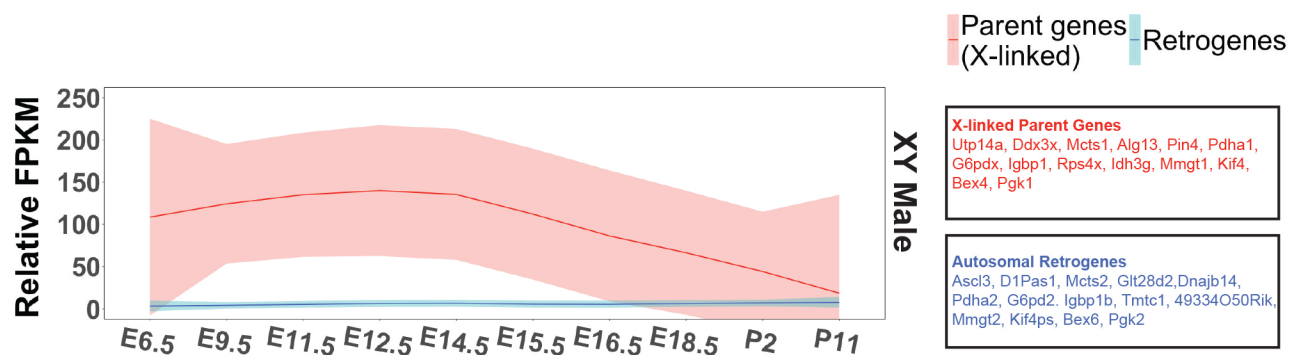

B

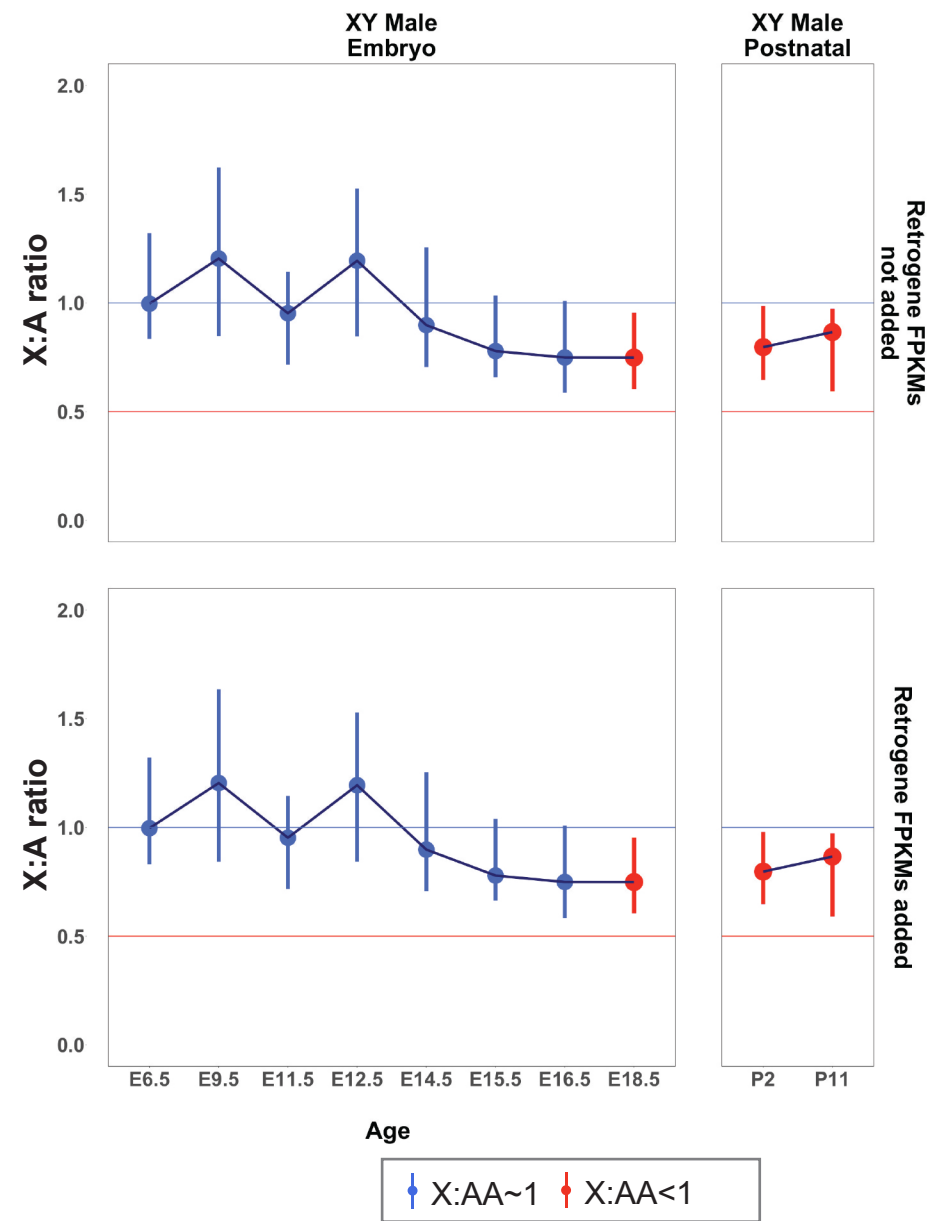

Supplementary Table 1

**Samples**	**No. of Reads**	**Mapped**	**Overall read mapping rate %**	**Aligned pairs**	**Concordant pair alignment rate %**
E9.5 XX F_GC_1	46050987	44723246	96.4	43258239	84.4
E9.5 XX F_GC_2	12272046	9243118	65.7	5454098	40.3
E9.5 XX F_SC_1	28386893	27003689	94.5	25648978	83
E9.5 XX F_SC_2	39713464	30943305	69.3	18167101	40.6
E9.5 XY M_GC_1	35965922	34418799	94.9	33117669	83.7
E9.5 XY M_GC_2	32802948	31399363	95	30300224	84.4
E9.5 XY M_GC_3	12132025	8094386	57.3	3901486	29.3
E9.5 XY M_GC_4	12043405	6643460	47.6	3540557	26.9
E9.5 XY M_SC_1	40406772	39405220	96.8	38192623	86
E9.5 XY M_SC_2	34355568	33592787	97	32540220	87.2
E9.5 XY M_SC_3	31512671	25420228	71.9	15780303	45.9
E9.5 XY M_SC_4	30045729	23386215	69.2	13729884	39
E9.5 XO F_GC_1	37914973	36484288	95.5	35032264	84
E9.5 XO F_GC_2	8963060	6278449	61.5	3509494	35.2
E9.5 XO F_SC_1	34802214	32533136	94.8	31498499	84.3
E9.5 XO F_SC_2	18415941	14009047	66.7	7804824	38.4
E9.5 XX M_GC_1	43964197	42393202	95.9	40807530	84.4
E9.5 XX M_GC_2	24306023	17943254	64.7	9708158	35.1
E9.5 XX M_SC_1	30513797	29558243	96.3	28457328	85
E9.5 XX M_SC_2	35128536	26768732	68.4	15890052	39.4
E11.5 XX F_GC_1	73772964	68591692	87	57518202	72.4
E11.5 XX F_GC_2	34691212	32350831	92	30206793	81.5
E11.5 XX F_GC_3	34632289	32189351	91.6	30797381	81.2
E11.5 XX F_GC_4	48389242	44559132	90.6	42332211	79
E11.5 XX F_SC_1	101557389	95261714	91.5	89686788	82.6
E11.5 XX F_SC_2	42827118	41192689	94.7	38825189	85.7
E11.5 XX F_SC_3	40658848	37854016	90.5	34249747	80.6
E11.5 XX F_SC_4	37396111	35343045	93.1	33890872	85.5
E11.5 XY M_GC_1	33854553	30771895	89	28341334	78.6
E11.5 XY M_GC_2	42203053	39697612	91.7	36424848	81
E11.5 XY M_SC_1	33674860	30983184	88.2	28109938	79.1
E11.5 XY M_SC_2	39919065	37140265	90.8	33765986	79.3
E11.5 XX M_GC_1	59879438	55967272	91.4	52626402	79.6
E11.5 XX M_GC_2	46968013	44044077	91.8	40631593	81.2
E11.5 XX M_SC_1	82063958	77603361	92.4	73359684	83.7
E11.5 XX M_SC_2	59220762	56706050	94.3	54415364	86.4
E12.5 XX F_GC_1	24889038	23011598	92	2238340	83.3
E12.5 XX F_GC_2	23877394	20770051	86.4	20198415	79.5
E12.5 XX F_GC_3	29595261	24276197	78.8	22034536	68.1
E12.5 XX F_GC_4	19184826	17045783	85.6	15572621	74
E12.5 XX F_SC_1	37656653	35296548	93.1	34420154	86.3

E12.5 XX F_SC_2	40697757	39031619	95.5	38245346	88.1
E12.5 XX F_SC_3	41140216	39440860	94.6	37960715	84.8
E12.5 XX F_SC_4	53578777	47892570	85.3	43364536	75.9
E12.5 XY M_GC_1	27962154	23874580	85	23160407	76.2
E12.5 XY M_GC_2	22147573	18487970	84	17824661	74.2
E12.5 XY M_GC_3	44748739	42812390	95.2	41848301	86.6
E12.5 XY M_GC_4	33340239	29120956	84.6	26900294	75.4
E12.5 XY M_GC_5	23070443	19383093	81.3	17899313	72
E12.5 XY M_GC_6	42084166	39484187	92.6	37948967	81.3
E12.5 XY M_SC_1	36271525	34502780	94.7	33733751	85.8
E12.5 XY M_SC_2	33733751	34252766	94.3	33407328	85.3
E12.5 XY M_SC_3	34724307	33354940	95.6	32706051	88
E12.5 XY M_SC_4	38771368	35673063	88.6	32723615	79.4
E12.5 XY M_SC_5	57732271	51842560	87.1	48262175	78.5
E12.5 XY M_SC_6	55926177	53325614	94.1	51207143	83.5
E12.5 XY M_SC_7	59473246	56641939	94	54381277	81.5
E12.5 XX M_GC_1	45296228	42615943	92.8	40838252	80.9
E12.5 XX M_GC_2	36182313	32421491	88.3	31111659	78.6
E12.5 XX M_SC_1	55321814	52929254	94.2	50560078	82.8
E12.5 XX M_SC_2	61733520	59190346	94.5	56729164	83.1
E12.5 XX M_SC_3	56729164	42666594	94.5	41018816	83.9
E14.5 XX F_GC_1	41018816	39702042	86.3	38449446	78.7
E14.5 XX F_GC_2	32428012	29868949	91.6	28981396	82.8
E14.5 XX F_GC_3	13545004	13036597	95.6	12738072	87.5
E14.5 XX F_GC_4	48548985	46968449	96.1	45963824	88.3
E14.5 XX F_GC_5	30482637	28968429	94.2	28226291	86.8
E14.5 XX F_SC_1	28684766	24567640	85.2	23829681	77.6
E14.5 XX F_SC_2	34532659	31053730	89.5	30288871	82.5
E14.5 XX F_SC_3	33131758	31843685	95.6	31183314	86.9
E14.5 XX F_SC_4	73635998	71187000	96	69555969	87.7
E14.5 XX F_SC_5	42493965	40454694	94.7	39059815	8.6
E14.5 XX F_Liver_1	48128368	46041897	94.8	44313655	85.5
E14.5 XX F_Liver_2	38376194	35319990	92.1	34088571	85.1
E14.5 XX F_Tail_1	37997850	36370339	95.4	35427812	89.7
E14.5 XX F_Tail_2	37995367	35728020	92.9	33729043	82.5
E14.5 XY M_GC_1	34616031	21053355	60.4	20284101	53.2
E14.5 XY M_GC_2	41199611	39244561	94.6	38318778	84.2
E14.5 XY M_GC_3	46688710	44218035	94	43053816	84.2
E14.5 XY M_SC_1	34496700	31823754	91.7	30836924	83.5
E14.5 XY M_SC_2	43689113	41979491	95.3	40924235	86.6
E14.5 XY M_SC_3	46679089	44764624	95	39750456	73.2
E14.5 XY M_Liver_1	34755780	32880912	94.4	31879420	88.6
E14.5 XY M_Liver_2	38195831	34512065	89.8	31837925	73.7
E14.5 XY M_Tail_1	35390365	33853408	95.4	33003213	89.8
E14.5 XY M_Tail_2	43067474	40882344	94.4	39158762	84.1
E14.5 XO F_GC_1	47098681	45385531	95.8	44086503	86.8

E14.5 XO F_GC_2	38041178	36440676	95.2	35366796	85.5
E14.5 XO F_SC_1	39239386	37949572	96	36834229	89.1
E14.5 XO F_SC_2	32480821	30990535	95	30115913	83.9
E14.5 XX M_GC_1	26601186	23440005	87.7	22831328	78.6
E14.5 XX M_GC_2	27935387	19958229	71	19273160	63.7
E14.5 XX M_GC_3	46478194	44289447	94.7	43271076	85.7
E14.5 XX M_GC_4	41324593	39049794	93.7	37993740	85
E14.5 XX M_SC_1	29361836	27950598	94.7	27192328	86
E14.5 XX M_SC_2	54993866	51931134	94	50491174	85.7
E14.5 XX M_SC_3	45769709	43878136	95.3	42913247	87.8
E14.5 XX M_SC_4	36990417	35140204	94.5	34341003	86.3
E15.5 XX F_GC_1	24562858	23583586	94.7	22454190	83.2
E15.5 XX F_GC_2	30126159	29140055	95.5	27844432	84.4
E15.5 XX F_SC_1	28514601	26796037	92.6	24837749	81.4
E15.5 XX F_SC_2	32215715	31082466	95.1	29495233	84.6
E15.5 XY M_GC_1	22999653	21811724	93.5	20599928	78.7
E15.5 XY M_GC_2	36692459	3535122	94.9	33615059	83.8
E15.5 XY M_SC_1	28423766	27239708	94.5	25683169	84
E15.5 XY M_SC_2	15787172	4072125	24.6	2597719	11.5
E15.5 XO F_GC_1	17209494	12005881	67.5	8841721	47
E15.5 XO F_GC_2	22322724	12475591	53.8	8482879	34.4
E15.5 XO F_SC_1	14871538	9258070	59.2	5902126	37.4
E15.5 XO F_SC_2	1387034	9561488	65.5	6188434	42.1
E15.5 XX M_GC_1	13084008	12278518	92.5	11616017	78.1
E15.5 XX M_GC_2	10748356	10009585	91.7	9456938	79.6
E15.5 XX M_SC_1	25704154	2462872	94.5	23249523	84.2
E15.5 XX M_SC_2	19185459	6091071	30.5	3946978	14
E16.5 XX F_GC_1	37033925	33920950	90.9	32977946	81.8
E16.5 XX F_GC_2	48735981	43025796	87.4	41461471	78.5
E16.5 XX F_GC_3	37249217	27382726	72.7	26304026	65.7
E16.5 XX F_SC_1	28931301	23476810	80.8	22971189	74.6
E16.5 XX F_SC_2	50347998	45699607	89.9	44207323	82.5
E16.5 XX F_SC_3	45708379	42300228	91.8	41085580	82.8
E16.5 XY M_GC_1	33701593	31124317	91.9	30236405	83
E16.5 XY M_GC_2	42243377	39675055	93.2	38653985	86
E16.5 XY M_GC_3	41666965	39587020	94.5	38658827	86.2
E16.5 XY M_GC_4	46730071	44367965	94.4	43350655	85.8
E16.5 XY M_GC_5	34303651	29570514	85.6	2853436	77.1
E16.5 XY M_GC_6	47255051	4340570	91	42002524	82.1
E16.5 XY M_SC_1	36359008	34550460	94.5	33712084	85.6
E16.5 XY M_SC_2	44795431	41823387	95	41823387	87.5
E16.5 XY M_SC_3	51011762	48915913	95.3	47809968	86.8
E16.5 XY M_SC_4	49824698	47769959	95.1	46595308	86.8
E16.5 XY M_SC_5	51821711	49803136	95.3	48617264	86.4
E16.5 XY M_SC_6	56368532	53670090	94.5	52440733	86.8
E16.5 XX M_GC_1	35841408	33691571	93.6	32812276	85.7

E16.5 XX M_GC_2	33405218	30398339	90.6	29462370	81
E16.5 XX M_SC_1	34665737	33574729	95.5	31965371	85.6
E16.5 XX M_SC_2	31308282	27756906	88.1	26779878	81
E18.5 XX F_GC_1	25377373	24415984	94.8	23255643	81.3
E18.5 XX F_GC_2	20402095	19550930	94.4	18628334	80.3
E18.5 XX F_SC_1	23560761	22543112	94.2	21299141	84.3
E18.5 XX F_SC_2	24122318	22962466	93.8	21753605	83.4
E18.5 XY M_GC_1	18906068	17823036	92.9	16820899	81
E18.5 XY M_GC_2	20423787	19512366	94.2	18504354	82.3
E18.5 XY M_SC_1	34665737	33574729	95.5	31965371	85.6
E18.5 XY M_SC_2	25638416	24846300	95.6	23680270	85.5
E18.5 XO F_GC_1	16431599	10664402	62.7	7646527	42.1
E18.5 XO F_GC_2	21088612	14130776	64.5	9692248	42.1
E18.5 XO F_GC_3	25136687	17018070	65.5	12382340	45.1
E18.5 XO F_SC_1	14820024	10121610	66.2	7137610	43.7
E18.5 XO F_SC_2	22662925	14779217	62.7	10044528	40
E18.5 XO F_SC_3	18261077	11505744	60.7	8028450	40.1
E18.5 XX M_GC_1	17191493	16403698	94	15494336	81.7
E18.5 XX M_GC_2	17703494	16090331	89.7	15273967	78.1
E18.5 XX M_SC_1	25718808	24946892	95.6	23724475	85.6
E18.5 XX M_SC_2	31407625	30531677	95.8	29127649	86.3
P2 XY M_GC_1	18583157	12706584	65.7	8872046	44.7
P2 XY M_GC_2	3997290	2322648	55.6	1551579	36.1
P2 XY M_GC_3	30046869	20103504	64.3	13936989	43.5
P2 XY M_GC_4	29534347	23537047	70.7	14431119	45.5
P2 XY M_GC_5	21985954	16737085	67.4	9891983	41
P2 XY M_GC_6	18359348	14248641	68.6	8494578	41.7
P2 XY M_SC_1	13302158	8466447	60.8	5696896	39.9
P2 XY M_SC_2	11518279	8115387	67.8	5799549	46.7
P2 XY M_SC_3	27273713	17882296	62.9	12163666	41.7
P2 XY M_SC_4	27862693	23080045	73.1	14502515	48.1
P2 XY M_SC_5	18359348	14248641	68.6	8494578	41.7
P2 XY M_SC_6	25726046	21123945	73.3	13449903	48.5
P11 XY M_GC_1	13302526	9393124	68.2	6085352	41
P11 XY M_GC_2	12677352	9263228	71	6280926	45.8
P11 XY M_GC_3	13472876	9366075	67.3	6048895	39.9
P11 XY M_GC_4	13198601	9443830	69.4	6261337	41.9
P11 XY M_SC_1	17570170	13575601	74.4	9659348	50
P11 XY M_SC_2	13375021	10206036	74.4	7300346	50.5
P11 XY M_SC_3	15625677	11454370	71.5	7888115	45.1
P11 XY M_SC_4	12599227	9124244	70.5	6163872	44.1
E6.5 XX F_1	17663893	1527982	8.8	202764	1.1
E6.5 XX F_2	16736555	4616881	26	660576	3.6
E6.5 XX F_3	21720331	4836561	20.8	794618	3.3
E6.5 XX F_4	16734020	7049084	41.5	3520223	19.3
E6.5 XY M_1	24876254	12463271	47.9	4257756	15.8

E6.5 XY M_2	30819788	19591773	60.8	9557711	28.4
E6.5 XY M_3	34004398	19434483	54.9	8412661	22.7
E6.5 XY M_4	23118529	8312502	34.5	5293986	19.6
E6.5 XY M_5	11416541	4552555	38.8	2460959	14.9
E6.5 XY M_6	19515827	4287107	20.5	900422	4.2

Supplementary Table 2

**Chrom_class**	**Condition**	**No. of genes FPKM>1**	**% Percentage of genes**
X-CH	E9.5.XY.M_SC	375	36.90944882
X-CH	E11.5.XY.M_SC	415	40.84645669
X-CH	E12.5.XY.M_SC	464	45.66929134
X-CH	E14.5.XY.M_SC	438	43.11023622
X-CH	E15.5.XY.M_SC	466	45.86614173
X-CH	E16.5.XY.M_SC	437	43.01181102
X-CH	E18.5.XY.M_SC	455	44.78346457
X-CH	P2_SC	480	47.24409449
X-CH	P11_SC	516	50.78740157
X-CH	E9.5.XX.F_SC	383	37.69685039
X-CH	E11.5.XX.F_SC	426	41.92913386
X-CH	E12.5.XX.F_SC	444	43.7007874
X-CH	E14.5.XX.F_SC	504	49.60629921
X-CH	E15.5.XX.F_SC	473	46.55511811
X-CH	E16.5.XX.F_SC	457	44.98031496
X-CH	E18.5.XX.F_SC	474	46.65354331
X-CH	E14.5.XX.F_liver	371	36.51574803
X-CH	E14.5.XY.M_liver	376	37.00787402
X-CH	E14.5.XX.F_tail	462	45.47244094
X-CH	E14.5.XY.M_tail	457	44.98031496
Autosome	E9.5.XY.M_SC	10424	47.31299927
Autosome	E11.5.XY.M_SC	11343	51.48420479
Autosome	E12.5.XY.M_SC	13129	59.5905955
Autosome	E14.5.XY.M_SC	12384	56.20915033
Autosome	E15.5.XY.M_SC	12814	58.16085694
Autosome	E16.5.XY.M_SC	12192	55.33769063
Autosome	E18.5.XY.M_SC	12326	55.94589688
Autosome	P2_SC	12701	57.64796659
Autosome	P11_SC	12615	57.25762527
Autosome	E9.5.XX.F_SC	10628	48.2389252
Autosome	E11.5.XX.F_SC	11536	52.36020334
Autosome	E12.5.XX.F_SC	12006	54.49346405
Autosome	E14.5.XX.F_SC	13387	60.76161946
Autosome	E15.5.XX.F_SC	13040	59.18663762
Autosome	E16.5.XX.F_SC	12665	57.4845679
Autosome	E18.5.XX.F_SC	13011	59.05501089
Autosome	E9.5.XX.M_SC	10364	47.04066812
Autosome	E14.5.XX.F_liver	11333	51.43881627
Autosome	E14.5.XY.M_liver	11359	51.55682643
Autosome	E14.5.XX.F_tail	12804	58.11546841
Autosome	E14.5.XY.M_tail	12720	57.73420479
